# Human Melanoma-Associated Mast Cells Display a Distinct Transcriptional Signature Characterized by an Upregulation of the Complement Component 3 That Correlates With Poor Prognosis

**DOI:** 10.3389/fimmu.2022.861545

**Published:** 2022-05-20

**Authors:** Rajia Bahri, Orsolya Kiss, Ian Prise, Karen M. Garcia-Rodriguez, Haris Atmoko, Julia M. Martínez-Gómez, Mitchell P. Levesque, Reinhard Dummer, Michael P. Smith, Claudia Wellbrock, Silvia Bulfone-Paus

**Affiliations:** ^1^ Lydia Becker Institute of Immunology and Inflammation, Faculty of Biology, Medicine and Health, University of Manchester, Manchester Academic Health Science Centre, Manchester, United Kingdom; ^2^ Division of Musculoskeletal & Dermatological Sciences, School of Biological Sciences, Faculty of Biology, Medicine and Health, University of Manchester, Manchester Academic Health Science Centre, Manchester, United Kingdom; ^3^ Department of Dermatology, Skin Cancer Center, University Hospital Zurich, University of Zurich, Zurich, Switzerland; ^4^ Division of Cancer Sciences, Faculty of Biology, Medicine and Health, University of Manchester, Manchester, United Kingdom

**Keywords:** mast cells, complement, IL-33, TGF-β, IL-1β

## Abstract

Cutaneous melanoma is one of the most aggressive human malignancies and shows increasing incidence. Mast cells (MCs), long-lived tissue-resident cells that are particularly abundant in human skin where they regulate both innate and adaptive immunity, are associated with melanoma stroma (MAMCs). Thus, MAMCs could impact melanoma development, progression, and metastasis by secreting proteases, pro-angiogenic factors, and both pro-inflammatory and immuno-inhibitory mediators. To interrogate the as-yet poorly characterized role of human MAMCs, we have purified MCs from melanoma skin biopsies and performed RNA-seq analysis. Here, we demonstrate that MAMCs display a unique transcriptome signature defined by the downregulation of the FcεRI signaling pathway, a distinct expression pattern of proteases and pro-angiogenic factors, and a profound upregulation of complement component C3. Furthermore, in melanoma tissue, we observe a significantly increased number of C3^+^ MCs in stage IV melanoma. Moreover, in patients, *C3* expression significantly correlates with the MC-specific marker *TPSAB1*, and the high expression of both markers is linked with poorer melanoma survival. *In vitro*, we show that melanoma cell supernatants and tumor microenvironment (TME) mediators such as TGF-β, IL-33, and IL-1β induce some of the changes found in MAMCs and significantly modulate C3 expression and activity in MCs. Taken together, these data suggest that melanoma-secreted cytokines such as TGF-β and IL-1β contribute to the melanoma microenvironment by upregulating C3 expression in MAMCs, thus inducing an MC phenotype switch that negatively impacts melanoma prognosis.

## Introduction

Among skin cancers, melanoma is the least common with the highest mortality rate, and its incidence is continuously increasing worldwide (1). Melanomas are among the most immunogenic tumors. Therefore, they have great potential to respond to immunotherapy. However, the acquisition of various suppressive mechanisms allows melanoma tumors to escape innate and adaptive immune detection (2). Despite the known efficacy of checkpoint inhibitors, a sizeable percentage of melanoma-affected patients do not benefit from single-agent therapy, prompting further investigation into whether their combination with other anticancer treatments could improve outcomes (3, 4). Furthermore, melanoma is one of the cancers that most commonly metastasize, and shows the highest propensity to develop metastases in the brain (5). Therefore, there is a need to explore adjuvant therapies that target skin immune-resident cells that promote barrier disruption, angiogenesis, and tumor cell migration.

Besides their conventional roles in allergy and host defense, mast cells (MCs) are key immunoregulatory cells that may also play an important, as-yet insufficiently explored, but therapeutically targetable role in tumor immunology (6). In mouse models, MCs play a pro-tumorigenic role and modulate melanoma tumor angiogenesis and tumor growth (7). Using a humanized mouse melanoma model, MC presence was associated with therapy resistance to anti-PD-1 and a decrease of CD8^+^ T cells. In this model, MC depletion induced tumor regression by anti-PD-1 treatment, showing that MCs are also able to play a role in therapy resistance (8).

It is thought that the wide range of bioactive molecules released by activated MCs impacts invasion, tumor-associated angiogenesis/lymphangiogenesis, and immune cells and, consequently, tumor growth and metastasis (6). However, their individual contributions in an oncological context remain opaque. Although increasing evidence shows that MCs consistently infiltrate tumors, it is yet unclear whether their role in tumor immunity is protective or harmful and to what extent this role is dictated by the local inflammatory tumor microenvironment (TME), namely, the site where non-cancerous cells, cancerous cells, and immune cells interact.

Melanoma cells are known to shape the microenvironment to escape immune surveillance and disseminate (2). In various malignant TMEs, including those fostered by surrounding melanoma, immunosuppressive cytokines, such as TGF-β, and the pro-inflammatory mediators of the IL-1 family, IL-1β and IL-33, have emerged as key facilitators of tumor growth, angiogenesis, and modulators of local immune responses (9–11).

Recently, several studies have demonstrated in TMEs increased concentrations of complement components that, by affecting immune cell responsiveness, contribute to tumor progression (12–14).

In this study, we aim to dissect the role of MCs in the modulation of melanoma growth. More specifically, we have characterized the transcriptional signature of MCs located at melanoma lesions to understand whether, within a malignant TME, MCs change their phenotype. We have demonstrated that MC numbers are increased in malignant (MEL: melanoma) and non-malignant (BCC: basal cell carcinoma) TMEs. However, the MC transcriptional signature in these two contexts significantly differs. While it is characterized by the expression of pro-inflammatory mediators in MCs from BCC lesions (BCCMCs), MEL-associated MCs (MAMCs) express pro-tumorigenic mediators such as pro-angiogenic factors, specific chemokines/cytokines (CCL2, IL-8, and IL-1β), and proteases. Interestingly, we observed that MAMCs are a source of complement C3 and that TGF-β, IL-1β, and IL-33 synergistically modulate the expression of C3 in MCs. Most importantly, we found a significant correlation between the increase of C3^+^ MC numbers, disease severity (melanoma stage IV), and poor prognosis. Thus, our findings demonstrate the plasticity of human MCs in a tumor context, show that specific mediators in the melanoma microenvironment dictate their phenotype, and underline the prognostic impact of C3^+^ MCs in melanoma.

## Materials and Methods

### Study Participants

All study participants provided written informed consent (UK NHS Human Research Authority, North West Greater Manchester West Research Ethics reference 17/NW/0328 and University of Zurich Hospital Biobank Ethics Committee reference EK647 and EK800, BASEC-Nr.PB-2017-00494). Thirteen subjects with basal cell carcinoma, six with melanoma, and eight healthy were recruited for the study. Native skin biopsies were collected and processed within 2–24 h for RNA-seq analysis. For immunohistochemistry, tissue microarrays of 148 biopsies were obtained from melanoma patients (93 biopsies comprising the tumor central area and 82 comprising the border) were used (**Supplementary Table 1**).

### Mast Cell Isolation From Human Skin Biopsies

After removing subcutaneous fat tissue, skin biopsies were chopped into small pieces and treated with collagenase 0.5 Wünsch unit/ml (Liberase**™**, Roche Applied Science, Mannheim, Germany) for 3–16 h. At complete digestion, cell suspensions were passed through 70-µm cell strainers and spun down. Cell suspensions were stained with Fc receptor blocking solution (Human TruStain FcX™), CD45 (2D1), anti-Human Lineage Cocktail [CD3 (UCHT1), CD14 (HCD14), CD19 (HIB19), CD20 (2H7), and CD56 (HCD56)], CD117 (104D2), and FcεRIα (AER-37) antibodies (all from BioLegend, San Diego, CA, USA) in FACS buffer [phosphate-buffered saline (PBS), 1% bovine serum albumin (BSA), 2 mM EDTA] for 30 min at 4°C. Then, cells were washed with PBS and incubated for 20 min with LIVE/DEAD™ blue viability dye to discriminate dead cells (LIVE/DEAD™ Fixable Blue Dead Cell Stain Kit, Life Technologies, Carlsbad, USA). Cells were washed, resuspended in FACS buffer, and sorted by flow cytometry using the FACS Aria cytometer. After exclusion of dead cells and doublets, CD45^+^Lin^−^CD117^+^ cells were gated and CD45^+^Lin^−^CD117^+^FcεRIα^+^ cells were sorted as MCs. The rest of the CD45^+^Lin^+^CD117^+^, CD45^+^Lin^+^CD117^−^, and CD45^+^Lin^−^CD117^−^ cells were sorted as CD45^+^ cells (**Supplementary Figure 1**). One thousand MCs were collected and stored at −80°C for further analysis.

### Sample Preparation for RNA-Seq

From MC pellets, total RNA was extracted and cDNA synthesized using the SMART-Seq^®^ kit (Takara Bio USA, Inc.) according to the manufacturer’s protocol. The amplified cDNAs were then submitted to the Genomic Technologies Core Facility (GTCF) and sequencing libraries generated using Nextera XT DNA Library Preparation Kits (Illumina, Inc.). The manufacturer’s protocol was modified to accommodate a reduced amount of starting material (100–150 pg). Adapter indices were used to multiplex the libraries, which were pooled prior to cluster generation using a cBot instrument. The loaded flow cell was then paired-end sequenced (76 + 76 cycles, plus indices) on an Illumina HiSeq 4000 instrument. Finally, the output data were demultiplexed (allowing one mismatch) and BCL-to-Fastq conversion was performed using Illumina’s bcl2fastq software.

### RNA-Seq Analysis

RNA samples were sequenced on the Illumina sequencing platform. Reads were filtered with Trimmomatic (v0.36). Filtered fastq files were aligned to the human GENCODE genome (GRCh38.v27) using STAR (v2.5.3). Filtered reads were then sorted and compressed and unaligned reads removed using samtools (v0.1.18). Aligned reads were then counted, normalized, and compared, respectively, using the Cuffquant, Cuffnorm, and Cuffdiff function of Cufflinks (v2.2.2). The enrichment of gene ontology terms was determined using Amigo2 (v2.4.6). Heat maps were visualized using Morpheus (https://software.broadinstitute.org/morpheus). Genes were clustered using one minus Pearson correlation k-means clustering. The network visual analysis of enriched gene ontology categories was generated using the NetworkAnalyst website (https://www.networkanalyst.ca/NetworkAnalyst).

### Human Blood-Derived Mast Cell Generation

Peripheral blood from healthy donors was purchased from the National Health Service blood bank (Manchester) and used in accordance with the University of Manchester Research Ethics Committee (UREC ref 2018-2696-5711). Peripheral blood mononuclear cells (PBMCs) were isolated using Ficoll-Paque Plus (GE Healthcare, Uppsala, Sweden) from healthy donor buffy coat or apheresis cones provided by the National Health Service blood bank (Manchester, UK), and human MCs derived from blood progenitors were generated, as previously described (15). Briefly, CD117^+^ progenitor cells were purified from PBMCs by using a positive selection kit (Miltenyi Biotec, Bergisch Gladbach, Germany) following the manufacturer’s instructions. Progenitor cells were cultured in Serum-Free StemSpan Medium (STEMCELL Technologies, Canada) supplemented with 100 U/ml penicillin (Invitrogen), 100 μg/ml streptomycin (Invitrogen), human IL-3 (10 ng/ml, PeproTech, USA), human stem cell factor (100 ng/ml, PeproTech, USA), and human IL-6 (50 ng/ml, PeproTech, USA). After 4 weeks, cells were progressively transferred to culture medium 2 containing Iscove’s modified Dulbecco medium with GlutaMAX-I, 50 μmol/L β_2_-mercaptoethanol, 0.5% BSA, 1% Insulin-Transferrin-Selenium (Life Technologies, USA), 100 U/ml penicillin, 100 μg/ml streptomycin, human stem cell factor (100 ng/ml), and human IL-6 (50 ng/ml). After 8 to 10 weeks of culture, the cells were tested for maturity and more than 90% were found positive for CD117 and FcϵRIα.

### Cell Culture Supernatants

Normal human melanocytes (NHMs) were cultured in M254 medium, supplemented with 1% (v/v) human melanocyte growth supplement (HMGS2), 100 U/ml penicillin, and 100 μg/ml streptomycin. The melanoma cell line BRAF^V600E^ mutated A375 and RPMI-7951 were cultured in Dulbecco’s modified Eagle’s medium (DMEM) supplemented with 10% fetal bovine serum and 100 U/ml penicillin and 100 μg/ml streptomycin. NHMs were from Cascade Biologics and A375 and RPMI-7951 from the ATCC. Due to the different cell growth, cells at confluence were cultured for 24 h with Dulbecco’s modified Eagle’s medium supplemented with 10% fetal bovine serum (FCS) and 100 U/ml penicillin and 100 μg/ml streptomycin, and then the cell culture supernatants were harvested and concentrated 10 times using the Amicon^®^ Ultra centrifugal filter 10K (Merck Millipore, Ireland) and stored at −80°C. Taking into account that FCS could have an effect on MCs (16), controls were incubated with a medium containing 10 times concentrated FCS for all experiments that employed cell culture supernatants.

### Human Mast Cell Culture

Mast cells were seeded at 5 × 10^5^ cells per ml with 50% (v/v) medium 2 and cell culture supernatants. NHM, A375, and RPMI-7951 cell culture supernatants were used as well as medium alone [50% (v/v) medium 2 and supplemented DMEM] for control. In some cases, MCs were incubated for 30 min with neutralizing antibodies or appropriate isotype control antibodies before treatments. The following blocking antibodies were used with their respective isotype controls: anti-human IL-33 (50 μg/ml) (AF3625), goat IgG control (AB-108-C), anti-human IL-1β (50 μg/ml) (clone: 8516, MAB201), and mouse IgG1 isotype control (MAB002R) antibodies all from R&D Systems, Mineapolis, USA and anti-human TGF-β1 (100 μg/ml) (clone: 1D11.16.8) and mouse IgG1 isotype control (clone: MOPC-21) from Bio X Cell, USA.

### Real-Time PCR

Mast cells (3 × 10^5^ at 5 × 10^5^ cells/ml) were cultured with 50% (v/v) medium 2 and 10× concentrated cell culture supernatant. After 48 h, MC supernatants were harvested and total RNA was extracted from the cell pellets using the RNeasy Micro kit (Qiagen, Manchester, UK) according to the manufacturer’s instructions. Complementary DNA was synthesized using the Tetro complementary DNA synthesis kit (Bioline, Wokingham, UK). To quantify the level of gene expression, a real-time quantitative PCR was performed using StepOne real-time PCR system (Applied Biosystems, Warrington, UK) with SYBR Green fast dye advance master mix (Applied Biosystems). Gene names and primer sequences used in this study are shown in **Supplementary Material Table 2**, and the following primers from Eurogentec were used: carboxypeptidase A3 (*CPA3*), tryptase (*TPSAB*), chymase (*CMA1*), *C3*, *C5*, *IL1B*, *FCERIA*, *MS4A2*, *C1R*, *C1S*, and *KIT*. Samples were run using the StepOne Plus Real-Time PCR system and associated software (Applied Biosystems); relative expression was quantified against the housekeeping genes *H3-3B*, *PPIA*, and *18S*.

### Flow Cytometry

#### Membrane Staining

Cells were incubated with Fc receptor blocking solution (Human TruStain FcX™) and stained with anti-human CD63 antibody (clone H5C6, BioLegend) in FACS buffer for 30 min at 4°C. Cells were then washed with PBS and incubated for 20 min with LIVE/DEAD™ blue viability dye (LIVE/DEAD™ Fixable Blue Dead Cell Stain Kit, Life Technologies, Carlsbad, USA). After washing, cells were fixed with 4% formaldehyde solution, resuspended in FACS buffer, and analyzed with an LSRII or LSRFortessa flow instrument (BD Biosciences). Data were analyzed using the FlowJo software (Tree Star Inc.) and degranulation was measured as a percentage of CD63^+^ cells.

#### Intracellular Phosflow Staining

MCs (3 × 10^5^ at 5 × 10^5^ cells/ml) were incubated for 90 min with 50% (v/v) medium 2 and NHM, A375, and RPMI-7951 cell culture supernatants, and recombinant human TGF-β1 at 5 ng/ml (PeproTech, USA) was used as positive control and medium for negative control. At the end of the treatment, MCs were fixed immediately with one volume of prewarmed BD Phosflow™ Fix Buffer I (557870, BD Biosciences, San Diego, USA) at 37°C for 15 min. Cell pellets were permeabilized with cold BD Phosflow™ Perm Buffer III (558050, BD Biosciences, San Diego, USA) for 30 min on ice. Cells were washed twice and stained with Fc receptor blocking solution and anti-Smad2 (pS465/pS467)/Smad3 (pS423/pS425) (clone: O72-670, BD Biosciences, San Diego, USA) antibody or isotype control for 1 h in FACS buffer. Cells were washed and resuspended in FACS buffer before flow analysis.

### β-Hexosaminidase Release Assay

Cell supernatants and pellets were separated by centrifugation. Cell pellets (25,000 cells) were lysed in 50 µl 1% Triton X-100 media 2. β-Hexosaminidase activity was measured in supernatants (50 µl) as well as in the cell pellets by adding 100 µl of β-hexosaminidase substrate and 10 mM *p*-nitrophenyl *N*-acetyl-beta-D-glucosaminide (Sigma-Aldrich, Saint Louis, USA) in 0.1 M Na_2_HPO_4_ buffer (pH 4.5) for 2 h at 37°C. Before reading the optical density at the wavelength of 405 nm, the reaction was stopped by adding 100 µl of 0.2 M glycine buffer (pH 10). MC degranulation was assessed as % release of total β-hexosaminidase.

### ELISA

After treatment, MC pellets were separated from the supernatants, washed two times in 1× PBS, and lysed in an equal volume (75 µl) as the supernatant of 1× PBS, 1% (v/v) Triton X-100, 2 mM EDTA, and 1× protease inhibitor cocktail. We measured in MC pellets and supernatants the complement C3 component concentration using a human complement C3 ELISA kit (ab108823, Abcam, UK) and IL-33 using the human IL-33 DuoSet ELISA (R&D Systems, Mineapolis, USA). Both C3 and IL-33 were measured according to the manufacturer’s instructions.

### Cytometric Bead Array

After treatment, MC pellets were separated from the supernatants, washed two times in 1× PBS, and lysed in an equal volume as the supernatant (75 µl) of 1× PBS, 1% (v/v) Triton X-100, 2 mM EDTA, and 1× protease inhibitor cocktail. IL-8/CXCL-8, CCL2/MCP-1, IL-1β, TGF-β, anaphylatoxin C3a, C4a, and C5a were quantified by a BD cytometric bead array (CBA, BD Biosciences, San Diego, USA) multiplex kit following the manufacturer’s protocol and analyzed using FACSVerse flow cytometer. The analysis was performed using the FCAP Array™ Software v3.0.

### C3 Cleavage Assay

MCs (5 × 10^4^ cells) were washed two times in 1× PBS, pH 7.2 and resuspended in 50 μl of 1× PBS, pH 7.2. To disrupt the cellular membrane, MCs were sonicated for 3 min (3 cycles of 1 min) in an ultrasonic bath. In some conditions, MC tryptase activity was inhibited either by adding 100 μM of the tryptase-specific inhibitor APC366 (R&D Systems) 30 min before the assay or either by treating MCs 2 h at 56°C as previously described. Human serum-purified C3 (Merck, USA) 500 ng/ml was incubated 20 min at 37°C with 5 × 10^4^ MCs in a final volume of 100 μl of 1× PBS, pH 7.2. The reaction was stopped by adding 10 μl of 1 N HCl and the generated C3a was then quantified by CBA.

### Immunohistochemistry

Tissue microarrays were prepared from formalin-fixed paraffin-embedded samples containing mostly metastatic melanoma tumors from stage III and IV patients who were treated at the University Hospital of Zurich after approval of the local ethics and scientific committees. The methods and cohort descriptions have been previously published (17, 18). Five-micrometer paraffin-embedded skin sections were de-paraffinized in xylene (2 × 5 min) and rehydrated in descending grades of alcohol (2× 100%, 95%, 70%, water; 2 min/step). Rehydrated slides were boiled in sodium citrate buffer (10 mM, pH 6) for 20 min for antigen retrieval and blocked in blocking buffer (10% normal goat serum in PBS–Tween 20, 0.1% v/v) for 30 min at room temperature. After the blocking steps, sections were incubated with primary antibodies diluted in blocking buffer for 1 h at room temperature. Anti-tryptase antibody (monoclonal mouse, Abcam, #ab2378, 1:1,000) was applied with melan A (monoclonal rabbit, Abcam, #ab51061, 1:200) and complement 3 (C3) (polyclonal chicken, Sigma, #GW20073F, 1:400) or C3/C3a (polyclonal chicken, Abcam, #ab48580, 1:400) antibodies as appropriate. Primary antibodies were skipped for negative control. Following exposure to primary antibodies, slides were washed three times in PBS–Tween 20 (0.1% v/v) and incubated with the secondary antibodies diluted in 1:200 in blocking buffer for 30 min at room temperature. Goat anti-chicken AlexaFlour 488 (Invitrogen, #A11039) was combined with anti-rabbit AlexaFlour 555 (Abcam, #ab150086) and anti-mouse AlexaFlour 647 (Abcam, #ab150117), respectively. Sections were washed and mounted using Fluoroshield mountant containing 4′,6-diamidino-2-phenylindole (Abcam, UK) prior to image analysis.

Images were acquired on a 3DHISTECH Pannoramic-250 microscope slide scanner using a [×20/0.80 Plan Apochromat] objective (Zeiss) and the DAPI, FITC, Cy3, and Cy5 filter sets. Images were then processed and analyzed using HistoQuant application (3DHISTECH). Snapshots of the slide scans were taken using the CaseViewer software (3DHISTECH). Images were also collected on a Zeiss Axioimager.D2 upright microscope using a ×40/0.5 EC Plan-Neofluar objective and captured using a Coolsnap HQ2 camera (Photometrics) through Micro-Manager software v1.4.23. Specific bandpass filter sets for DAPI, FITC, Cy3, and Cy5 were used to prevent bleed-through from one channel to the next. Images were then processed and analyzed using ImageJ software (https://imagej.nih.gov/ij/download.html).

### The Cancer Genome Atlas Melanoma Patient Cohort Analysis

The cohort dataset with 426 melanoma RNA-sequencing data and clinical information was obtained from The Cancer Genome Atlas (TCGA) (https://portal.gdc.cancer.gov/). Kaplan–Meier analysis of the TCGA melanoma patient cohort was performed (19). Differences in overall survival for patients whose tumors express high or low levels of *TPSAB1* and *C3* transcripts were determined. The data were analyzed for mRNA expression (*z*-score 2.0) in cBioPortal (20) and survival data were extracted and analyzed in GraphPad Prism.

### Statistical Analysis

All data are presented as the mean ± SEM. Statistically significant differences between two groups were calculated by using the non-parametric Mann–Whitney test. Unless indicated in the figure legends, for data consisting of more than two groups, statistically significant differences were calculated by using the one-way analysis of variance (ANOVA) and Kruskal–Wallis with Dunn’s *post-hoc* comparison test (Prism software; GraphPad Software v 8-9.00 Inc.) with *p < 0.05, **p < 0.01, ***p < 0.001, ***p < 0.001, and ****p < 0.0001 considered significant.

### Study Approval

All study patients provided written informed consent prior to participation (UK NHS Human Research Authority, North West Greater Manchester West Research Ethics reference 17/NW/0328 and University of Zurich Hospital biobank Ethics Committee reference EK647 and EK800, BASEC-Nr.PB-2017-00494).

## Results

### Increased Mast Cell Numbers in Melanoma and Basal Cell Carcinoma Lesions

**Figure 1:**
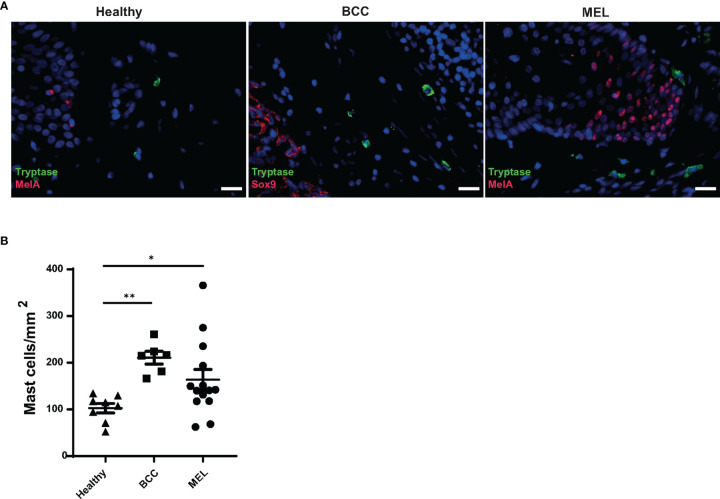
Increased mast cell numbers in melanoma and basal cell carcinoma lesions. **(A)** Mast cells present in skin biopsies from healthy, basal cell carcinoma (BCC), and melanoma subjects. Immunohistochemistry was used to identify mast cells (MCs) (tryptase, green), melanocytes (Mel A, red), and BCC cells (SOX9, red) in the skin. Nuclear staining was performed with DAPI (blue). Scale bars represent 20 μm. **(B)** Numbers of MCs were determined in three different biopsy groups: healthy skin (*n* = 8), melanoma (*n* = 14), and BCC (*n* = 6) by immunohistochemistry using 5-μm paraffin sections and anti-tryptase antibody staining and expressed as mast cells/mm^2^. Dots represent the mean cell density of 10 areas of interest (AOI) per tissue section. Significance was calculated with Kruskal–Wallis test with Dunn’s multiple comparison. *p > 0.05, **p > 0.01.

The accumulation of MCs in tumor tissue indicates their involvement in shaping the characteristics of the TME and exerting an important function in tumor biology (21). To investigate whether the presence of tumors influences MC density in the skin, we identified MCs by histochemistry with staining for the MC marker tryptase (TPSAB1) and compared cell numbers in lesions of melanoma (*n* = 14) with the ones in slow-growing, non-metastatic basal cell carcinoma (BCC) (*n* = 6) and healthy skin (*n* = 8). MCs were found located up to ∼900 μm from the tumor border and occasionally observed in close contact with the tumor. While MCs were rarely found within the tumor tissue, they were mostly distributed in the surrounding stroma. An increased number of peri-tumoral MCs was observed in melanoma (mean: 163 MCs/mm^2^, SD: 81) and in BCC (mean: 210 MCs/mm^2^, SD: 33) lesions compared with healthy skin (mean: 102 MCs/mm^2^, SD: 28) (**Figures 1A, B**), suggesting the involvement of MC products in conditioning the TME of both skin tumors.

### Melanoma-Associated Mast Cells Display a Distinct and Unique Transcriptional Signature

**Figure 2:**
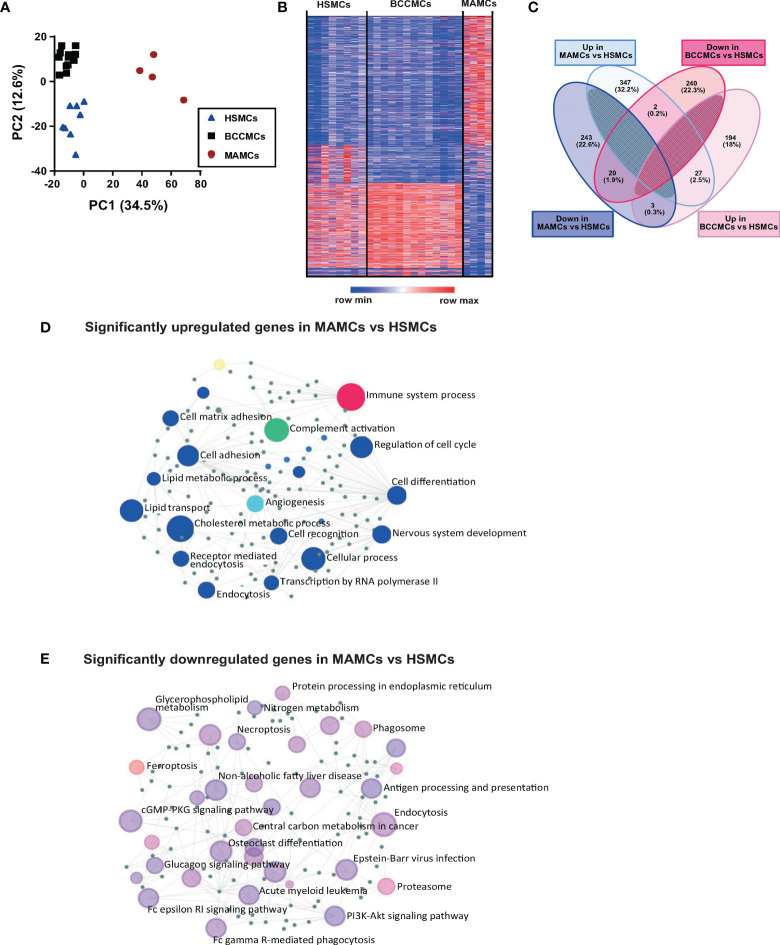
Melanoma-associated mast cells (MAMCs) display a distinct and unique transcriptional signature. Mast cells were isolated by flow cytometry from 8 healthy skin (HSMCs), 13 BCC (BCCMCs), and 4 melanoma (MAMCs) biopsies. The RNA was extracted and the Illumina RNA-seq analysis was performed. **(A)** Principal component analysis (PCA) biplot of gene expression data where each biopsy condition is represented by a symbol: HSMCs in blue triangles, BCCMCs in black squares, and MAMCs in red circles. **(B)** Heat maps of changes in expression values of genes in MCs isolated from healthy skin, BCC, and MEL; red to blue scale bar indicated the *z*-score of fold change per genes (red indicates higher *z*-score, blue indicates lower *z*-score). Each row indicates an individual gene and each column a sample. **(C)** Venn diagram displays significantly up- and downregulated transcripts in BCCMCs (pink) and MAMCs (blue) versus HSMCs. Numbers represent gene numbers in different groups. **(D)** Network visual analysis of enriched gene ontology categories for significantly upregulated and downregulated **(E)** genes in MAMCs compared with HSMCs. Each circle represents a group of genes from the same biologic process.

In the skin, resident MCs patrol the tissue microenvironment and sample environmental antigens (22). These cells are possibly among the first immune cells to modify their functionality and phenotype upon tumor development. To understand whether the MCs residing in the TME are dysfunctional, we have investigated the transcriptional signature of MAMCs and compared them with MCs isolated from BCC lesions (BCCMCs) and healthy human skin (HSMCs). To this purpose, MCs (CD45^+^Lin^−^CD117^+^FcϵRIα^+^) were purified from biopsies of melanoma (*n* = 4) and BCC (*n* = 13) patients and healthy volunteers (*n* = 8) by flow cytometry sorting. For comparison, we also sorted CD45^+^ cells (**Supplementary Figures 1A, B**). RNA-seq analysis was performed on 1,000 isolated MCs. PC1 and PC2 in the principal component analysis (PCA) showed a distribution into three separate groups for HSMCs, BCCMCs, and MAMCs (**Figure 2A**). While the expression of individual genes was more similar between HSMCs and BCCMCs, the gene expression pattern of MAMCs differed profoundly from HSMCs (**Figure 2B**). In MAMCs, 347 transcripts were found significantly upregulated and 243 transcripts were found downregulated compared with HSMCs (*q* value < 0.05) (**Figure 2C** and **Supplementary Table 3**). Only a few transcripts were found commonly upregulated (23) or downregulated (20) in both MAMCs and BCCMCs relative to HSMCs (**Figure 2C** and **Supplementary Table 3**). CD45^+^ cells sorted from melanoma, BCC, or healthy skin showed a less clear PCA separation distribution and fewer differentially expressed genes (**Supplementary Figures 2A–C**). The top 50 highly expressed transcripts in skin MCs are listed in **Supplementary Table 4**.

Gene ontology enrichment analysis of significantly up- and downregulated transcripts for biological functions in MAMCs shows enrichment in immune system, angiogenesis, cell matrix, and complement activation processes (**Figure 2D**). In contrast, transcripts associated with the process of MC degranulation, namely, the high-affinity FcϵRI signaling pathway that mediates MC degranulation *via* IgE binding and allergen cross-linking, were significantly downregulated in MAMCs compared with HSMCs (**Figure 2E**). Gene set enrichment analysis (GSEA) was performed for genes belonging to the complement cascade and FcϵRI signaling pathway in HSMCs and MAMCs. A significant upregulation of the complement-related and a downregulation of the FcϵRI signaling pathway gene set were observed in MAMCs compared with HSMCs. Thus, these findings confirmed the results obtained with the gene ontology enrichment analysis (**Supplementary Figures 3C, D** **)**.

**Figure 3:**
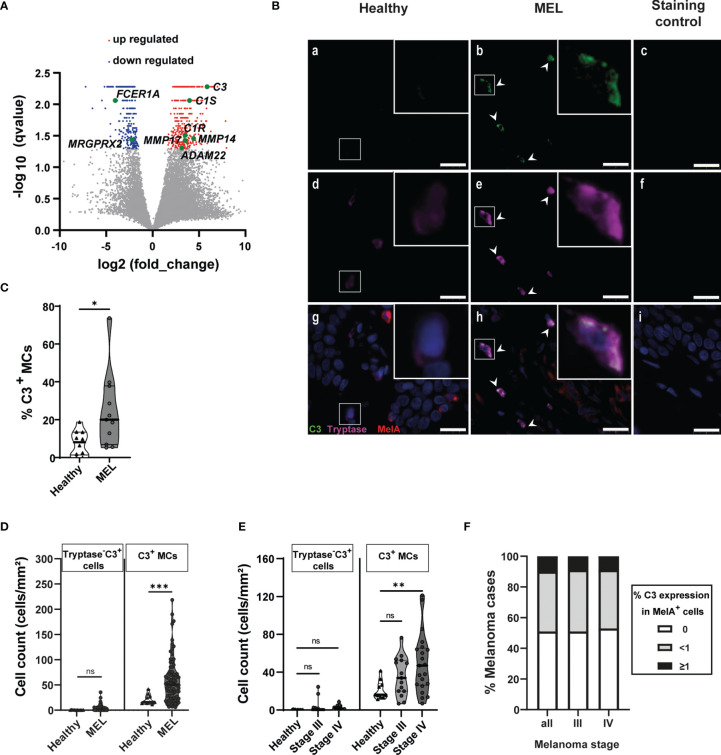
Melanoma-associated mast cells express C3. **(A)** Volcano plots display differential gene expression between MAMCs and HSMCs. Each gene is symbolized by a dot: red dots indicate significantly upregulated genes and blue dots indicate significantly downregulated genes in MAMCs. **(B)** Stain of fixed healthy (Healthy) and melanoma (MEL) skin biopsies. Representative images of immunofluorescence staining of tryptase (magenta) and co-expressed complement 3 (C3) (green) in MAMCs (b, e, h) compared with HSMCs (a, d, g). Control: no primary antibody added (c, f, i). Nuclear staining was performed with DAPI (blue). Scale bars represent 20 μm. White arrows show the C3^+^ MCs. White squares mark the areas shown at a higher zoom in the upper right. Tryptase^+^ and tryptase^+^ C3^+^ MCs were counted. **(C)** Violin plots show the calculated percentage of tryptase^+^ C3^+^ MCs in healthy (*n* = 10) and MEL skin biopsies (*n* = 11). Data are displayed as median (heavy line) with interquartile range. Statistical comparisons were performed using Mann–Whitney *U*-test. *p = 0.0155. **(D)** Violin plots display C3^+^ tryptase^−^ and C3^+^ tryptase^+^ (C3^+^ MCs) cell numbers in healthy skin samples (healthy, *n* = 10) and in melanoma borders (MEL, *n* = 82). Results are shown as mean with quartile range. Statistical comparisons between healthy and MEL cell counts were performed using Mann–Whitney *U*-test, ***p < 0.001, ns = not significant. **(E)** Violin chart comparing C3^+^ tryptase^−^ and C3^+^ tryptase^+^ (C3^+^ MCs) cell numbers in healthy skin (*n* = 10) and melanoma at stage III (*n* = 15) and stage IV (*n* = 20). Data are displayed as median with interquartile range. One-way ANOVA statistical test was used, **p < 0.01, ns = not significant. **(F)** C3 and MelA immunohistochemistry staining was performed on 93 biopsies located in the central melanoma area, and MelA^+^ and C3^+^ cells were counted. C3 expression levels were defined as three groups: 0, no C3 expression (white bars); <1, less than 1% of C3^+^ MelA^+^ cells (gray bars); and ≤1, equal or greater than 1% of C3^+^ MelA^+^ cells (black bars). Chart shows the percentage of C3^+^ MelA^+^ cells in all melanoma biopsies analyzed (total, *n* = 93), stage III melanoma (stage III, *n* = 24), and stage IV melanoma (stage IV, *n* = 31). The polyclonal chicken antibody from Abcam, #ab48580, was used for C3/C3a staining in **(B**, **D**–**F**), and in **(C)**, the polyclonal chicken antibody from Sigma, #GW20073F, was used for C3 staining.

In particular, the FcεRIα (*FCER1A*) (HSMCs: 249 FPKM, MAMCs: 15 FPKM, fold decrease: 16, *q* value: 0.008) together with its β (*MS4A2*) (HSMCs: 289 FPKM, MAMCs: 109 FPKM, fold decrease: 2.6, *q* value: 0.12) and γ chains (*FCERIG*) (HSMCs: 987 FPKM, MAMCs: 286 FPKM, fold decrease: 3.4, *q* value: 0.05), as well as transcripts related to FcϵRI signaling such as the tyrosine-kinase SYK (HSMCs: 12.5 FPKM, MAMCs: 3.5 FPKM, fold decrease: 3.5, *q* value: 0.045) and the serine/threonine kinase AKT1 (HSMCs: 44.2 FPKM, MAMCs: 5.2 FPKM, fold decrease: 8.4, *q* value: 0.005), was downregulated in MAMCs (**Figure 3A**, **Supplementary Figure 3A** and **Supplementary Tables 5, 6**). In addition, transcripts coding for the mas-related G-protein-coupled receptor member X2 (*MRGPRX2*) (HSMCs: 41 FPKM, MAMCs: 8.5 FPKM, fold decrease: 4.8, *q* value: 0.03), whose ligation by a wide range of neuropeptides and active compounds induce MC degranulation, were reduced in MAMCs (**Figure 3A**). Interestingly, compared with HSMCs, transcripts related to FcϵRI-mediated degranulation are upregulated in BCCMCs (*FCER1A* in HSMCs: 249 FPKM, BCCMCs: 562 FPKM, fold increase: 2.2, *q* value: 0.02) (**Supplementary Figure 3A** and **Supplementary Tables 5, 6**).

While MAMCs display upregulation of angiogenesis-related transcripts (**Figure 2D** and **Supplementary Figure 4A**), their protease profile is altered with a significant increase in several metalloprotease (MMP) transcripts, e.g., *MMP14* (HSMCs: 0.89 FPKM, MAMCs: 18.98 FPKM, fold increase: 21.4, *q* value: 0.034) and *MMP17* (HSMCs: 1.4 FPKM, MAMCs: 15.9 FPKM, fold increase: 11.3, *q* value: 0.037) (**Figure 3A**), and a reduction in tryptase (*TPSAB*, HSMCs: 7,561 FPKM, MAMCs: 2,036 FPKM, fold decrease: 3.7, *q* value: 0.17), chymase (*CMA1*, HSMCs: 711 FPKM, MAMCs: 255 FPKM, fold decrease: 2.78, *q* value: 0.09), and carboxypeptidase A3 (*CPA3*, HSMCs: 861 FPKM, MAMCs: 629 FPKM, fold decrease: 1.3, *q* value: 0.58) expression (**Supplementary Figure 4B** and **Supplementary Table 9**).

Furthermore, MAMCs exhibited a significant increase in the expression of the complement cascade genes. The significant upregulation of complement *C3* (HSMCs: 6.34 FPKM, MAMCs: 371.8 FPKM, fold increase: 58.6, *q* value: 0.005) and upstream components *C1R* and *C1S* (**Figures 2D**, **3A**, **Supplementary Figure 3B** and **Supplementary Table 7**) and the increase of several complement-related transcripts involved in the classical complement pathway *(C1QA*, *C1QB*, *C1QC*) and the lectin pathway (MASP1, MASP2) were accompanied by the downregulation of complement inhibitory receptor/mediator transcripts, *CD55*, *CFHR2*, *CD59*, and *CD46* (**Supplementary Figure 3B** and **Supplementary Table 7**). While single complement system components were modulated in CD45^+^ cells from healthy skin, BBC, and melanoma, a general upregulation of the complement cascade appears to be a unique characteristic of MAMCs (**Supplementary Figure 3B** and **Supplementary Table 8**).

To confirm the upregulation of the complement cascade, we investigated the tissue expression of C3 since this component plays a central role in the activation of the complement system. Triple immuno-staining detecting melan-A (MelA) (tumor area), tryptase (MAMCs), and C3 was performed on slides obtained from melanoma lesions and healthy skin biopsies and tryptase^+^ C3^+^ MAMCs were counted. In melanoma, 24% of MAMCs (SD: 20, *n* = 11) expressed C3 compared with 7% of C3^+^ MCs in healthy skin (SD: 6, *n* = 10) (**Figures 3B, C** **)**. A significant increase in C3^+^ MAMC (tryptase^+^ C3^+^) numbers was observed in melanoma (mean: 58.72 cells/mm^2^, SD: 43.64, *n* = 82) compared with tryptase^+^ C3^+^ healthy skin MCs (mean: 21.25 cells/mm^2^, SD: 9.85, *n* = 10) (**Figures 3B, D** **)**. However, in the melanoma tissues, we also observed a few tryptase^−^ C3^+^ cells (3.67 cells/mm^2^, SD: 5.76, *n* = 82). Their number was not significantly different compared with healthy skin control tissue (0.44 cells/mm^2^, SD: 0.32, *n* = 10) (**Figure 3D**). Furthermore, the number of tryptase^−^ C3^+^ cells did not significantly change with melanoma progression, while C3^+^ MAMCs were significantly increased in stage IV (50 cells/mm^2^, SD: 32.2, *n* = 20) in comparison to healthy C3^+^ MCs (21.2 cells/mm^2^, SD: 9.8, *n* = 10). In stage III of melanoma, we observed higher C3^+^ MAMC numbers (35.6 cells/mm^2^, SD: 20.4, *n* = 15). However, this was not significantly different compared with healthy C3^+^ MC numbers (**Figure 3E**). Furthermore, in 50% of tumor biopsies, C3 staining was not detectable in MelA^+^ cells. However, C3 was detected with variable expression among MelA^+^ cells in the same biopsy, with 39% of melanoma tissue displaying less than 1% of MelA^+^ C3^+^ cells and 11% melanoma biopsies with equal or greater than 1% MelA^+^ C3^+^ cells (**Figure 3F**). Moreover, C3 expression was comparable between melanomas at stages III and IV (**Figure 3F**). Thus, our histochemistry data confirm the changes in C3 expression detected by RNA-seq and altogether suggest that MAMCs contribute to C3 expression in the melanoma TME and higher numbers of C3^+^ MCs correlate with disease severity.

Altogether, our findings demonstrate significant transcriptional upregulation of mediators of the complement cascades and a downregulation of degranulation-related pathways in MAMCs. These changes indicate that not only the plasticity of this long-lived tissue-resident immune cell in a tumor context but also the tumor type/microenvironment dictates the MC phenotype.

### Melanoma Cell Secreted Mediators Do Not Induce MC Degranulation But Cytokine Production and TGF-β-Mediated Signaling

To investigate in more detail how melanoma cells modulate the MC functional phenotype and to determine which factors are responsible for phenotype switching, we exposed human MCs generated from blood progenitors (MCs) to supernatants obtained from the culture of two melanoma cell lines: A375 and RPMI-7951. These cell lines are well-established and representatives of the two main phenotypes identified in melanoma. Both A375 and RPMI-7951 cells carrying a BRAF mutation were found at high frequencies in malignant melanoma (24). Nevertheless, while A375 cells are sensitive to BRAF inhibition, RPMI-7951 cells display a BRAF inhibitor-resistant phenotype characterized by a mesenchymal transcriptional signature, which is also linked to resistance to anti-PD1 (25–27).

Cell viability, cell death, and proliferation were investigated in melanoma cell lines co-cultured with MCs. While the co-culture with MCs did not affect RPMI-7951 cell proliferation, cell death, or viability, A375 cells displayed a mild but not significant increase in cell proliferation (**Supplementary Figures 5A–C**). Thus, MCs seem not to influence significantly melanoma cell numbers.

**Figure 4:**
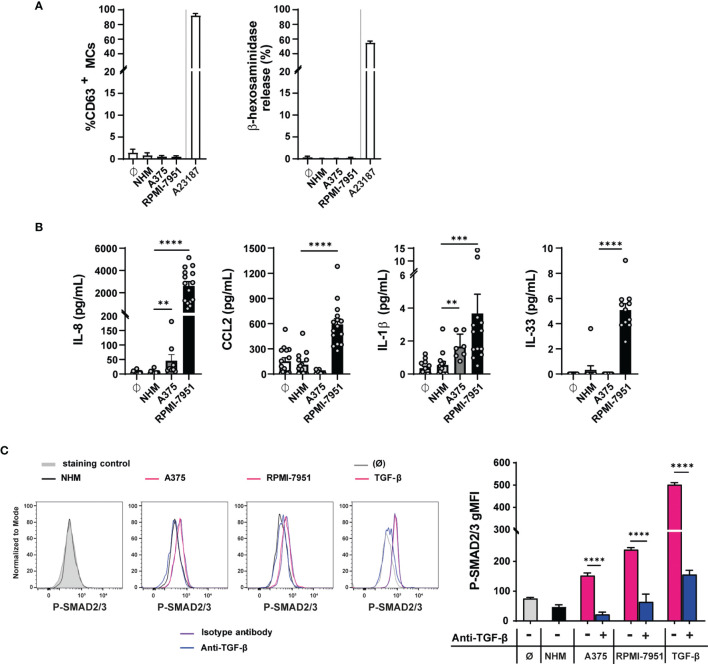
Melanoma cell-secreted mediators do not induce MC degranulation but cytokine production and TGF-β-mediated signaling. Blood-derived human mast cells were incubated 50% v/v with human melanocytes (NHM), A375 (A375), and RPMI-7951 (RPMI-7951) cell culture supernatants or with medium (Ø). **(A)** After 1 h, MC degranulation was assessed by CD63 surface staining, measured by flow cytometry or β-hexosaminidase release assay. Calcium ionophore A23187 was used as positive control. Data shown as mean ± SEM of four independent experiments. **(B)** After 48 h, cytokine levels were quantified in MC pellets by cytometric bead array (CBA) for IL-8, CCL2, and IL-1β and by ELISA for IL-33. Data shown are the average ± SEM of at least four independent experiments (*n* = 7–15). **p < 0.01, ***p < 0.001 using the one-way ANOVA statistical test. **(C)** After 90 min, SMAD2/3 phosphorylation was measured by intracellular flow cytometry staining: as positive control, MCs were treated with TGF-β1 (5 ng/ml), and for negative control, MCs were left untreated (Ø). In **(C)**, the dotted gray histogram shows the isotype control staining and the black line represents MCs treated with NHM culture supernatant cells. The pink lines show the A375 culture supernatant, RPMI-7951 culture supernatant, or TGF-β treatment. In addition, MCs were incubated with the TGF-β blocking antibody, anti-human TGF-β1, 4D11 (100 µg/ml) (anti-TGF-β, blue line), or the isotype blocking antibody (isotype antibody, purple line). On the right, the graph shows the phosphoSmad2/3 geometric mean fluorescence intensity (gMFI) in the different conditions. The data are representative of three independent experiments. Each bar indicates mean ± SEM from three samples (****p < 0.0001 using the two-way ANOVA and Sidak’s multiple comparisons test, comparing the TGFβ blocking-treated sample to its matched untreated control).

To mimic the effect of the TME on degranulation and cytokine secretion, MCs were incubated with melanoma cell-derived conditioned medium for 1 h (**Figure 4A**) or for 3, 6, 12, 24, and 48 h (**Supplementary Figure 6A**). The supernatants derived from the culture of human melanocytes (NHM) were used as control. Melanoma RPMI-7951 and A375 cell culture supernatants did not induce a significant MC degranulation at any time point upon incubation measured by CD63 expression and β-hexosaminidase release, while MC degranulation was observed in MC treated with the calcium ionophore A23187 used as positive control (**Figure 4A** and **Supplementary Figure 6A**). Thus, these findings indicate that melanoma cells do not release mediators that induce MC degranulation.

Next, we investigated whether the melanoma cell supernatant treatment affects the production of selected cytokine and/or chemokines in MCs. CCL2, IL-8, IL-1β, IL-10, IL-33, IL-4, and IL-13 were selected because all are mediators known to play a role in the TME. Compared with controls (untreated, NHM cell culture supernatants), the RPMI-7951 cell culture supernatants upregulated IL-8 (2,604 pg/ml, SD: 1498), CCL2 (591 pg/ml, SD: 258), IL-1β (3.6 pg/ml, SD: 4.3), and IL-33 (5 pg/ml, SD: 1.6) protein amounts in MCs, as shown by flow cytometry analysis of cell pellets, while no IL-10, IL-13, or IL-4 was detectable (**Figure 4B**). The A375 cell culture supernatants induce a slight increase in IL-8 and IL-1β protein but did not upregulate CCL2 and IL-33 (**Figure 4B**).

Furthermore, as shown by the significantly induced levels of phospho-Smad2/3 geometric mean fluorescence (gMFI), both A375 and RPMI-7951 cell culture supernatants engaged SMAD2/3 signaling in MCs, compared with NHM cell culture supernatants or untreated cells. Anti-TGF-β antibodies specifically blocked the effect observed by the A375 (from 152 to 62 gMFI) and RPMI-7951 (from 239 to 64 gMFI) conditioned medium (**Figure 4C**), thus suggesting that TGF-β is released by the A375 and RPMI-7951 melanoma cell lines and affects MC activities. However, blockade of TGF-β activity did not affect significantly IL-8 and CCL2 levels nor IL-1β and IL-33 cytokine production in MC treated with RPMI-7951 supernatant cells, suggesting that TGF-β alone does not modulate the expression of these mediators in MCs (**Supplementary Figure 6B**).

### Mast Cell Treated With Supernatant of Cultured Melanoma Cells Partially Share Their Novel Phenotype With MAMCs

**Figure 5:**
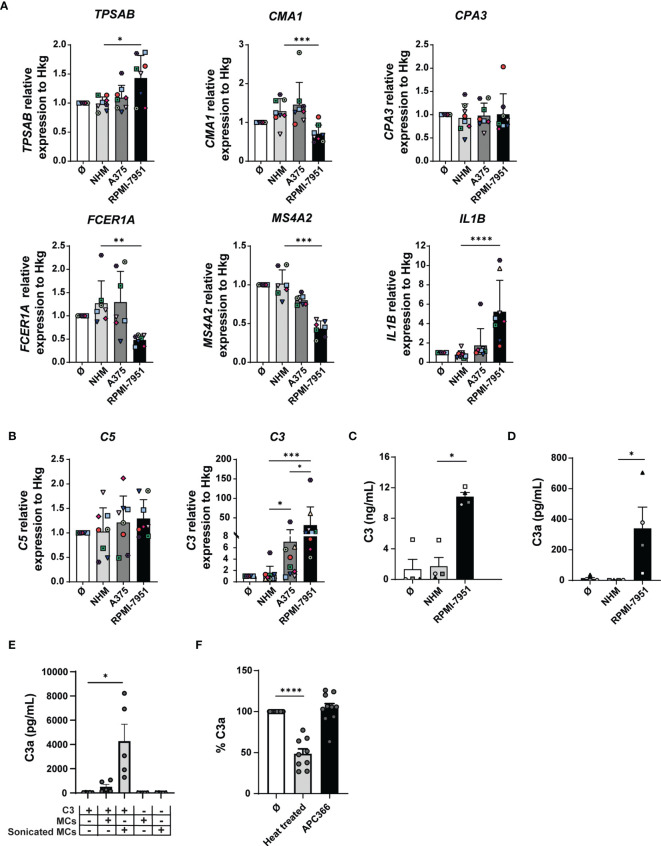
Melanoma cell culture supernatants reproduce some of the transcriptional changes found in MAMCs and induce C3 expression in MCs. MCs were incubated for 48 h with melanoma cell culture supernatants (A375 and RPMI-7951), melanocyte cell culture supernatant (NHM), or medium alone (Ø). **(A)** mRNA levels of tryptase (*TPSAB*), chymase (*CMA1*), carboxypeptidase A3 (*CPA3*), FcεRIα (*FCER1A*), MS4A2 (*MS4A2*), and IL-1β (*IL1B*) and **(B)** mean relative transcription levels of *C3* and *C5* were measured by real-time RT-PCR and normalized by the mean of three housekeeping genes (*PPIA*, *18S*, and *H3-3B*). Data were normalized to medium-treated control samples (Ø). Data represent the mean ± SEM of at least seven independent experiments (*n* = 7–8). **(C)** Quantification of C3 in MC pellets was performed by ELISA. **(D)** C3a was measured by CBA. Results are shown as mean ± SEM of at least four independent experiments. One-way ANOVA statistical test was used: *p < 0.05, **p < 0.01, ***p < 0.001, ****p < 0.0001. **(E)** C3 was incubated for 20 min with MCs in which the cellular membrane was disrupted by sonication (sonicated MCs) or with untreated (membrane-intact) cells (MCs). Concentration of generated C3a was then measured by CBA. Results are shown as mean ± SEM of at least five independent experiments. One-way ANOVA statistical test was used; *p < 0.05, *n* = 5. In **(F)**, C3 was incubated with sonicated MCs used here as control (Ø), heat-treated MCs (2 h at 56°C) (*n* = 9). To inhibit MC tryptase activity, 100 μM of the tryptase-specific inhibitor APC366 was added 30 min before the assay (APC366) (*n* = 10). Data are represented as the mean percentage of C3a in the sonicated MCs control (Ø) ± SEM of at least nine independent experiments. One-way ANOVA statistical test was used; ****p < 0.0001.

Next, we investigated the effect of melanoma cell culture supernatants on the expression of proteases and FcεRI in MCs by qRT-PCR. Compared with medium or NHM, RPMI-7951 cell culture supernatants increased significantly *TPSAB* gene transcription levels (NHM-treated MCs: 0.99, RPMI-7951-treated MCs: 1.43, fold increase: 1.44, *p*-value: 0.03) (**Figure 5A**). In addition, RPMI-7951 medium conditioning decreased significantly the chymase (*CMA1*, NHM-treated MCs: 1.29, RPMI-7951-treated MCs: 0.71, fold decrease: 1.82, *p*-value: 0.0004) and the FcεRIα (*FCER1A*, NHM-treated MCs: 1.27, RPMI-7951-treated MCs: 0.48, fold decrease: 2.65, *p*-value: 0.0024) and β (*MS4A2*, NHM-treated MCs: 1, RPMI-7951-treated MCs: 0.43, fold decrease: 2.3, *p*-value: 0.0005) subunit gene expression in MCs (**Figure 5A**), while the carboxypeptidase A3 (*CPA3*) transcript levels remained unchanged (**Figure 5A**). However, the A375 cell culture supernatants had no significant effect on the gene expression mentioned above (**Figure 5A**). Furthermore, in MCs, *IL1B* transcripts were upregulated by A375 (NHM-treated MCs: 0.80, A375-treated MCs: 1.74, fold increase: 2.1, *p*-value: 0.013) and to a higher extent by the RPMI-7951 (RPMI-7951-treated MCs: 5.22, fold increase: 6.5, *p*-value: <0.0001) cell culture supernatants (**Figure 5A**). This suggests that cell culture supernatants from the RPMI-7951 melanoma cells were found to best reproduce aspects of the MAMC signature. However, we observed some discrepancies between *in-vitro* and *in-vivo* data. While RPMI-7951 melanoma cell supernatant reduced 2.65 times FCER1A expression in MCs *in vitro* and *in vivo*, in MAMCs, the expression of FCER1A was reduced by 16 times.

### Melanoma Cell Culture Supernatants Induce C3 and C3a in Human Mast Cells

Complement activation plays an important role in the regulation of cancer immunity (23). C3, the key complement component, is strongly expressed in all cancer types together with the components of the classical pathway (13). The upregulation of the complement cascade is also one of the key aspects of the MAMC signature (**Figure 2**). Therefore, we investigated the expression of complement components C1R, C1S, C5, and C3 by qRT-PCR in MCs treated with melanoma cell-derived conditioned medium. While we observed no substantial differences in the expression of complement *C1R*, *C1S*, and *C5* (**Figure 5B** and **Supplementary Figure 7B**), *C3* transcripts were significantly increased in MCs treated with RPMI-7951 cell culture supernatants by 24.5-fold (NHM-treated MCs: 1.27, RPMI-7951-treated MCs: 31.3, *p*-value: <0.0001) and with A375 cell culture supernatants by 5.5-fold (A375-treated MCs: 7.05, *p*-value: 0.034) compared with MCs treated with NHM cell culture supernatants (**Figure 5A**).

To validate our qRT-PCR data, we measured C3 protein expression in MCs by ELISA. Since the supernatants of RPMI-7951 but not A375 cells contain C3 (**Supplementary Figures 7C, D** **)** and to investigate the ability of MCs to produce C3 components, C3 detection assays made use of MC pellets and not supernatants. Complement C3 was significantly increased in MCs treated with RPMI-7951 cell culture supernatants (10 ng/ml, SD: 1) compared with NHM controls (**Figure 5C**). Since C3 is cleaved to generate the small bioactive fragments C3a, with both pro- and anti-inflammatory properties (28), we also measured C3a in pellets by CBA. As shown in **Figure 5D**, we found a significant increase in C3a levels (340 pg/ml, SD: 278) in pellets of MCs conditioned with RPMI-7951 cell culture supernatants, the strongest *C3* transcript inducers, compared with NHM control or untreated. Interestingly, the C3aR is constitutively highly expressed on MCs (99.9% of MCs are C3aR positive) and downregulated to 65% (SD: 10, *n* = 5) when MCs are treated for 48 h with RPMI-7951 cell supernatant that contains C3 but not with supernatant derived from NHM melanocytes, suggesting a ligand-induced receptor internalization (**Supplementary Figure 8C**).

To investigate the origin of C3a whether generated upon C3 cleavage at the membrane or intracellularly, C3 (from human serum) was incubated for 20 min with MCs in which the cellular membrane was disrupted by sonication or MCs left untreated (membrane-intact). C3a was then quantified by CBA. While untreated MCs had little effect on C3 cleavage (mean C3a levels: 509 pg/ml, SD: 448, *n* = 5), sonicated MCs generated significant levels of C3a (mean C3a levels: 4,283 pg/ml, SD: 3,105, *n* = 5) compared with the C3 only negative control (mean C3a levels: 37 pg/ml, SD: 13, *n* = 5) (**Figure 5E**). Thus, these findings suggest that for an efficient and biological relevant generation of C3a, the MC intracellular content/compartment is required. While the use of the tryptase inhibitor APC366 did not inhibit C3 cleavage (104% of C3 cleavage activity, SD: 18, *n* = 10), the MC ability to generate C3a was reduced to 48% upon heat treatment (SD: 18, *n* = 9) (**Figure 5F**). These results suggest that C3a generation, in our *in-vitro* system, is a process that is tryptase independent but controlled by MCs.

In summary, C3 seems to be a key complement component highly expressed in the TME, and C3a, the C3-derived anaphylatoxin (C3a), is induced in MCs by melanoma-conditioned medium.

### TGF-β, IL-33, and IL-1β Induce Some of the Changes Found in MAMCs and Significantly Modulate C3 Expression and Activity in Mast Cells

TGF-β, IL-33, and IL-1β exert diverse and complementary effects on MCs (29–31). Furthermore, TGF-β is present in high amounts in the TME (32) together with IL-1β and IL-33 (9, 11). IL-33 was not detectable in melanoma cell culture supernatants. However, the cell culture supernatants from A375 and RPMI-7951 melanoma cell lines contain TGF-β and IL-1β (**Supplementary Figures 7A, B** **)** and also profoundly increase the production of IL-1β and IL-33 in MCs. We asked, therefore, whether these cytokines could contribute to the melanoma TME-induced phenotype switching in skin MCs. To this purpose, we have tested the effect of the pro-tumoral cytokines TGF-β, IL-1β, and IL-33 on the expression of proteases, FcεRIα, and complement components in MCs.

**Figure 6:**
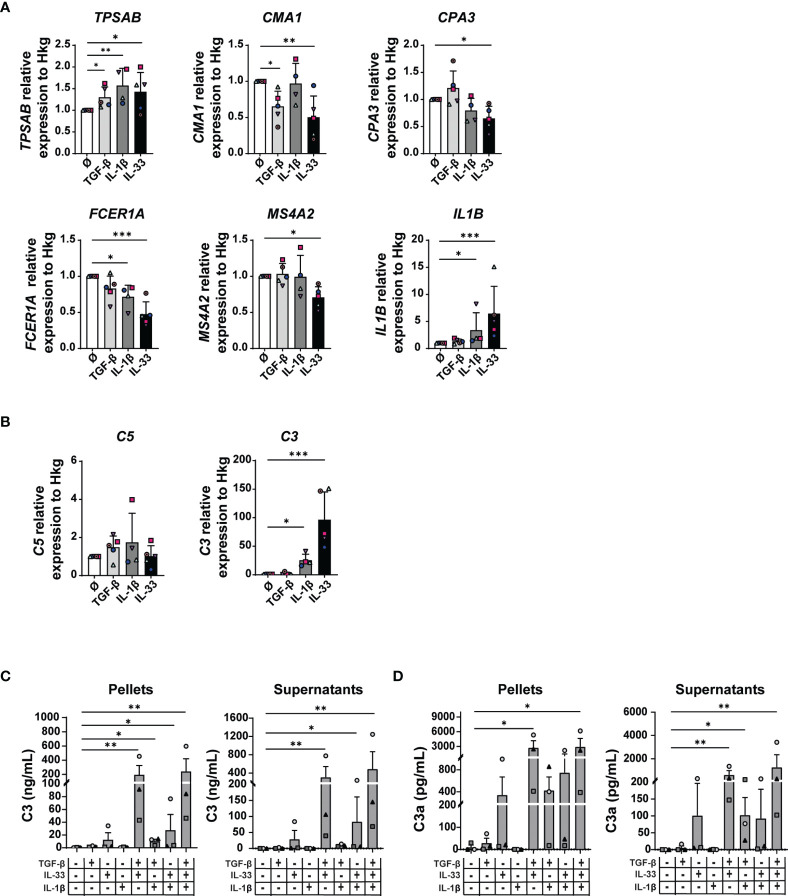
TGF-β, IL-33, and IL-1β induce some of the changes found in MAMCs. MCs were treated 48 h with 50 ng/ml of TGF-β1, IL-1β, and IL-33 cytokines alone or in different combinations as indicated. Tryptase (*TPSAB*), chymase (*CMA1*), carboxypeptidase A3 (*CPA3*), FcεRIα (*FCER1A*), MS4A2 (*MS4A2*), and IL-1β (*IL1B*) **(A)** and C3 and C5 **(B)** mRNA levels were measured by RT-PCR and normalized by the mean of three housekeeping genes (*PPIA*, *18S*, and *H3-3B*). Data were normalized to medium-treated control samples (Ø). Data represent the mean ± SEM of four independent experiments (*n* = 4–5) in **(A, B)**. Quantification of C3 protein in MC pellets and supernatants was performed by ELISA **(C)**. C3a was measured using CBA **(D)**. Results are shown as mean ± SEM of three independent experiments (*n* = 3) in **(C, D)**. One-way ANOVA statistical test was used; *p < 0.05, **p < 0.01, ***p < 0.001.

TGF-β, IL-1β, and IL-33 significantly increased tryptase (*TPSAB*) gene transcription levels (**Figure 6A**). Furthermore, chymase expression was reduced in the presence of TGF-β or IL-33 but not upon IL-1β stimulation. While IL-1β and IL-33 were able to inhibit *FCER1A* expression (untreated MCs: 1, IL-1β-treated MCs: 0.71, fold decrease: 1.4, *p*-value: 0.038 and IL-33: 0.47, fold decrease: 2.1, *p*-value: 0.0004), only IL-33 reduced *MS4A2* transcripts in MCs (untreated MCs: 1, IL-33-treated MCs: 0.70, fold decrease: 1.4, *p*-value: 0.01) (**Figure 6B**). The carboxypeptidase A3 (*CPA3*) expression was not challenged by cytokine treatment with the exception of IL-33 that inhibited its expression (**Figure 6B**). In MCs, *IL1B* transcripts were upregulated by the IL-1β cytokine itself and to a higher extent by IL-33 (**Figure 6A**).

We observed no substantial differences in the expression of complement *C1R*, *C1S*, and *C5* (**Figures 6A, B** and **Supplementary Figure 8B**). While TGF-β stimulation had no effect on C3 mRNA levels, both IL-1β (25-fold increase, *p*-value: 0.04) and IL-33 (96-fold increase, *p*-value: 0.0007) were potent inducers of C3 (**Figure 6B**).

Interestingly, although C3 mRNA levels in MCs were increased by individual stimulation with IL-1β and IL-33, C3 protein levels were significantly detectable in MC pellets and supernatants only when cytokines were combined: TGF-β+IL-33, 196 ng/ml (SD: 224) in cell pellets and 307 ng/ml (SD: 407) in cell supernatants; TGF-β+IL-33+IL-1β, 242 ng/ml (SD: 307) in cell pellets and 487 ng/ml (SD: 659) in cell supernatants (**Figure 6C**). C3a protein levels were greater in MC supernatants upon stimulation with multiple cytokines: TGF-β+IL-33, 2,746 pg/ml (SD: 2476) in MC pellets and 608 pg/ml (SD: 647) in MC supernatants; TGF-β+IL-33+IL-1β, 2,923 pg/ml (SD: 2,953) in MC pellets and 1,265 pg/ml (SD: 1,863) in MC supernatants (**Figure 6D**). Interestingly, in TGF-β-, IL-1β-, and IL-33-treated MC pellets, we observed both C3 and cleaved C3a forms, suggesting that C3 can be cleaved in C3a intracellularly (**Figures 6C, D** **)**.

Our findings suggest that TGF-β, IL-33, and IL-1β exert diverse and complementary effects on MC transcriptional activities. They mimic some of the properties of the melanoma cell-conditioned medium and the MAMC signature. Therefore, these cytokines could contribute to the melanoma TME-induced MC phenotype switch.

### TGF-β, IL-33, and IL-1β in Melanoma-Conditioned Media Act in Concert to Induce C3 and C3a in Human Mast Cells

**Figure 7:**
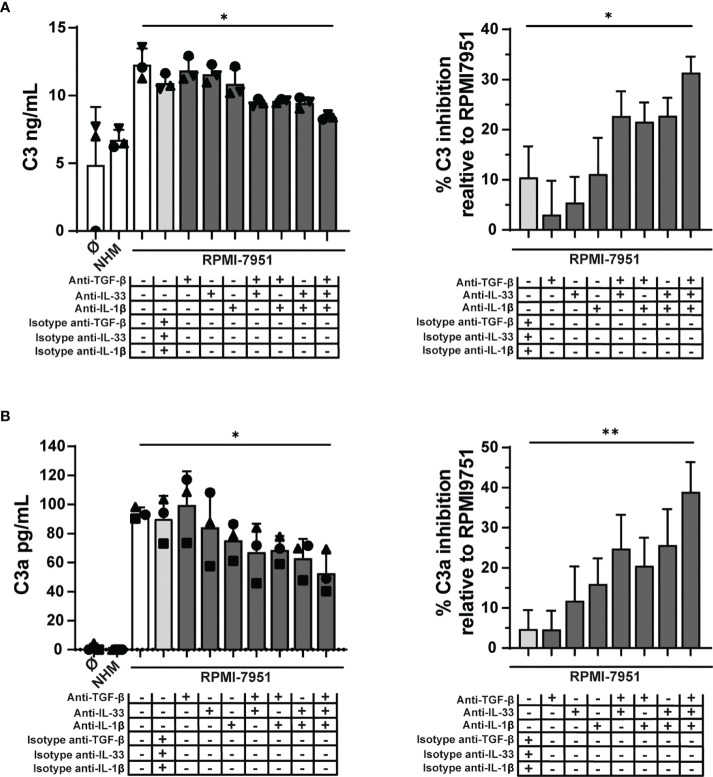
Melanoma cell mediators regulate C3 in MCs. MCs were cultured for 48 h with RPMI-7951 melanoma cell culture supernatant, melanocyte cell culture supernatant (NHM), or with medium alone (Ø). Before treatment, MCs were incubated 30 min with different combinations of blocking antibodies, anti-human IL-33 (50 μg/ml), anti-human IL-1β (50 μg/ml), anti-human TGF-β1 (100 μg/ml), or appropriate isotype control antibody. In MC pellets, C3 **(A)** and C3a **(B)** were measured, respectively, by ELISA and CBA. Graphs on the right represent percent inhibition of C3 and C3a expression relative to protein levels in MC treated with RPMI-7951 melanoma cell culture supernatant alone. Data are shown as mean ± SEM of three independent experiments (*n* = 3). One-way ANOVA statistical test was used; *p < 0.05, **p < 0.01.

To investigate whether the C3 modulatory effects observed in MCs treated with RPMI-7951 melanoma-conditioned media could be attributed to the TME cytokines TGF-β, IL-1β, and IL-33 (expression of the latter induced in MCs by melanoma cell supernatants), we blocked their activities using neutralizing antibodies or isotype controls. Individual cytokine blockade had no effect, and a minimal but non-significant effect was observed by combining two cytokines. However, C3 expression and C3a generation were significantly inhibited, 38% (SD: 14) and 31% (SD: 5), respectively, when all three cytokine activities were blocked (**Figures 7A, B** **)**. Thus, these findings suggest that TGF-β, IL-33, and IL-1β act in concert to induce C3 and C3a in MCs.

### MCs Expressing C3 Are Linked to Low Survival Rates in Melanoma Patients

**Figure 8:**
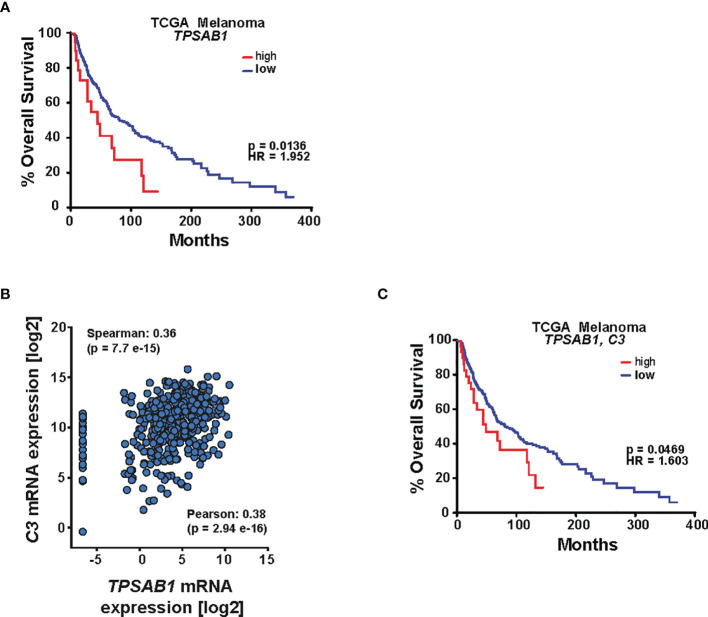
MCs expressing C3 are linked to low survival rates in melanoma patients. **(A)** Kaplan–Meier analysis of The Cancer Genome Atlas (TCGA) melanoma patient cohort. Differences in overall survival for patients whose tumors express high or low levels of *TPSAB1* are shown. The data were analyzed for mRNA expression (*z*-score 2.0) in cBioPortal, and survival data were extracted and analyzed in GraphPad Prism. Log-rank (Mantel–Cox) test was used; *p* = 0.0136 and hazard ratio (HR) = 1.952. **(B)** Correlation analysis of the TCGA melanoma patient cohort for *C3* and *TPSAB1*. The TCGA dataset was interrogated in cBioPortal and the data were extracted and further analyzed in GraphPad Prism. Spearman as well as Pearson correlation is indicated. **(C)** Kaplan–Meier analysis of the TCGA melanoma patient cohort. Differences in overall survival for patients whose tumors express high or low levels of *C3* and *TPSAB1* were analyzed. cBioPortal assessing patient survival for *C3* and *TPSAB1* mRNA expression (*z*-score 2.1). Data were extracted and further analyzed in GraphPad Prism. Log-rank (Mantel–Cox) test was used; *p* and hazard ratio (HR) values are indicated.

We next interrogated the TCGA patient data and analyzed the survival for patients whose tumors express high or low mRNA levels of the tryptase gene *TPSAB1*, and we observed that patients with a higher level of *TPSAB1* mRNA exhibited a significantly worse survival rate [log-rank (Mantel–Cox) *p* < 0.05], suggesting the unfavorable role of MCs in melanoma patient survival (**Figure 8A**). To investigate whether C3^+^ MCs associate with melanoma prognosis, we first assessed the correlation of *C3* expression with MC-specific *TPSAB1*. Interrogating the TCGA dataset revealed a highly significant correlation between *TPSAB1* with *C3* (**Figure 8B**). This confirms the relevance of MCs for *C3* expression in melanoma and is entirely in line with previous data from scRNA-seq demonstrating that *C3* expression in the melanoma TME comes from the immune cell population (33). Most importantly, a higher expression of *TPSAB1* and *C3* significantly correlated with poorer patient survival [log-rank (Mantel–Cox) *p* < 0.05] (**Figure 8C**), thus supporting the prognostic impact of C3^+^ MCs in melanoma.

## Discussion

In this study, to investigate the role of MCs in modulating the melanoma TME, we have characterized the transcriptional signature of MCs from melanoma lesions and studied how this differs functionally from MCs residing in BCC and healthy skin. Our findings demonstrate the plasticity of these long-lived tissue-resident immune cells in a tumor context and elucidate the novel mechanisms used by MCs in shaping the melanoma TME.

Recruitment of MCs to the TME has been described previously (34, 35). We show that the number of MCs is increased in the proximity of both malignant melanoma and benign BCC. One factor that effectively induces the migration/chemotactic activity of MCs is the stem cell factor (SCF) (36). Noteworthy, the expression of SCF is significantly increased in BCC and melanoma (37), suggesting an involvement of this factor in the increased MC numbers we observed. MCs comprise in melanoma tissue, MAMCs, and in BCC tissue, BCCMCs are accompanied by changes in phenotypes. Both populations display a novel transcriptional signature as demonstrated by RNA-seq analysis. While BCCMCs displayed an upregulation of the FcεRI-signaling pathway genes, MAMCs were significantly and uniquely challenged in their phenotype. In MAMCs, we found that, together with angiogenic factors, metalloproteases MMP14 and MMP17 were significantly upregulated. This agrees with published evidence (38) that reports the increase of MMP expression in melanoma and their involvement in tissue disruption and tumor growth.

Furthermore, MAMCs downregulate the expression of *FCER1A* and *MRGPRX2*, both receptors involved in inducing MC degranulation, which have been shown to decrease upon chronic IL-33 exposure (31, 39). Interestingly, MAMCs upregulate complement C3. To investigate TME mediators implicated in the modulation of C3 in MAMCs, we have used the supernatants of cultured melanoma cell lines RPMI-7951 and A375 to condition blood-derived MCs. We observed some discrepancies between the RNA-seq data obtained for MAMCs and the direct analysis of MCs exposed to soluble factors contained in melanoma cell culture supernatants or cytokines. One of the possible explanations is that *in-vivo* MCs are influenced by other factors than the ones released by tumor cells. Indeed, MAMCs are exposed to a wide variety of residents and recruited immunocytes as well as skin structural mediators and cells.

Similar to the skin MC_TC_ (MC tryptase^+^ chymase^+^), our MCs generated *in vitro* express both tryptase and chymase in their secretory granules (15, 40). Treated MCs produced C3 protein and cleaved it intracellularly into C3a. This process occurs in skin MCs where β-tryptase has been described to generate anaphylatoxin C3a from C3 in a convertase- and lectin-independent manner (41). However, in our *in-vitro* studies, we showed that intracellular MC mediators cleave C3 in a tryptase-independent manner. This is in line with the findings of Elvington et al. that demonstrated that the C3 proteolytic activity *in vitro* is controlled by multiple intracellularly stored MC proteases such as cathepsin L, elastase, and granzyme B (42). Interestingly, only by neutralizing TGF-β, IL-33, and IL-1β cytokines simultaneously, we found that the upregulation of complement C3 and C3a expression in conditioned MCs was inhibited, suggesting a synergistic activity of those cytokines in regulating C3 expression and activity in MCs.

TGF-β, IL-33, and IL-1β are present in the TME. While we provided evidence that melanoma cells can produce both TGF-β and IL-1β, IL-33 was undetectable in supernatants from both A375 and RPMI-5971 cells. However, we observed that MCs produced IL-33 and IL-1β and acted as autocrine inducers of C3 expression. Furthermore, Fukuoka et al. showed *in vitro* that other melanoma TME pro-inflammatory cytokines like TNF-α act in synergy with IL-4 or IL-13 to induce C3 expression in human MCs (43). Thus, the fact that multiple cytokines synergize in modulating C3 expression would explain why TGF-β, IL-33, and IL-1β activity blockade did not fully inhibit the expression of C3 in MCs treated with melanoma cell culture supernatants.

In our study, we have demonstrated in the tissue and in a cell culture system that both melanoma cells and MCs produce C3. *In-vitro* MCs exposed to melanoma cell supernatants upregulate C3 expression. MCs treated with TGF-β, IL-1β, and IL-33 showed an increased expression of C3 and C3a both intracellularly and in cell culture supernatants. The intracellularly stored MC proteases seem to be most efficient in cleaving the newly synthesized C3 into C3a. Altogether, the data support the concept that C3 is a key mediator in melanoma, but importantly, MCs in the presence of tumor cell-secreted mediators, such as cytokines, have the ability to synthesize C3 and cleave it to C3a.

In cancer, the complement system was found to exert a critical anti-tumor function by inducing cell cytotoxicity either directly (complement-dependent cytotoxicity, CDC) or indirectly through antibodies (antibody-dependent cell-mediated cytotoxicity, ADCC), the latter being relevant in antibody-based immunotherapy (44, 45). However, recent studies have demonstrated the expression of complement components C3 and C5 and the anaphylatoxins C3a and C5a in several types of tumors (12, 14, 46) and their role in promoting tumor growth (46, 47), angiogenesis, metastasis (48, 49), immunosuppression, inhibition of T-cell responses (14, 50), and regulatory cell recruitment [myeloid-derived suppressor cells (MDSCs) and tumor-associated macrophages (TAMs)] (51, 52). Moreover, C3 expression correlated in resistance to PD-L1 antibody treatment in a mouse model of B16F10 and CT26 tumors (52). Altogether, these data indicate a role for the complement system, in particular for C3 and C3a, in making the TME beneficial to tumor growth, proliferation, and angiogenesis.

In addition, we found that conditioned MCs could be a source of the pro-tumorigenic cytokine IL-8 (53); CCL2, a potent chemokine that recruits MDSCs and TAMs (54, 55); IL-33 that modulates melanoma cell proliferation, angiogenesis, and regulatory T cells (11, 56, 57); and the pro-angiogenic cytokine IL-1β (58, 59). Melanoma cells apart from TGF-β produce many cytokines, among them growth factors such as TGFA, EGF, and HGF as well as cytokines such as IL-6, IL-8, and IL-1β (60–63). All these factors could potentially induce the expression of IL-8, CCL2, and IL-1β in MCs. MCs seem not to influence significantly the proliferation and viability of the melanoma cell lines RPMI-7951 and A375. However, we cannot exclude that the impact could be cell line dependent.

In the present study, we provide evidence that the melanoma micromilieu enhances the production of CCL2, IL-8, IL-1β, and C3/C3a by MCs, factors associated with tumor progression. Furthermore, we observed a higher C3^+^ MAMC number in the latest melanoma stage and a significantly worse survival rate in melanoma patients expressing a higher level of combined *TPSAB1* and *C3* mRNA. Our findings are in accordance with previous studies that correlate increased MC density with increased microvascularity and a poor prognosis in human melanoma, therefore suggesting a pro-tumorigenic role for MCs (64, 65). Conversely, evidence is available for an anti-tumorigenic role of MCs in melanoma showing that MC proteases including tryptase, chymase, and carboxypeptidase A3 promote cytotoxic T and NKT cells (66–68). Interestingly, depending on the melanoma stage, MC frequency correlated with poor prognosis in deep but not in superficially invasive melanomas (68). We indeed observed a difference in the intensity of the response of MCs to the malignant metastatic RPMI-7951 versus the primary A375 melanoma cell line. Both cell lines displayed a significant differential gene expression profile that could act differently on MCs (69). Thus, we suggest that the discrepancies in the literature concerning the role and activities displayed by MCs in various tumor types and stages could be explained by the diversity in nature and concentrations of mediators found in the different TMEs (70, 71).

In summary, our findings demonstrate that MAMCs are producers of pro-tumorigenic mediators such as pro-angiogenic factors and specific chemokines/cytokines (CCL2, IL-8, IL-1β, and IL-33). Most importantly, a significant transcriptional downregulation of the FcεRI-signaling pathway and upregulation of mediators of C3 and C3a were detectable in MAMCs. These changes indicate that tumors dictate MC characteristics and activities that define a new melanoma skin MC-associated phenotype. Most importantly, we found a significant correlation between the increase of C3^+^ MC numbers, increase of disease severity, and poor prognosis, suggesting a prognostic impact of C3^+^ MCs in melanoma. Thus, the manipulation of this novel MC phenotype and its mediators could deliver innovative therapeutic strategies.

## Data Availability Statement

The datasets presented in this study can be found in online repositories. The names of the repository/repositories and accession number(s) can be found below: https://www.ncbi.nlm.nih.gov/geo/query/acc.cgi?acc=GSE196292.

## Ethics Statement

The studies involving human participants were reviewed and approved by the UK NHS Human Research Authority, North West Greater Manchester West Research Ethics reference 17/NW/0328; University of Zurich Hospital Biobank Ethics Committee reference EK647 and EK800, BASEC-Nr.PB-2017-00494; and the University of Manchester Research Ethics Committee (UREC ref 2018-2696-5711). The patients/participants provided their written informed consent to participate in this study.

## Author Contributions

RB, OK, KG, HA, and MS performed the experiments. IP performed the RNA-seq analysis. RB, CW, JMMG, RD, ML, and SB-P participated in the research design and interpretation of the data. RB and SB-P contributed to the writing of the manuscript. All authors contributed to the article and approved the submitted version.

## Funding

RB, SB-P, and CW were supported by CRUK (C21043, C11591/A16416). OK was supported a British Skin Foundation research grant. The Kennedy Trust supported IP for rheumatology research. KG was supported by a CONACyT fellowship. HA was supported by a Ph.D. studentship from the Psoriasis Association. CW and SB-P were supported by the University of Manchester.

## Conflict of Interest

RD has intermittent, project-focused consulting and/or advisory relationships with Novartis, Merck Sharp & Dhome (MSD), Bristol-Myers Squibb (BMS), Roche, Amgen, Takeda, Pierre Fabre, Sun Pharma, Sanofi, Catalym, Second Genome, Regeneron, Alligator, MaxiVAX SA, and touchIME outside the submitted work.

The remaining authors declare that the research was conducted in the absence of any commercial or financial relationships that could be construed as a potential conflict of interest.

## Publisher’s Note

All claims expressed in this article are solely those of the authors and do not necessarily represent those of their affiliated organizations, or those of the publisher, the editors and the reviewers. Any product that may be evaluated in this article, or claim that may be made by its manufacturer, is not guaranteed or endorsed by the publisher.

## Abbreviations

BCC, basal cell carcinoma; BCCMC, basal cell carcinoma-associated mast cell; CCL2, C-C motif chemokine ligand 2; HSMC, healthy skin-associated mast cell; IL-1β, interleukin 1 beta; IL-8, interleukin 8; IL-33, interleukin 33; MAMC, melanoma-associated mast cell; MEL, melanoma; MelA, melan-A; MC, mast cell; MDSC, myeloid-derived suppressor cell; MMP, metalloprotease; NHM, normal human melanocyte; SD, standard deviation; TAM, tumor-associated macrophage; TGF-β, tumor growth factor beta; TME, tumor microenvironment.
