# Supplementary material for: Human Melanoma-Associated Mast Cells Display a Distinct Transcriptional Signature Characterized by an Upregulation of the Complement Component 3 That Correlates With Poor Prognosis
Source: Front Immunol. 2022 May 20;13:861545. doi: 10.3389/fimmu.2022.861545 (PMC9163391; doi:10.3389/fimmu.2022.861545)
Supplement: Supplementary file 1 [file DataSheet_1.pdf]

Supplementary Figure 1: Skin mast cell isolation

A. Sample preparation

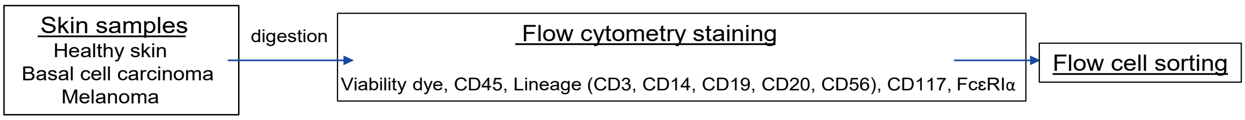

B. Sorting strategy

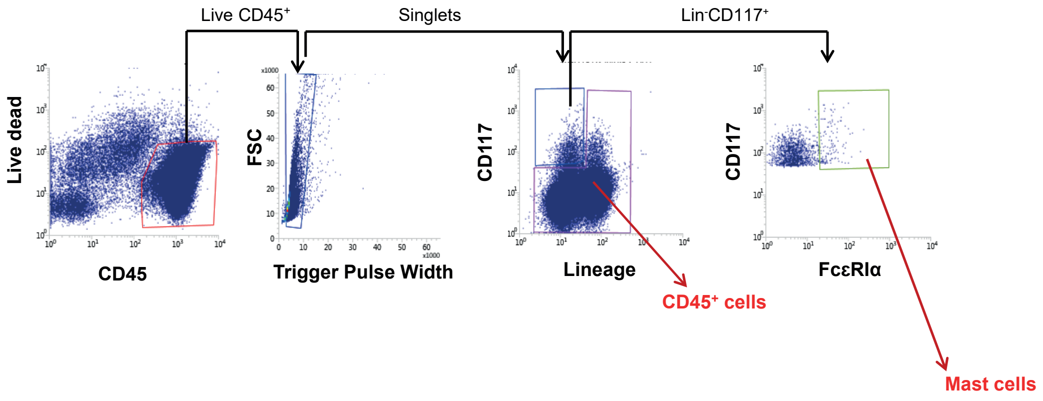

Supplementary Figure 1: Skin mast cell isolation. A. Human skin biopsies were digested for 3-6 hours with collagenase. Cell suspensions were stained with CD45, Lin (CD3, CD14, CD19, CD20, CD56), CD117, FcεRIα antibodies and a viability dye. Cells were sorted by flow cytometry using the FACS Aria cytometer. **B**. The plots show the gating strategy for cell sorting. After exclusion of dead cells and doublets, $CD45^+Lin^-CD117^+$ cells were gated and $CD45^+Lin^-CD117^+FcεRIα^+$ cells were sorted as MCs. The remaining cells, $CD45^+Lin^+CD117^+$, $CD45^+Lin^+CD117^-$ and $CD45^+Lin^-CD117^-$ were sorted as $CD45^+$ cells.

Supplementary Figure 2: Transcriptional analysis of skin CD45^{+}cells

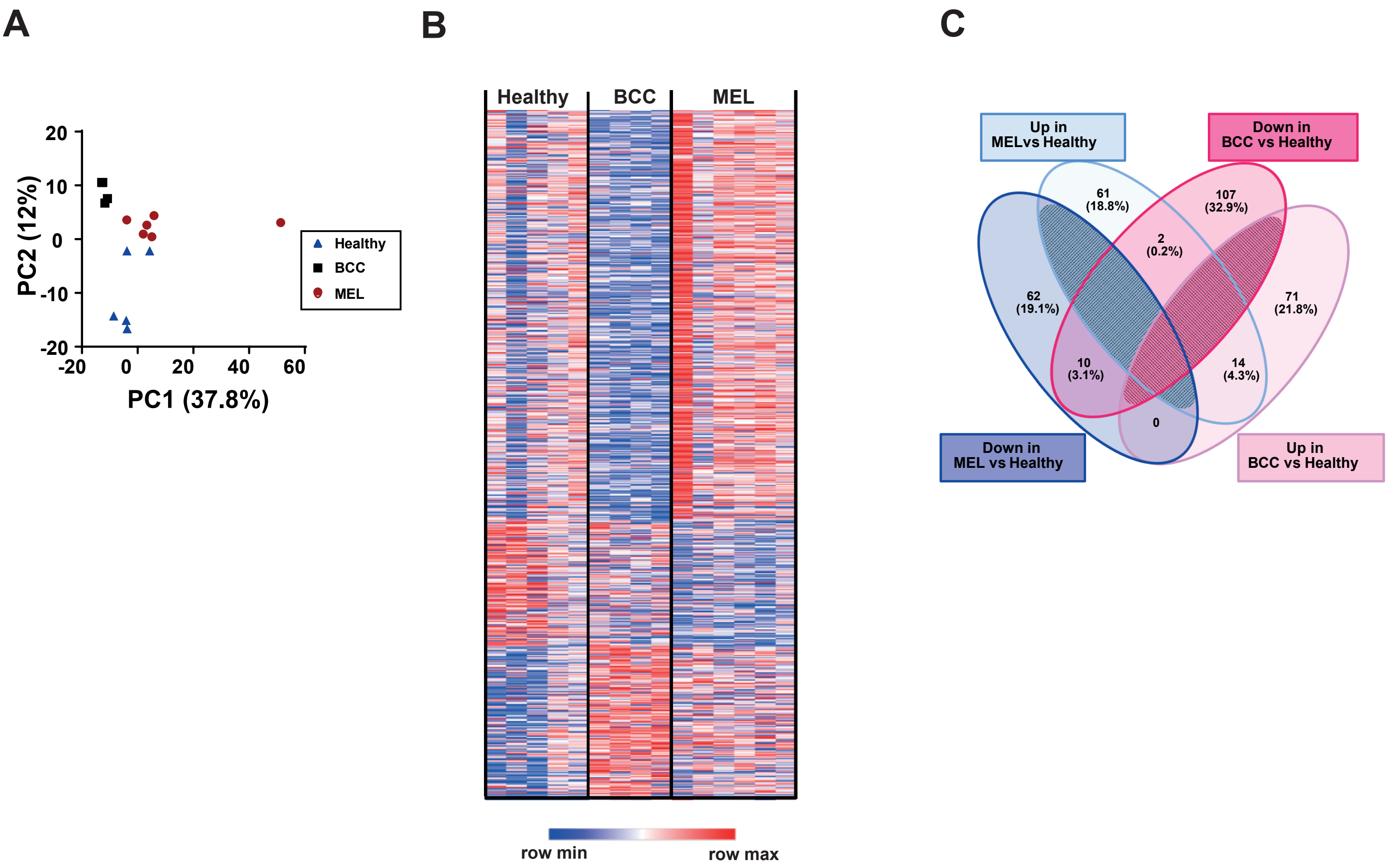

Supplementary Figure 2: Transcriptional analysis of CD45^{+} cells. CD45^{+} cells were sorted from 5 healthy skin, 4 BCC and 3 MEL biopsies. The RNA was extracted and the Illumina RNAseq analysis was performed. **A** principal component analysis (PCA) biplot of gene expression data where each biopsy is represented by a symbol: healthy skin CD45^{+} cells in blue triangles, CD45^{+} cells from BCC in black squares and CD45^{+} cells from MEL in red circles. **B**, heat maps show z-scores of averaged expression values of genes in CD45^{+} cells isolated from healthy skin, BCC and MEL; red to blue scale represents the intensity of fold change per genes (red indicates higher z-score, blue indicates lower z-score). Each row shows an individual gene and each column a sample. **C**, Venn diagrams displays significantly up- and down-regulated transcripts in CD45^{+} cells isolated from BCC (pink), MEL (blue) versus Healthy skin. Numbers represent gene numbers in different groups.

Supplementary Figure 3: MAMCs display a downregulation of FcεRI signalling pathway-related genes while genes associated with the complement cascade are upregulated

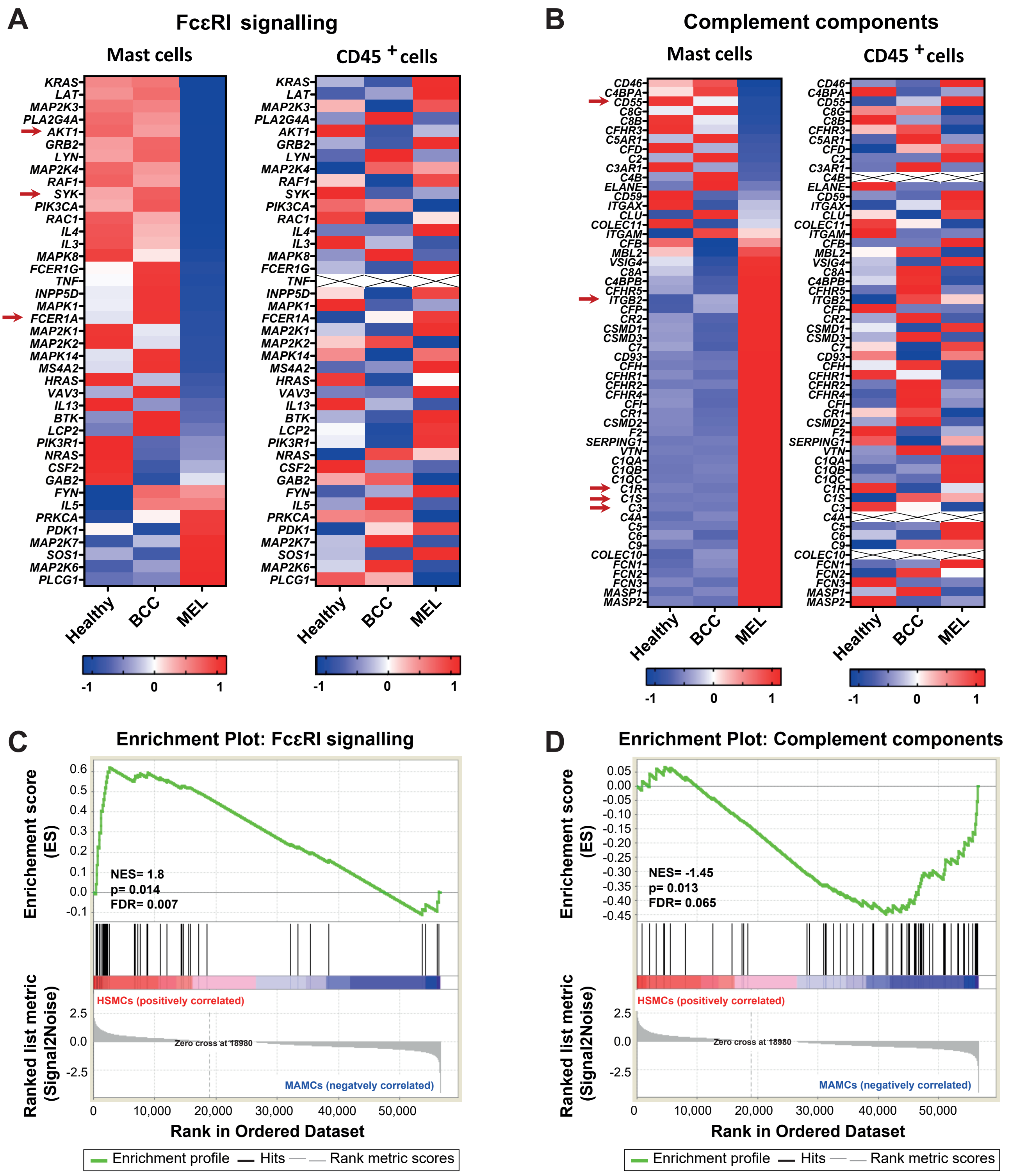

Supplementary Figure 3: MAMCs display a downregulation of FcεRI signalling pathway-related genes while genes associated with the complement cascade are upregulated. Mast cells were isolated by flow cytometry from 8 healthy skin (Healthy), 13 BCC and 4 melanoma (MEL) biopsies. $CD45^{+}$ cells were sorted from 5 healthy, 4 BCC and 3 MEL skin biopsies. The RNA was extracted and the Illumina RNAseq analysis was performed. Heat map showing z-scores of averaged gene expression of FcεRI signalling pathway (**A**) and complement cascade related genes (**B**) in $CD45^{+}$ cells and MCs isolated from healthy, BCC and MEL skin biopsies; red to blue scale represents intensity of gene expression (red indicates higher z-score, blue indicates lower z-score). Red arrows highlight the significant expressed genes in MAMCs compared to healthy skin MCs. Gene Set Enrichment Analysis (GSEA) curves of FcεRI signalling pathway (**C**) and complement components (**D**) related gene sets in healthy skin mast cells (HSMCs) versus melanoma associated mast cells (MAMCs). In GSEA thumbnails, the green curve represents the enrichment score curve. Genes on the far left (red) correlated with HSMCs, and genes on the far right (blue) correlated with MAMCs. The vertical black lines indicate the position of each gene in the studied gene set. The normalized enrichment score (NES), false discovery rate (FDR) and nominal p value (p) are indicated.

Supplementary Figure 4: MAMCs transcriptional signature: angiogenesis factors and proteases

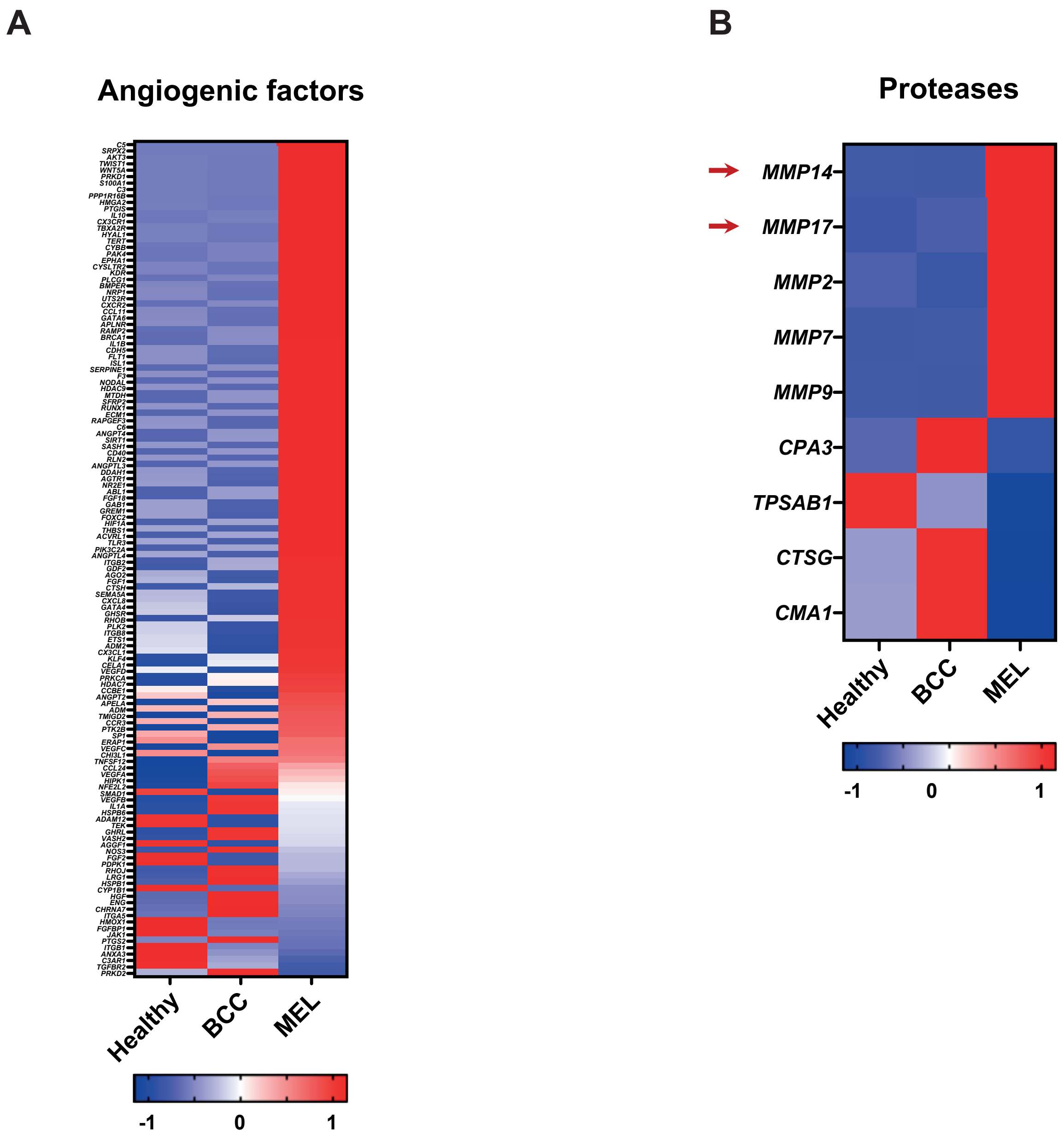

Supplementary Figure 4: MAMCs transcriptional signature: angiogenesis factors and proteases. Mast cells were isolated by flow cytometry from 8 healthy skin (Healthy), 13 BCC and 4 melanoma (MEL) biopsies. The RNA was extracted and the Illumina RNAseq analysis was performed. Heat map showing z-scores of averaged gene expression of angiogenic factors (**A**) and proteases genes (**B**) in MCs isolated from healthy, BCC and MEL skin biopsies; red to blue scale indicates intensity of gene expression (red indicates higher z-score, blue indicates lower z-score). Red arrows highlight the significant expressed genes in MAMCs compared to healthy skin MCs.

Supplementary Figure 5: *In-vitro*, MCs do not influence significantly melanoma cell line proliferation and viability

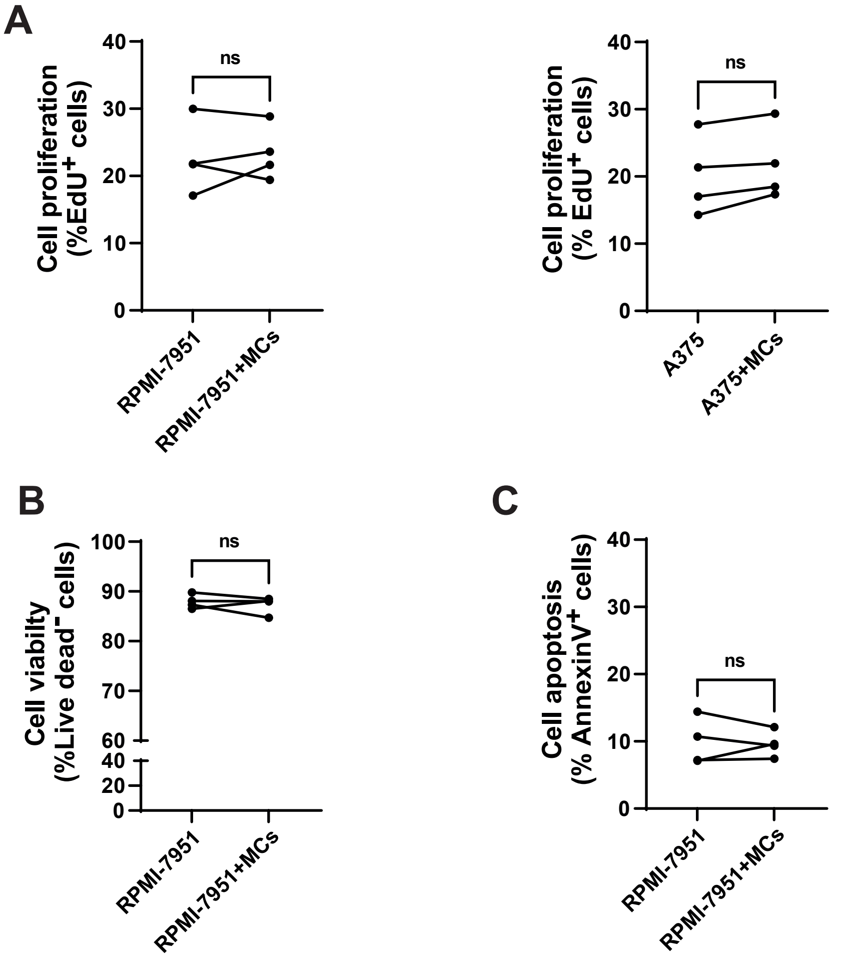

Supplementary Figure 5: *In-vitro,* MCs do not influence significantly melanoma cell line proliferation and viability. (**A**) A375 and RPMI-7951 melanoma cell lines were cultured 48 hours in the presence of MCs (ratio 2:1, melanoma cells:MCs). As negative control melanoma cells were cultured alone (A375 or RPMI-7951). Click-iT™ Plus EdU Alexa Fluor™ 647 Flow Cytometry Assay Kit was used for analysing DNA replication in proliferating cells. 10 µM EdU was added the last 2 hours in cell cultures. Cells were harvested and CD45 surface flow cytometry staining was performed. Cells were fixed and EdU incorporation was measured by intracellular flow cytometry staining. Data were analysed using the Flowjo software (Treestar Inc.) and melanoma cell proliferation was measured as a percentage of EdU^+ cells in gated $CD45^-$ cells to exclude MCs. The data represent 4 independent experiments. Statistical comparisons were performed using Mann-Whitney U-test. ns= not significant.
For viability (**B**) and apoptosis (**C**) measurement, cell Trace Violet proliferation kit labelled MCs were added to RPMI-7951 melanoma cell lines cultures for 24 hours (ratio 2:1, melanoma cells:MCs). As negative control melanoma cells were cultured alone (RPMI-7951). Harvested cells were washed with cold PBS twice and incubated with live/dead™ blue viability dye. After a wash, cells were stained with FITC Annexin V. Then, cells were washed and resuspended in Annexin V Binding Buffer and analysed by flow cytometry (LSRII or LSRFortessa flow instrument, BD Biosciences). Data were analysed using the Flowjo software (Treestar Inc.). Melanoma cells were gated on Cell Trace Violet negative population to exclude MCs and the viability was measured as a percentage of Live/dead blue dye negative cells and the necrotic/apoptotic cells as the percentage of Annexin V^+ cells. The data represent 4 independent experiments. Statistical comparisons were performed using Mann-Whitney U-test. ns= not significant.

Supplementary Figure 6: Melanoma cell culture supernatants do not induce MC degranulation but a TGF-β-independent release of mediators

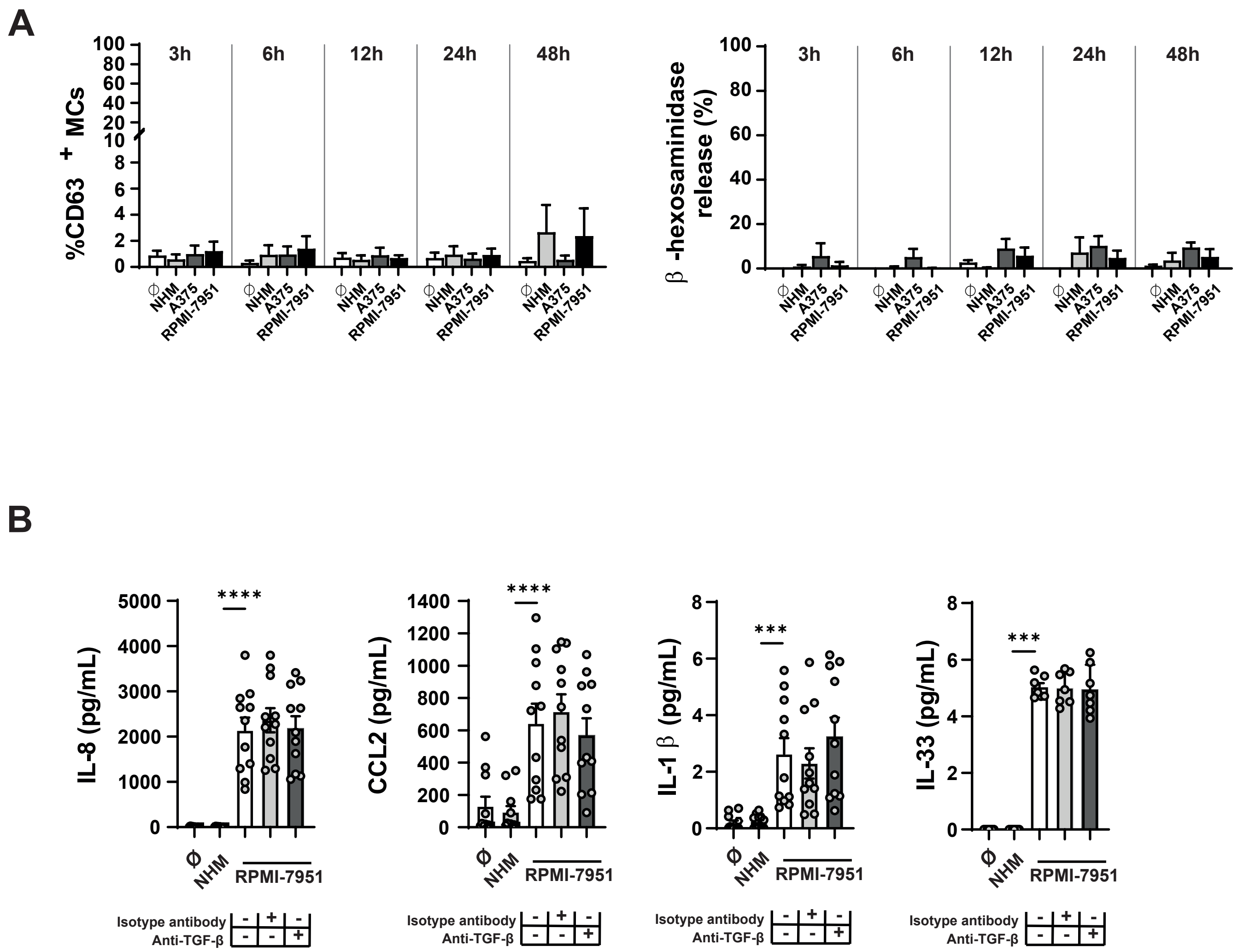

Supplementary Figure 6: Melanoma cell culture supernatants do not induce MC degranulation but TGF-β-independent release mediators. Mast cells were incubated 50% v/v with human melanocytes (NHM), A375 (A375) and RPMI-7951 (RPMI-7951) cell supernatants or with medium (Ø). **A**, MC degranulation was assessed by CD63 surface staining measured by flow cytometry or β-hexosaminidase release assay at 3, 6, 12, 24 and 48 hours. Data shown as mean ± SEM from four experiments (n=4). **B**, at 48 hours, in the presence of anti-human TGF-β1 (100 µg/mL) or isotype control antibody, cytokine levels were quantified in MC pellets by CBA for IL-8, CCL-2, IL1-β and by ELISA for IL-33. Data shown are the average ± SEM of at least four independent experiments (n=7-11). ***p < 0.001, ****p < 0.0001 using the one-way ANOVA statistical test.

Supplementary Figure 7: TGF-β, IL-1β, C3 and C3a concentrations in melanoma cell culture supernatants

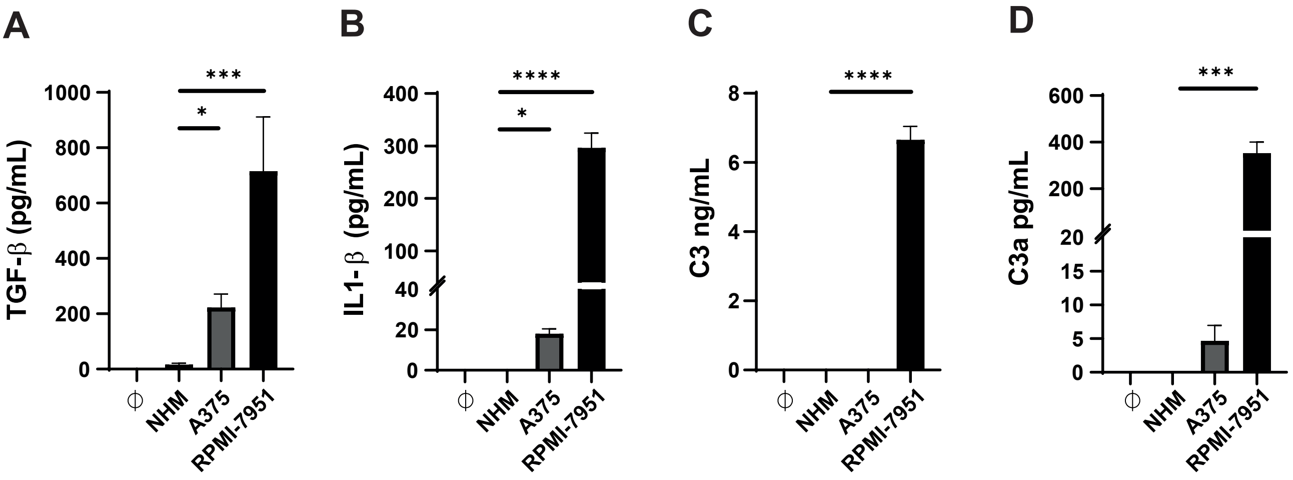

Supplementary Figure 7: TGF-β, IL-1-β, C3 and C3a concentrations in melanoma cell culture supernatants. NHM, A375, RPMI-7951 cells were cultured at confluence for 24 hours then supernatants were harvested and concentrated. TGF-β (**A**), IL1-β (**B**), C3 (**C**) and C3a (**D**) concentrations were quantified in cell cultured supernatants as well as in medium alone (Ø) (TGF-β, IL1-β, C3a by CBA and C3 by ELISA). Data are represented as mean ± SEM of measurements (n=5-7 for TGF-β, n=7-16 for IL1-β, n=8 for C3 and n=4-10 for C3a). One-way ANOVA statistical test was used, *p < 0.05, ***p < 0.001, ****p < 0.0001.

Supplementary Figure 8: Expression of complement components C1R and C1S, and C3aR in melanoma cell culture supernatants-treated MCs

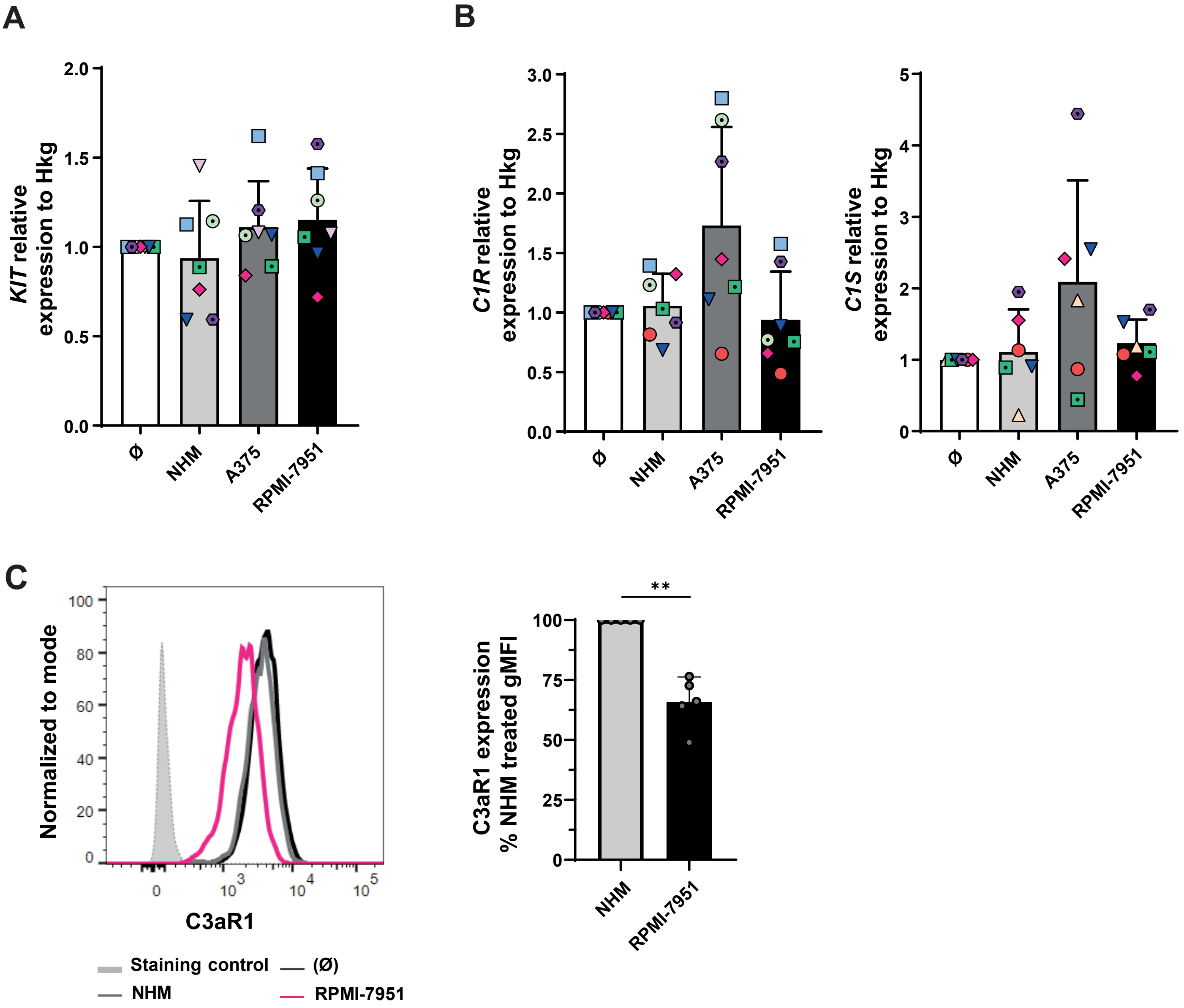

Supplementary Figure 8: Expression of component C1R and C1S, and C3aR in melanoma cell supernatant-treated MCs. Mast cells were incubated for 48 hours with melanoma cell supernatants (A375 and RPMI-7951), melanocyte supernatant (NHM) or medium alone (Ø). mRNA level of KIT (used here as comparison) (**A**) and complement component C1R and C1S (**B**) were measured by real-time RT-PCR and relatively expressed as a mean of 3 housekeeping genes (*PPIA*, *18S* and *H3-3B*). Data were normalised to medium treated control samples (Ø). Results are represented as mean ± SEM of at least 6 independent experiments (n=6-7). **C**, C3aR1 expression was measured by surface flow cytometry staining. One the left graph, the dotted grey histogram shows the control staining, the black line displays MCs treated with medium alone (Ø), the grey line represents MCs incubated with NHM culture supernatant and the pink line the ones treated with RPMI-7951 culture supernatant. On the right side in **C**, the graph shows the percentage of C3aR1 expression, values were calculated as percentage of geometric mean fluorescence intensity (gMFI) in NHM culture supernatant treated MCs. Each bar indicates mean ± SEM of 5 independent experiments (n=5), statistical comparisons were performed using Mann-Whitney U-test, $^{**}p < 0.01$.

Supplementary Table 1: Melanoma patient cohort samples used for immunohistochemistry

Patient ID	Gender	Age	Stage of Melanoma	Type of Melanoma	Sample location
1	F	73	III	nodular	border
2	M	69	NA	nodular	border
3	F	53	IV	nodular	border
4	F	78	III	acral lentiginous	center
5	M	70	NA	nodular	border
6	M	67	NA	nodular	center
7	M	68	NA	nodular	border
8	M	73	NA	nodular	border
9	M	60	III	SSM	border
10	M	71	IV	nodular	border
11	M	74	NA	SSM	border
12	M	59	NA	NA	center
13	F	76	NA	nodular	border/center
14	M	58	IV	nodular	border/center
15	F	49	IV	nodular	border
16	M	61	III	NA	border
17	F	72	NA	nodular	border/center
18	F	69	NA	nodular	center
19	M	70	NA	SSM	border
20	M	70	NA	SSM	border
21	M	79	NA	SSM	center
22	M	67	NA	nodular	center
23	M	55	NA	SSM	border
24	F	62	IV	NA	border/center
25	M	46	NA	NA	center
26	M	51	NA	SSM	border
27	M	51	NA	SSM	border
28	F	79	IV	nodular	border/center
29	M	77	NA	NA	center
30	M	29	III	nodular	border/center
31	M	65	IV	nodular	border/center
32	F	48	NA	SSM	border
33	F	84	IV	NA	border
34	F	69	NA	SSM	border/center
35	F	69	NA	SSM	border/center
36	M	50	NA	nodular	border/center
37	M	60	NA	NA	border/center
38	M	76	NA	SSM	center
39	F	81	NA	nodular	border

40	F	53	NA	NA	border/center
41	M	54	IV	nodular	center
42	F	70	NA	SSM	border
43	F	46	IV	nodular	border
44	F	56	NA	SSM	center
45	M	34	NA	SSM	border/center
46	M	76	NA	SSM	border
47	M	65	NA	SSM	center
48	F	45	NA	NA	border
49	M	47	IV	NA	center
50	M	58	NA	nodular	border
51	M	62	NA	SSM	border
52	M	80	NA	nodular	border/center
53	F	44	NA	NA	border
54	M	60	IV	SSM	center
55	M	82	NA	NA	border
56	M	56	IV	acral lentiginous	border/center
57	M	81	IV	SSM	border/center
58	M	74	NA	nodular	center
59	M	60	NA	acral lentiginous	center
60	F	82	IV	acral lentiginous	center
61	M	80	III	acral lentiginous	border
62	M	63	IV	nodular	border/center
63	M	46	III	SSM	border
64	F	78	III	SSM	border
65	F	67	NA	nodular	border
66	M	64	NA	SSM	border
67	M	64	IV	nodular	border
68	F	63	NA	SSM	border
69	M	82	NA	SSM	border
70	M	69	NA	SSM	border
71	F	53	NA	SSM	border
72	M	50	NA	SSM	border
73	F	34	NA	nodular	center
74	M	40	NA	NA	border
75	F	67	NA	nodular	border
76	M	71	III	nodular	border
77	F	39	NA	NA	border
78	M	22	NA	SSM	border
79	M	78	NA	SSM	border
80	M	64	NA	SSM	border
81	F	76	NA	SSM	border
82	M	53	NA	SSM	center
83	M	64	NA	SSM	border/center

84	M	73	NA	nodular	border/center
85	M	59	NA	SSM	border
86	F	45	NA	SSM	center
87	F	46	NA	nodular	border
88	F	58	IV	nodular	center
89	M	66	III	nodular	border/center
90	F	75	III	nodular	center
91	F	63	NA	nodular	border
92	M	≥60	III	cutaneous	border/center
93	M	≥60	IV	cutaneous	center
94	M	≥60	III	cutaneous	center
95	M	≥60	IV	cutaneous	center
96	M	<45	IV	cutaneous	border
97	M	≥60	IV	cutaneous	center
98	F	≥60	III	cutaneous	center
99	M	≥60	IV	cutaneous	center
100	F	≥60	IV	acral lentiginous	center
101	F	≥60	III	cutaneous	center
102	M	≥60	IV	unknown	center
103	M	≥60	III	cutaneous	border
104	M	<45	III	cutaneous	center
105	F	45-59	III	cutaneous	center
106	F	<45	IV	cutaneous	center
107	F	≥60	III	cutaneous	border/center
108	M	≥60	IV	acral lentiginous	border
109	M	≥60	IV	acral lentiginous	center
110	M	≥60	IV	acral lentiginous	center
111	F	45-59	III	cutaneous	center
112	F	45-59	IV	ocular	center
113	M	≥60	III	cutaneous	center
114	M	45-59	III	cutaneous	border/center
115	F	≥60	NA	mucosal	center
116	F	≥60	IV	cutaneous	center
117	M	≥60	III	cutaneous	center
118	M	≥60	III	cutaneous	border/center
119	F	≥60	III	cutaneous	center
120	M	<45	IV	cutaneous	center
121	M	45-59	IV	cutaneous	center
122	F	≥60	IV	acral lentiginous	center
123	F	≥60	IV	acral lentiginous	border/center
124	F	≥60	IV	acral lentiginous	center
125	F	≥60	IV	cutaneous	border
126	M	≥60	III	cutaneous	border/center
127	F	45-59	IV	ocular	center

128	M	≥60	IV	cutaneous	center
129	M	≥60	IV	cutaneous	border
130	M	≥60	III	unknown	center
131	M	<45	IV	cutaneous	center
132	M	<45	IV	cutaneous	border/center
133	F	≥60	IV	acral lentiginous	center
134	M	45-59	IV	cutaneous	center
135	M	≥60	IV	cutaneous	center
136	M	≥60	IV	cutaneous	center
137	M	<45	III	cutaneous	center
138	M	45-59	IV	cutaneous	center
139	M	45-59	IV	cutaneous	border
140	F	≥60	III	cutaneous	center
141	M	<45	IV	cutaneous	center
142	F	45-59	IV	cutaneous	center
143	M	45-59	III	cutaneous	center
144	F	≥60	III	cutaneous	center
145	F	≥60	III	cutaneous	center
146	M	≥60	IV	cutaneous	center
147	F	45-59	IV	cutaneous	center
148	M	≥60	IV	cutaneous	center

F: female, M: male, NA: not available, SSM: superficial spreading melanoma

Supplementary Table 2: Forward and reverse primer sequences used for RT-qPCR analysis

Gene symbol	Gene name	Sequence	Primer sequence (5'-3')	Source
PPIA	Peptidyl-prolylisomerase A	Forward	GGCAAATGCTGGACCAAAC	(1)
		Reverse	CATTCCTGGACCCAAAACG	
H3-3B	H3.3 histone B	Forward	TGCTGGTTTTTCGCTCGTCG	(2)
		Reverse	GCCATTTTCTTTCACCCAACGC	
18S	18S ribosomal RNAs	Forward	GTAACCCGTTGAACCCCATT	(3)
		Reverse	CCATCCAATCGGTAGTAGCG	
TPSAB	Tryptase alpha/beta	Forward	TCATGCCAGGGCGACTCCG	(4)
		Reverse	ACCCAGGTGGACACCCCAGG	
CMA1	Chymase 1	Forward	CCGGTAATGGGAGATTCGGG	
		Reverse	GGGCAATCCCAGGAAGACAA	
CPA3	Carboxypeptidase A3	Forward	AGGATGAAAAACAAGCAGACATCA	(4)
		Reverse	CAGACTGGATGGCTTGGGATT	
FCER1A	FcεRIa	Forward	TGTGGCAGCTGGACTATGAGTCT	(4)
		Reverse	ACTTCTCACGCGGAGCTTTTAT	
MS4A2	Membrane spanning 4-domains A2	Forward	AATCTTGCTCTCCCACAGGA	(5)
		Reverse	TGTGTTACCCCCAGGAACTC	
IL1B	Interleukin 1 beta	Forward	CTCGCCAGTGAAATGATGGCT	(6)
		Reverse	GTCGGAGATTCGTAGCTGGAT	
C5	Complement C5	Forward	ACTGAATTTGGTTGCTACTCCTC	(7)
		Reverse	GTATTACTGGGACTCCTCCTACC	
C3	Complement C3	Forward	GAGCCAGGAGTGGACTATGTGTA	(8)
		Reverse	CAATGGCCATGATGTACTCG	
C1R	Complement C1r	Forward	GCCTCCCTGACAACGATACCTTCTA	(9)
		Reverse	CGTCCTGCTTTAGAGATGGGTGTCC	
C1S	Complement C1s	Forward	AAGAGCGTTTTACGGGGTTT	(10)
		Reverse	AATCTCCCCAATCAGTGCAG	
KIT	KIT proto-oncogene, receptor tyrosine kinase	Forward	ATTTTCTCTGCGTTCTGCTCCTAC	(4)
		Reverse	CGCCCACGCGGACTATTA	

Supplementary Table 3: Gene list for HSMC, MAMC and BCCMC Venn Diagram intersections

243 elements included exclusively in "Down in MAMCs vs HSMCs"	347 elements included exclusively in "Up in MAMCs vs HSMCs	2 common elements in "Up in MAMCs vs HSMCs" and "Down in BCCMCs vs HSMCs"	240 elements included exclusively in "Down in BCCMCs vs HSMCs"	194 elements included exclusively in "Up in BCCMCs vs HSMCs"	27 common elements in "Up in MAMCs vs HSMCs" and "Up in BCCMCs vs HSMCs"	20 common elements in "Down in MAMCs vs HSMCs" and "Down in BCCMCs vs HSMCs"	3 common elements in "Down in MAMCs vs HSMCs" and "Up in BCCMCs vs HSMCs":
AAMP	*A2M*	*TCF7L2*	*AARS*	*AC111188.1*	*JUN*	*S100A2*	*FCER1A*
ABHD14A-ACY1	*ABCA7*	*APP*	*ABCB6*	*AC244502.1*	*SOX4*	*KRT5*	*C1orf186*
ADGRE2	*ABCC3*		*ABCF2*	*ACBD3*	*PLA2G4C*	*C10orf128*	*ARHGDIB*
ADIPOR1	*AC004594.1*		*ABLIM1*	*ACOT7*	*IKZF3*	*HMOX1*	
ADORA2B	*AC012313.3*		*ADGRG6*	*ADAP1*	*SERPINH1*	*SHISA2*	
ADRB2	*AC012358.3*		*AGPAT5*	*ADCY3*	*PER1*	*PDCD2*	
ADRM1	*AC098934.1*		*AKR1C1*	*ADCYAP1*	*ST3GAL5*	*PERP*	
AKR1B1	*AC108471.2*		*AKR1C2*	*ADGRE5*	*LIMK2*	*GBE1*	
AKT1	*AC109466.1*		*ALDH1A3*	*AGTRAP*	*LINC00511*	*FOSL1*	
ALDH1A1	*AC245060.5*		*ALDH3A1*	*AHR*	*LINC01125*	*PTDSS2*	
ANTXR2	*ACP5*		*ALG1L13P*	*AKAP12*	*PLCB2*	*SLC44A1*	
ANXA1	*ACSBG1*		*ALKAL2*	*AL157895.1*	*GSAP*	*TUBB4B*	
AP1G2	*ADA2*		*AMOTL2*	*ALOX5*	*CSAD*	*GAR1*	
AP2M1	*ADAM22*		*ANO6*	*ALOX5AP*	*ISG15*	*TUBA1C*	
AP3S1	*AEBP1*		*ANXA3*	*AMT*	*NLRC5*	*CTSL*	
APMAP	*AIF1L*		*AQP2*	*ANKRD28*	*CADPS*	*LDB2*	
AREG	*AL109976.1*		*AQP3*	*AP000648.3*	*SEMA4A*	*SLC38A2*	
ARF1	*AL136311.1*		*ARAP2*	*APOC2*	*SERPINF1*	*DPH2*	
ARF4	*AL139220.2*		*ARHGEF5*	*ARHGEF1*	*CPNE5*	*GLO1*	
ARF6	*AL356273.3*		*ASNS*	*ARHGEF6*	*SNX10*	*ANXA2*	
ARL4C	*AL512646.1*		*AZGP1*	*ARL5B*	*DIO2*		
ARL6IP5	*AL590094.1*		*B3GALT6*	*ASIC3*	*ITGB2*		
ATP6V0E1	*ALDH7A1*		*BNIP3*	*ASRGL1*	*MYEF2*		
ATP6V1A	*ANK2*		*BYSL*	*ATP2A3*	*ATG9B*		
BAG1	*ANKIB1*		*C15orf41*	*ATP2C1*	*ITGA2B*		
BET1L	*ANKS1B*		*C3orf52*	*ATP6V0A2*	*FYB1*		
BEX4	*ANP32B*		*C6orf141*	*BAG3*	*SPP1*		
BID	*AP000347.1*		*CA12*	*BCL2A1*			
BLOC1S2	*AP001486.2*		*CAPN2*	*BHLHE40*			
BLOC1S4	*AP2A2*		*CAV1*	*BST2*			
BRK1	*APOC1*		*CAV2*	*BTG2*			
BSPRY	*APOD*		*CCL20*	*BTK*			
C21orf91	*APOL1*		*CD163*	*BTN2A2*			
CA2	*APOL4*		*CD3EAP*	*C1orf162*			
CALM2	*ARHGDIA*		*CD59*	*C1orf228*			
CCL22	*ARNT2*		*CDH1*	*CACNA1H*			
CCNY	*ASF1B*		*CDH13*	*CCNH*			

CCT7	*ASPH*		*CDH3*	*CD37*			
CD44	*ATRNL1*		*CERS3*	*CD69*			
CD55	*AURKB*		*CHAC1*	*CD81*			
CD82	*AXL*		*CHL1*	*CD83*			
CDC42EP3	*BAIAP3*		*CLDN4*	*CDK15*			
CDK16	*BCAN*		*COCH*	*CENPM*			
CDK2AP2	*BIRC5*		*COL17A1*	*CHORDC1*			
CHPT1	*BMPR1B*		*COLEC11*	*CHRDL2*			
CIAO1	*BTN3A3*		*CRB3*	*CHRNA7*			
CLK4	*C10orf90*		*CSF3*	*CLNK*			
CNBP	*C11orf95*		*CYP1B1*	*CLU*			
CNIH1	*C19orf44*		*CYP3A5*	*CMA1*			
COMMD2	*C1R*		*DIEXF*	*CMPK2*			
CPM	*C1S*		*DMKN*	*COL9A2*			
CPPED1	*C20orf194*		*DNASE1L3*	*CPA3*			
CST7	*C3*		*DOCK7*	*CREB5*			
CXXC1	*C5orf42*		*DSG1*	*CSF1*			
CYC1	*CACNA1A*		*DST*	*CSF2RB*			
DAZAP2	*CAD*		*DUOX2*	*CST3*			
DCTD	*CALR*		*DUSP4*	*CTSG*			
DDIT4	*CAMK2B*		*EBNA1BP2*	*CYBA*			
DDX39A	*CAMK2D*		*ECE2*	*CYTH3*			
DEGS1	*CAPS2*		*EEF2KMT*	*DNAJA1*			
DNAJB11	*CASC19*		*EFEMP1*	*DNAJA4*			
DOHH	*CASP4*		*EGLN3*	*DNAJB4*			
DPH5	*CATSPERG*		*EHF*	*DNAJB6*			
DRAM2	*CCDC40*		*EIF2S1*	*DOK2*			
DRD2	*CCDC80*		*ENO1*	*DUSP1*			
DSG3	*CD36*		*EPHA5*	*EMID1*			
EED	*CDH11*		*EPN3*	*ETV6*			
EIF4H	*CDK18*		*ERRFI1*	*ETV7*			
EIF5A2	*CDR2L*		*EXO1*	*FAM46A*			
ERP44	*CENPF*		*FAM110B*	*FBP1*			
EXOSC1	*CEP112*		*FAM160A1*	*FDX1*			
FRS2	*CEP70*		*FBLIM1*	*FDXR*			
GFI1	*CFHR2*		*FGF7*	*FHL1*			
GHITM	*CFLAR-AS1*		*FGFRL1*	*FOS*			
GLUL	*CHD4*		*FHL2*	*FREM1*			
GNAI1	*CMIP*		*FRMD6*	*GADD45B*			
GNPAT	*CNN1*		*FTH1P2*	*GHRL*			
GNS	*CNTN2*		*FTH1P8*	*GLIPR2*			
GOLPH3	*COL11A1*		*FXYD3*	*GNA13*			
GPR65	*COL14A1*		*GAPDH*	*GPBP1*			
GPRC5C	*COL16A1*		*GBAP1*	*GRAP2*			
H2AFZ	*COL6A2*		*GCLM*	*GRASP*			
H3F3B	*COL6A3*		*GDF15*	*GRHL1*			

HDC	*COL9A3*		*GEMIN5*	*GRK2*
HDHD5	*CPT1C*		*GFPT2*	*HAAO*
HEY1	*CREB3L1*		*GJB3*	*HAVCR2*
HK1	*CSGALNACT1*		*GLIS3*	*HES4*
HLA-E	*CTSS*		*GNLY*	*HIPK1*
HNRNPF	*CUBN*		*GPATCH4*	*HIST1H2BG*
HPGD	*CYP17A1*		*GPI*	*HS3ST1*
HPGDS	*CYP27A1*		*GRHL3*	*HSP90AA1*
HSPA8	*DAB2*		*GULP1*	*HSPA6*
ID2	*DCC*		*GZMB*	*HSPB1*
IFNGR1	*DDIAS*		*HACD3*	*HSPD1*
IL2RG	*DDX25*		*HAS3*	*HSPG2*
ILF2	*DIAPH3*		*HBEGF*	*HSPH1*
ING4	*DLGAP1*		*HOPX*	*IER5*
IP6K2	*DMC1*		*HPSE*	*IGKV1-8*
ITM2A	*DNAH14*		*HPSE2*	*IGKV3D-11*
JUP	*DNASE1*		*HSPB8*	*IGLV1-41*
KIT	*DOC2A*		*IGFBP3*	*IL18BP*
KRT1	*DOCK4*		*IL1R2*	*ITGB7*
LAPTM4A	*DRC7*		*IL33*	*ITM2C*
LDHA	*DUXAP10*		*IMPA2*	*JUND*
LGALS3	*DYNLL2*		*IPO5*	*KMT5C*
LITAF	*ELK3*		*IRF6*	*LAIR1*
LPXN	*EML1*		*ITGA2*	*LIMD2*
LYPLA2	*EML6*		*ITGA6*	*LINC00694*
M6PR	*ENAH*		*KCNK1*	*LINC00893*
MAPRE1	*EPB41L2*		*KIAA1217*	*LMNA*
MED21	*ERBB3*		*KLC3*	*LTC4S*
MEG3	*EREG*		*KLF5*	*MAFF*
MELTF	*ESPN*		*KLK11*	*MAOB*
METTL9	*ESRRG*		*KLK5*	*MAP3K1*
MFSD8	*ETV1*		*KRT16*	*MAST4*
MOB1A	*FADS6*		*LAD1*	*MCTP1*
MPP1	*FAM19A5*		*LAMA3*	*MIIP*
MRGPRX2	*FANCA*		*LAMA4*	*MRPL55*
MRPL15	*FBN2*		*LAMB4*	*MS4A3*
MRPS26	*FHAD1*		*LAMC2*	*MSRA*
MSRB2	*FMO1*		*LARS2*	*MYADM*
MUL1	*FN1*		*LCE3A*	*MYO15B*
MYD88	*GABBR2*		*LGALS12*	*MYO5C*
MYL12A	*GABPB2*		*LIMA1*	*NCF1*
MYL12B	*GABRD*		*LINC00969*	*NDST2*
NAA50	*GARNL3*		*LPP*	*NEDD9*
NABP1	*GAS7*		*LRRC49*	*NFATC1*
NANS	*GBA3*		*LXN*	*NFE2*
NCBP2-AS2	*GCNT2*		*LYPD3*	*NFKBID*

NDUFA8	*GFAP*		*LZTFL1*	*NFKBIE*			
NDUFAF4	*GMPR*		*MAF*	*NFKBIZ*			
NHEJ1	*GOLGA8B*		*MAGI2*	*NR4A1*			
NOP2	*GPM6A*		*MAL2*	*NR4A2*			
NT5C3B	*GPM6B*		*MAOA*	*NR4A3*			
ODC1	*GPR155*		*MET*	*PARVG*			
OR14L1P	*GRM3*		*MGST1*	*PBX4*			
OSER1	*GYG2*		*MIAT*	*PDXK*			
OSM	*HAGLR*		*MIR205HG*	*PHACTR1*			
OSTF1	*HERC2P3*		*MIR22HG*	*PHF20*			
P2RY1	*HFE*		*MME*	*PIM2*			
PANX1	*HGNC:24955*		*MMP3*	*PLAT*			
PCNP	*HIF3A*		*MPZL2*	*PLAUR*			
PDHB	*HIST2H2AC*		*MS4A6A*	*PLEK*			
PDLIM5	*HLA-F*		*MT1G*	*PLK3*			
PEBP1	*HORMAD1*		*MT1X*	*POU2F2*			
PFN1	*HOXB3*		*MT2A*	*PPP1R15A*			
PHAX	*HOXB-AS3*		*MTND1P23*	*PPP1R15B*			
PITPNA	*ICA1L*		*MUCL1*	*PTPN6*			
PLEKHB2	*IGF2*		*MXI1*	*RAB32*			
PLIN2	*IGF2BP3*		*MYO1B*	*RAB33A*			
PLP2	*IGFBP4*		*NAV3*	*RAB37*			
PNMA1	*IGFBP5*		*NDRG1*	*RANBP2*			
POLR2D	*INHBA*		*NHP2*	*RASGRP2*			
PPIA	*INTU*		*NOL10*	*RGCC*			
PPP2R1A	*IQCE*		*NOLC1*	*RGS1*			
PPP4C	*ITGAL*		*NOP16*	*RGS10*			
PRDX3	*JAK3*		*NOP56*	*RGS3*			
PREB	*JAML*		*NR2F2*	*RHOH*			
PRNP	*KCNQ1OT1*		*NRG1*	*RILPL2*			
PRPF8	*KCTD12*		*P3H2*	*RIN3*			
PSMB6	*KIAA1524*		*PAICS*	*RNF166*			
PSMD13	*KIF3C*		*PAM*	*ROBO3*			
PSME3	*KLF2*		*PCAT29*	*RRAD*			
PSMF1	*KNL1*		*PCDH7*	*RSAD2*			
PTDSS1	*KNTC1*		*PCK2*	*SAMSN1*			
RAB10	*LAMB1*		*PDLIM3*	*SAP25*			
RAB38	*LAMC1*		*PHLDB2*	*SEPT4*			
RAC2	*LGALS3BP*		*PIGN*	*SERPINB9*			
RAE1	*LGMN*		*PIK3AP1*	*SH3TC1*			
RASSF2	*LHFPL2*		*PLA2G2A*	*SKIL*			
RGS2	*LHFPL3-AS1*		*PLS1*	*SLC18A2*			
RHOG	*LIMK1*		*PLS3*	*SLC45A3*			
RIC8A	*LINC01198*		*POLR1B*	*SMAP2*			
RIOX2	*LINC01697*		*PPA1*	*SOCS1*			
RNH1	*LINC02028*		*PPP2R5A*	*SPI1*			

RPN1	LMNTD1		PRMT7	SREBF1			
RPS6KB2	LMO3		PRSS3	STAP1			
RTCB	LNPK		PSG4	SYAP1			
S100A11	LOX		PUS7	TANK			
SCAMP2	LOXL2		RAPH1	TCTEX1D1			
SDCBP	LRTM2		RARRES1	THY1			
SEC23B	MAFB		RBM47	TMC8			
SEC61B	MAGI1		RBPMS	TMEM173			
SERINC1	MAP1B		RCAN1	TMEM176B			
SGK1	MAPK10		RGMB	TNFAIP3			
SH3BGRL3	MARCKS		RGS5	TNFRSF21			
SIGLEC6	MBD5		RNF144B	TNFSF10			
SLC25A38	MECOM		RRP9	TSC22D1			
SLC25A5	MEF2C		S100A16	TSPYL2			
SLC36A4	MEFV		SCUBE1	TUBA1A			
SLC39A8	MEIS1		SDR16C5	UBA7			
SLC3A2	MEST		SEMA4B	ZBTB32			
SLC40A1	MFGE8		SEPT10	ZC3H12A			
SLC6A6	MIA		SERPINB2	ZFAND2A			
SLCO2B1	MLANA		SERPINB5				
SNRPG	MMP14		SERPINB7				
SOCS3	MMP17		SHQ1				
SQSTM1	MPDZ		SLC25A23				
SRGN	MSR1		SLC25A27				
STK26	MTMR11		SLC2A1				
STMN1	MT-ND1		SLC35B4				
STX11	MT-ND3		SLC38A5				
SYK	MT-ND5		SLC38A9				
SYNGR2	MT-ND6		SLC39A14				
TAGLN2	MT-RNR1		SLC39A6				
TANGO2	MTRNR2L8		SLC5A6				
TBC1D30	MUC19		SMTN				
TEX30	MX1		SORBS2				
TFG	MX2		SORD				
TGIF1	NAV2		SOWAHC				
TLDC1	NDRG4		SPRR1B				
TM9SF2	NFATC4		SRM				
TMED2	NFE2L1		SRPRB				
TMED9	NLGN1		STAG3				
TMEM218	NR1H3		STAP2				
TMEM60	NR1I3		SULT2B1				
TMX1	NUCKS1		SYNGR3				
TPRKB	NUF2		SYT8				
TPSD1	NXN		TFPI				
TPST2	OSBP2		THBS2				
TSG101	PAK3		THOC5				

TSR2	PARP14		TM4SF1				
TXN	PARP8		TMEM38B				
TXNIP	PAX6		TMEM40				
UBE2J1	PCOLCE2		TMEM45A				
UBLCP1	PEG10		TNFRSF25				
UFM1	PELP1		TOMM40				
ULK3	PFKM		TPD52L1				
USP1	PGGHG		TRIM16L				
VDAC3	PHF14		TRIM29				
VPS28	PHLDB1		TRPV3				
VPS29	PKD2		TWNK				
VWA5A	PLCB1		TXNRD1				
WDR12	PLCD4		WASF1				
YWHAZ	PLEKHA4		WWTR1				
ZFYVE21	PLEKHA6		ZBTB38				
ZNF181	PLEKHG2		ZDHHC16				
ZNF542P	PLK4		ZNF185				
ZNF622	PLOD2		ZNF229				
ZNF667-AS1	PLP1		ZNF692				
ZNF675	PLXNA2						
ZNF706	PLXND1						
ZPR1	PMEL						
	POLG2						
	PPFIA1						
	PPFIA3						
	PPFIBP1						
	PPM1N						
	PRC1						
	PRR11						
	PSPH						
	PSRC1						
	PSTPIP2						
	PTPRD						
	PTPRM						
	QPCT						
	RAD54B						
	RAP2A						
	RASGRP3						
	RASSF3						
	RASSF4						
	RBM25						
	RCAN3						
	RFX4						
	RGS16						
	RHOB						
	RIMS2						

	RIPOR2						
	RNF144A						
	ROCK1						
	SAMMSON						
	SCARB1						
	SCUBE2						
	SEC14L3						
	SEMA3B						
	SEMA3C						
	SERPINE2						
	SFRP2						
	SHCBP1						
	SKI						
	SLC11A1						
	SLC12A8						
	SLC12A9						
	SLC15A3						
	SLC16A3						
	SLC26A8						
	SLC5A9						
	SLC7A7						
	SLC8A1-AS1						
	SLITRK5						
	SOX2-OT						
	SOX5						
	SP100						
	SP140						
	SP140L						
	SPAG9						
	SPIRE2						
	SPTAN1						
	SPTBN1						
	SRPX2						
	STMN3						
	STOX2						
	STS						
	SULF1						
	SULF2						
	SYNGR1						
	SYNPR						
	SYP						
	SYT14						
	TBC1D16						
	TBC1D28						
	TBC1D31						
	TCEAL9						

	TDRD9						
	TECPR1						
	TEX41						
	TFRC						
	TGFBR3						
	THBS3						
	TMCC2						
	TMEM47						
	TNC						
	TNFAIP2						
	TNRC18						
	TOMM7						
	TPM2						
	TRA2A						
	TRA2B						
	TRABD2A						
	TRIM2						
	TRIM41						
	TRPM4						
	TYMS						
	UACA						
	UBALD2						
	UNC5D						
	USP36						
	VCAN						
	VEPH1						
	WISP2						
	ZBTB10						
	ZCCHC24						
	ZNF175						
	ZNF397						
	ZNF532						
	ZNF608						

HSMCs: healthy skin-associated mast cells, BCCMCs: basal cell carcinoma-associated mast cells, MAMCs: melanoma-associated mast cells.

Supplementary Table 4: Top 50 highly expressed transcripts in skin MCs

gene	gene_id	locus	expression in HSMCs (FPKM)	expression in BCCMCs (FPKM)	expression in MAMCs (FPKM)
CD44	ENSG00000026508.17	chr11:35138869-35232402	1764.63	2054.42	387.432
CAPG	ENSG00000042493.15	chr2:85394747-85418432	800.685	1434.11	330.91
KLF6	ENSG00000067082.14	chr10:3775995-3785281	552.539	1462.17	524.565
ATP1B3	ENSG00000069849.10	chr3:141876123-141926514	611.447	1102.67	434.341
ACTB	ENSG00000075624.13	chr7:5527150-5563784	2674.85	1671.13	1032
HSP90AA1	ENSG00000080824.18	chr14:102080737-102305200	1287.64	5492.62	4017.29
CD82	ENSG00000085117.11	chr11:44564426-44620363	928.386	873.462	113.778
FTL	ENSG00000087086.14	chr19:48965300-48966878	11730.9	8033.79	4438.43
CMA1	ENSG00000092009.10	chr14:24501593-24508688	711.153	1893.29	255.158
HSP90AB1	ENSG00000096384.19	chr6:44246165-44253888	905.053	1931.66	631.594
GADD45B	ENSG00000099860.8	chr19:2476121-2478259	506.762	1334.29	271.764
LGALS1	ENSG00000100097.11	chr22:37675607-37679806	698.765	763.065	848.336
CTSG	ENSG00000100448.3	chr14:24573521-24576260	3493.61	9646.37	1228.71
HSPB1	ENSG00000106211.8	chr7:76302543-76304295	312.639	1263.52	491.767
HSPA8	ENSG00000109971.13	chr11:123057488-123063230	1196.21	2006.14	209.185
CD69	ENSG00000110848.8	chr12:9752485-9760901	153.448	1442.1	113.958
ARHGDIB	ENSG00000111348.8	chr12:14942016-14961728	690.544	1563.57	154.424
GAPDH	ENSG00000111640.14	chr12:6533926-6538374	1880.37	659.471	597.488
HSPE1	ENSG00000115541.10	chr2:197486580-197553699	458.067	1565.09	365.217
DUSP1	ENSG00000120129.5	chr5:172758225-172777774	263.604	1436.47	197.194
HSPH1	ENSG00000120694.19	chr13:31134973-31162388	184.603	1053.28	708.107
CLU	ENSG00000120885.21	chr8:27596916-27615031	377.903	1513.78	764.042
LGALS3	ENSG00000131981.15	chr14:55124109-55145413	2039.89	1258.46	366.664
DNAJB1	ENSG00000132002.7	chr19:14514769-14565980	312.217	3433.51	421.444
H3F3B	ENSG00000132475.10	chr17:75776433-75825799	3702.52	7263.45	842.415
ANXA1	ENSG00000135046.13	chr9:73151756-73170393	2554.43	4597.87	442.189
CD63	ENSG00000135404.11	chr12:55725322-55730852	2014.73	2858.22	2040.24
GLUL	ENSG00000135821.17	chr1:182381703-182392206	6767.57	5396.97	239.277
HDC	ENSG00000140287.10	chr15:50241946-50266026	2706.11	3561.21	437.861
EMP3	ENSG00000142227.10	chr19:48296456-48330553	1008.47	913.174	364.711
CALM2	ENSG00000143933.16	chr2:46899274-47176601	978.582	999.507	272.164
HSPD1	ENSG00000144381.16	chr2:197486580-197553699	228.975	1041.71	420.429
EEF1A1	ENSG00000156508.17	chr6:73515749-73570596	866.255	804.226	385.182
FCER1G	ENSG00000158869.10	chr1:161215233-161220699	987.017	1638.97	286.5
LMNA	ENSG00000160789.19	chr1:156082572-156140089	1468.98	3838.88	635.774
HPGDS	ENSG00000163106.10	chr4:94298534-94342876	1012.18	1184.78	80.9123
CPA3	ENSG00000163751.3	chr3:148791101-148960112	861.826	3534.78	629.941
HPGD	ENSG00000164120.13	chr4:174490176-174523154	1914.41	1011.02	176.012
B2M	ENSG00000166710.17	chr15:44711476-44718877	3843.42	8929.23	1537.45
FTH1	ENSG00000167996.15	chr11:61949820-61967660	30168.2	25922.2	5065.48

DDIT4	ENSG00000168209.4	chr10:72273919-72276036	952.34	826.508	141.56
FOS	ENSG00000170345.9	chr14:75278773-75282230	348.137	1229.78	802.954
CFL1	ENSG00000172757.12	chr11:65823021-65873592	855.458	750.745	309.512
EIF1	ENSG00000173812.10	chr17:41688884-41692668	1445.45	1086.14	397.736
ACTG1	ENSG00000184009.9	chr17:81509970-81527776	1209.82	448.803	355.468
CALM1	ENSG00000198668.10	chr14:90396501-90408261	1355.73	840.229	553.042
IGKC	ENSG00000211592.8	chr2:88811185-88861563	1839.84	7491.2	58.8444
IGLC1	ENSG00000211675.2	chr22:22887779-22896107	626.526	2002.77	12.9687
MALAT1	ENSG00000251562.7	chr11:65497761-65506516	40223.1	55696.4	64876
MIR1248	ENSG00000283958.1	chr3:186781779-186807058	1242.44	1220.79	15879.4

HSMCs: healthy skin-associated mast cells, BCCMCs: basal cell carcinoma-associated mast cells, MAMCs: melanoma-associated mast cells. FPKM: fragments per kilo base of transcript per million mapped fragments.

Supplementary Table 5: Gene expression of FcεRI signalling-associated molecules in skin MCs

gene	gene_id	locus	sample_1	sample_2	status	value_1 (FPKM)	value_2 (FPKM)	log2 (fold_change)	test_stat	p_value	q_value	significant
AKT1	ENSG00000142208.15	chr14:104769348-104795751	HSMCs	MAMCs	OK	44.282	5.24883	-3.07665	-1.62916	5.00E-05	0.005242	yes
AKT1	ENSG00000142208.15	chr14:104769348-104795751	BCCMCs	HSMCs	OK	37.6084	44.282	0.235666	0.160546	0.5159	0.670453	no
AKT1	ENSG00000142208.15	chr14:104769348-104795751	BCCMCs	MAMCs	OK	37.6084	5.24883	-2.84099	-1.52047	5.00E-05	0.005242	yes
BTK	ENSG00000010671.15	chrX:101349446-101390796	HSMCs	MAMCs	OK	45.0459	34.0529	-0.403619	-0.237893	0.48555	0.647466	no
BTK	ENSG00000010671.15	chrX:101349446-101390796	BCCMCs	MAMCs	OK	143.306	34.0529	-2.07325	-1.25512	6.00E-04	0.0282057	yes
BTK	ENSG00000010671.15	chrX:101349446-101390796	BCCMCs	HSMCs	OK	143.306	45.0459	-1.66963	-1.02408	5.00E-05	0.005242	yes
CSF2	ENSG00000164400.5	chr5:132073789-132076170	BCCMCs	HSMCs	OK	14.4566	65.2384	2.17399	0.913683	0.3465	0.553542	no
CSF2	ENSG00000164400.5	chr5:132073789-132076170	HSMCs	MAMCs	OK	65.2384	28.111	-1.21459	-0.557211	0.679	0.792637	no
CSF2	ENSG00000164400.5	chr5:132073789-132076170	BCCMCs	MAMCs	OK	14.4566	28.111	0.959406	0.424221	0.71585	0.81896	no
FCER1A	ENSG00000179639.10	chr1:159289713-159308224	BCCMCs	HSMCs	OK	562.876	249.526	-1.17363	-1.02986	5.00E-04	0.025397	yes
FCER1A	ENSG00000179639.10	chr1:159289713-159308224	BCCMCs	MAMCs	OK	562.876	15.442	-5.18789	-3.34008	5.00E-05	0.005242	yes
FCER1A	ENSG00000179639.10	chr1:159289713-159308224	HSMCs	MAMCs	OK	249.526	15.442	-4.01426	-2.54856	1.00E-04	0.00869469	yes
FCER1G	ENSG00000158869.10	chr1:161215233-161220699	HSMCs	MAMCs	OK	987.017	286.5	-1.78454	-1.57518	0.00175	0.0533494	no
FCER1G	ENSG00000158869.10	chr1:161215233-161220699	BCCMCs	MAMCs	OK	1638.97	286.5	-2.51619	-2.23943	5.00E-05	0.005242	yes
FCER1G	ENSG00000158869.10	chr1:161215233-161220699	BCCMCs	HSMCs	OK	1638.97	987.017	-0.731646	-0.713465	0.0316	0.231917	no
FYN	ENSG00000010810.17	chr6:111660331-111873452	BCCMCs	HSMCs	OK	178.969	103.997	-0.78317	-0.492512	0.02485	0.209341	no
FYN	ENSG00000010810.17	chr6:111660331-111873452	HSMCs	MAMCs	OK	103.997	169.459	0.704399	0.444847	0.1654	0.452007	no
FYN	ENSG00000010810.17	chr6:111660331-111873452	BCCMCs	MAMCs	OK	178.969	169.459	-0.078771	-0.0517789	0.87335	0.923659	no
GAB2	ENSG00000033327.12	chr11:78215296-78574874	BCCMCs	MAMCs	OK	4.97193	6.81973	0.45591	0.150265	0.50315	0.661028	no
GAB2	ENSG00000033327.12	chr11:78215296-78574874	BCCMCs	HSMCs	OK	4.97193	9.37231	0.9146	0.27779	0.0855	0.3506	no
GAB2	ENSG00000033327.12	chr11:78215296-78574874	HSMCs	MAMCs	OK	9.37231	6.81973	-0.458689	-0.144996	0.55775	0.70313	no
GRB2	ENSG00000177885.13	chr17:75318075-75405709	BCCMCs	MAMCs	OK	163.894	68.0503	-1.26809	-0.633341	0.05515	0.293186	no
GRB2	ENSG00000177885.13	chr17:75318075-75405709	HSMCs	MAMCs	OK	147.857	68.0503	-1.11953	-0.594036	0.09425	0.365021	no
GRB2	ENSG00000177885.13	chr17:75318075-75405709	BCCMCs	HSMCs	OK	163.894	147.857	-0.148565	-0.0886185	0.6975	0.806241	no
HRAS	ENSG00000174775.16	chr11:532241-537287	BCCMCs	MAMCs	OK	11.4752	9.01105	-0.348752	-0.196618	0.7048	0.81142	no
HRAS	ENSG00000174775.16	chr11:532241-537287	BCCMCs	HSMCs	OK	11.4752	17.0802	0.57381	0.346928	0.2791	0.520418	no
HRAS	ENSG00000174775.16	chr11:532241-537287	HSMCs	MAMCs	OK	17.0802	9.01105	-0.922562	-0.537011	0.37425	0.571814	no
IL13	ENSG00000169194.9	chr5:132410635-132664272	BCCMCs	MAMCs	OK	14.1336	10.6318	-0.410748	-0.0841577	0.86125	0.915905	no
IL13	ENSG00000169194.9	chr5:132410635-132664272	HSMCs	MAMCs	OK	37.6577	10.6318	-1.82457	-0.410879	0.3822	0.576418	no
IL13	ENSG00000169194.9	chr5:132410635-132664272	BCCMCs	HSMCs	OK	14.1336	37.6577	1.41382	0.448634	0.34185	0.550977	no
IL3	ENSG00000164399.4	chr5:132060528-132063204	HSMCs	MAMCs	NOTEST	0.177278	0.112291	-0.658768	0	1	1	no
IL3	ENSG00000164399.4	chr5:132060528-132063204	BCCMCs	HSMCs	NOTEST	0.15742	0.177278	0.171397	0	1	1	no
IL3	ENSG00000164399.4	chr5:132060528-132063204	BCCMCs	MAMCs	NOTEST	0.15742	0.112291	-0.487371	0	1	1	no
IL4	ENSG00000113520.10	chr5:132673985-132682676	BCCMCs	HSMCs	NOTEST	0.360512	0.502007	0.477659	0	1	1	no
IL4	ENSG00000113520.10	chr5:132673985-132682676	HSMCs	MAMCs	NOTEST	0.502007	0		0	1	1	no
IL4	ENSG00000113520.10	chr5:132673985-132682676	BCCMCs	MAMCs	NOTEST	0.360512	0		0	1	1	no
IL5	ENSG00000113525.9	chr5:132410635-132664272	BCCMCs	MAMCs	NOTEST	0.906368	0.89888	-0.0119698	0	1	1	no
IL5	ENSG00000113525.9	chr5:132410635-132664272	HSMCs	MAMCs	NOTEST	0.374556	0.89888	1.26295	0	1	1	no
IL5	ENSG00000113525.9	chr5:132410635-132664272	BCCMCs	HSMCs	NOTEST	0.906368	0.374556	-1.27492	0	1	1	no

INPP5D	ENSG00000168918.13	chr2:233059966-233207903	BCCMCs	MAMCs	OK	10.5424	0.860319	-3.61519	-1.19815	5.00E-05	0.005242	yes
INPP5D	ENSG00000168918.13	chr2:233059966-233207903	BCCMCs	HSMCs	OK	10.5424	5.22551	-1.01256	-0.395177	0.0308	0.229947	no
INPP5D	ENSG00000168918.13	chr2:233059966-233207903	HSMCs	MAMCs	OK	5.22551	0.860319	-2.60263	-0.816496	0.0016	0.0508268	no
KRAS	ENSG00000133703.11	chr12:25195215-25250936	BCCMCs	HSMCs	OK	34.8762	33.4281	-0.0611829	-0.0383667	0.9195	0.952231	no
KRAS	ENSG00000133703.11	chr12:25195215-25250936	HSMCs	MAMCs	OK	33.4281	11.424	-1.54899	-0.875049	0.0443	0.268198	no
KRAS	ENSG00000133703.11	chr12:25195215-25250936	BCCMCs	MAMCs	OK	34.8762	11.424	-1.61018	-0.905133	0.02835	0.221867	no
LAT	ENSG00000213658.11	chr16:28973961-28990783	BCCMCs	HSMCs	OK	366.614	335.168	-0.12938	-0.0941293	0.73235	0.830049	no
LAT	ENSG00000213658.11	chr16:28973961-28990783	BCCMCs	MAMCs	OK	366.614	98.3513	-1.89825	-1.1199	0.0095	0.131356	no
LAT	ENSG00000213658.11	chr16:28973961-28990783	HSMCs	MAMCs	OK	335.168	98.3513	-1.76887	-1.03694	0.01715	0.175991	no
LCP2	ENSG00000043462.11	chr5:170232446-170298227	BCCMCs	HSMCs	OK	114.836	53.0658	-1.11372	-0.770952	0.0027	0.0685212	no
LCP2	ENSG00000043462.11	chr5:170232446-170298227	HSMCs	MAMCs	OK	53.0658	51.8721	-0.0328235	-0.0219743	0.95595	0.973675	no
LCP2	ENSG00000043462.11	chr5:170232446-170298227	BCCMCs	MAMCs	OK	114.836	51.8721	-1.14654	-0.853364	0.05975	0.303113	no
LYN	ENSG00000254087.7	chr8:55879812-56014168	HSMCs	MAMCs	OK	24.4103	8.41189	-1.53699	-1.01269	0.0199	0.18774	no
LYN	ENSG00000254087.7	chr8:55879812-56014168	BCCMCs	HSMCs	OK	28.1099	24.4103	-0.203584	-0.14627	0.67805	0.791918	no
LYN	ENSG00000254087.7	chr8:55879812-56014168	BCCMCs	MAMCs	OK	28.1099	8.41189	-1.74057	-1.12714	0.0045	0.0902559	no
MAP2K1	ENSG00000169032.9	chr15:66386816-66497813	BCCMCs	MAMCs	OK	15.7779	9.24131	-0.771737	-0.480255	0.29305	0.526304	no
MAP2K1	ENSG00000169032.9	chr15:66386816-66497813	HSMCs	MAMCs	OK	24.3904	9.24131	-1.40014	-0.902088	0.06185	0.30724	no
MAP2K1	ENSG00000169032.9	chr15:66386816-66497813	BCCMCs	HSMCs	OK	15.7779	24.3904	0.628407	0.387132	0.29855	0.528541	no
MAP2K2	ENSG00000126934.13	chr19:4090320-4124129	BCCMCs	MAMCs	OK	30.6458	26.7944	-0.193758	-0.13716	0.7294	0.828111	no
MAP2K2	ENSG00000126934.13	chr19:4090320-4124129	HSMCs	MAMCs	OK	36.1738	26.7944	-0.433015	-0.307569	0.4781	0.642038	no
MAP2K2	ENSG00000126934.13	chr19:4090320-4124129	BCCMCs	HSMCs	OK	30.6458	36.1738	0.239257	0.173188	0.57235	0.714114	no
MAP2K3	ENSG00000034152.18	chr17:21284671-21315240	BCCMCs	HSMCs	OK	121.633	122.239	0.00716638	0.0059253	0.9837	0.990034	no
MAP2K3	ENSG00000034152.18	chr17:21284671-21315240	HSMCs	MAMCs	OK	122.239	104.181	-0.230606	-0.15737	0.7052	0.811691	no
MAP2K3	ENSG00000034152.18	chr17:21284671-21315240	BCCMCs	MAMCs	OK	121.633	104.181	-0.22344	-0.158063	0.6772	0.791261	no
MAP2K4	ENSG00000065559.14	chr17:11998352-12143830	BCCMCs	HSMCs	OK	11.0824	12.324	0.153197	0.0783025	0.71085	0.815355	no
MAP2K4	ENSG00000065559.14	chr17:11998352-12143830	HSMCs	MAMCs	OK	12.324	4.40591	-1.48396	-0.737048	0.0378	0.250597	no
MAP2K4	ENSG00000065559.14	chr17:11998352-12143830	BCCMCs	MAMCs	OK	11.0824	4.40591	-1.33076	-0.630485	0.0511	0.283717	no
MAP2K6	ENSG00000108984.13	chr17:69414697-69543331	HSMCs	MAMCs	OK	1.50704	7.1885	2.25397	0.747277	0.01955	0.185814	no
MAP2K6	ENSG00000108984.13	chr17:69414697-69543331	BCCMCs	HSMCs	OK	2.51862	1.50704	-0.740914	-0.243107	0.1877	0.468977	no
MAP2K6	ENSG00000108984.13	chr17:69414697-69543331	BCCMCs	MAMCs	OK	2.51862	7.1885	1.51306	0.504035	0.0873	0.353742	no
MAP2K7	ENSG00000076984.17	chr19:7903842-7914478	HSMCs	MAMCs	OK	2.85087	6.83888	1.26236	0.615204	0.0977	0.370272	no
MAP2K7	ENSG00000076984.17	chr19:7903842-7914478	BCCMCs	HSMCs	OK	4.04864	2.85087	-0.506034	-0.245481	0.41115	0.594451	no
MAP2K7	ENSG00000076984.17	chr19:7903842-7914478	BCCMCs	MAMCs	OK	4.04864	6.83888	0.756322	0.374681	0.32775	0.543738	no
MAPK1	ENSG00000100030.14	chr22:21754499-21867680	HSMCs	MAMCs	OK	79.3955	33.7516	-1.2341	-0.699573	0.1073	0.384953	no
MAPK1	ENSG00000100030.14	chr22:21754499-21867680	BCCMCs	HSMCs	OK	134.828	79.3955	-0.763994	-0.470464	0.14945	0.436155	no
MAPK1	ENSG00000100030.14	chr22:21754499-21867680	BCCMCs	MAMCs	OK	134.828	33.7516	-1.9981	-1.14496	0.00435	0.0888276	no
MAPK14	ENSG00000112062.10	chr6:36027676-36111236	HSMCs	MAMCs	OK	17.6325	13.4254	-0.393274	-0.222541	0.5506	0.697235	no
MAPK14	ENSG00000112062.10	chr6:36027676-36111236	BCCMCs	HSMCs	OK	23.4275	17.6325	-0.409966	-0.234365	0.35035	0.556204	no
MAPK14	ENSG00000112062.10	chr6:36027676-36111236	BCCMCs	MAMCs	OK	23.4275	13.4254	-0.80324	-0.489631	0.17345	0.459286	no
MAPK8	ENSG00000107643.15	chr10:48306638-48439360	BCCMCs	HSMCs	OK	14.7459	22.0183	0.578392	0.308047	0.309	0.533327	no
MAPK8	ENSG00000107643.15	chr10:48306638-48439360	BCCMCs	MAMCs	OK	14.7459	5.49239	-1.4248	-0.612697	0.08905	0.356942	no
MAPK8	ENSG00000107643.15	chr10:48306638-48439360	HSMCs	MAMCs	OK	22.0183	5.49239	-2.0032	-0.864753	0.02945	0.225749	no
MS4A2	ENSG00000149534.8	chr11:60088260-60098466	BCCMCs	MAMCs	OK	556.297	109.784	-2.34119	-1.69568	5.00E-05	0.005242	yes
MS4A2	ENSG00000149534.8	chr11:60088260-60098466	BCCMCs	HSMCs	OK	556.297	289.105	-0.944261	-0.712361	0.0102	0.136692	no
MS4A2	ENSG00000149534.8	chr11:60088260-60098466	HSMCs	MAMCs	OK	289.105	109.784	-1.39693	-1.0488	0.00835	0.123076	no

NRAS	ENSG00000213281.4	chr1:114704468-114716894	HSMCs	MAMCs	OK	8.72256	7.36296	-0.244467	-0.175721	0.65465	0.775252	no
NRAS	ENSG00000213281.4	chr1:114704468-114716894	BCCMCs	MAMCs	OK	7.17413	7.36296	0.0374808	0.0270922	0.9412	0.965141	no
NRAS	ENSG00000213281.4	chr1:114704468-114716894	BCCMCs	HSMCs	OK	7.17413	8.72256	0.281947	0.212357	0.50065	0.658876	no
PDK1	ENSG00000152256.13	chr2:172427353-172608669	HSMCs	MAMCs	OK	10.208	14.867	0.542411	0.187037	0.6021	0.737229	no
PDK1	ENSG00000152256.13	chr2:172427353-172608669	BCCMCs	HSMCs	OK	5.18519	10.208	0.977234	0.324112	0.1007	0.374967	no
PDK1	ENSG00000152256.13	chr2:172427353-172608669	BCCMCs	MAMCs	OK	5.18519	14.867	1.51965	0.585044	0.14825	0.434571	no
PIK3CA	ENSG00000121879.3	chr3:179148113-179267002	BCCMCs	HSMCs	OK	15.4213	13.3538	-0.207673	-0.0660449	0.59695	0.733299	no
PIK3CA	ENSG00000121879.3	chr3:179148113-179267002	HSMCs	MAMCs	OK	13.3538	7.04008	-0.923589	-0.372064	0.11315	0.392015	no
PIK3CA	ENSG00000121879.3	chr3:179148113-179267002	BCCMCs	MAMCs	OK	15.4213	7.04008	-1.13126	-0.459184	0.04195	0.261736	no
PIK3R1	ENSG00000145675.14	chr5:68215719-68301821	HSMCs	MAMCs	OK	44.8907	41.3594	-0.118204	-0.0590763	0.8478	0.906888	no
PIK3R1	ENSG00000145675.14	chr5:68215719-68301821	BCCMCs	HSMCs	OK	40.904	44.8907	0.134177	0.0726177	0.71245	0.816428	no
PIK3R1	ENSG00000145675.14	chr5:68215719-68301821	BCCMCs	MAMCs	OK	40.904	41.3594	0.0159733	0.00810648	0.9795	0.987476	no
PLA2G4A	ENSG00000116711.9	chr1:186828952-186988981	BCCMCs	HSMCs	OK	11.2813	12.6502	0.165231	0.115331	0.6772	0.791261	no
PLA2G4A	ENSG00000116711.9	chr1:186828952-186988981	BCCMCs	MAMCs	OK	11.2813	1.76896	-2.67295	-1.72849	0.00405	0.0855463	no
PLA2G4A	ENSG00000116711.9	chr1:186828952-186988981	HSMCs	MAMCs	OK	12.6502	1.76896	-2.83819	-1.75677	0.00405	0.0855463	no
PLCG1	ENSG00000124181.14	chr20:41028817-41317672	HSMCs	MAMCs	OK	2.37524	13.6704	2.52491	0.412267	0.0096	0.132101	no
PLCG1	ENSG00000124181.14	chr20:41028817-41317672	BCCMCs	MAMCs	OK	3.02803	13.6704	2.1746	0.386655	0.0121	0.148763	no
PLCG1	ENSG00000124181.14	chr20:41028817-41317672	BCCMCs	HSMCs	OK	3.02803	2.37524	-0.350306	-0.053356	0.49385	0.653573	no
PRKCA	ENSG00000154229.11	chr17:66302635-66810743	HSMCs	MAMCs	OK	6.78464	31.3916	2.21004	0.69506	0.02345	0.203839	no
PRKCA	ENSG00000154229.11	chr17:66302635-66810743	BCCMCs	HSMCs	OK	19.776	6.78464	-1.5434	-0.442419	0.019	0.183374	no
PRKCA	ENSG00000154229.11	chr17:66302635-66810743	BCCMCs	MAMCs	OK	19.776	31.3916	0.666631	0.224767	0.4679	0.634836	no
RAC1	ENSG00000136238.17	chr7:6374522-6403977	BCCMCs	MAMCs	OK	232	124.256	-0.900806	-0.675967	0.13175	0.416201	no
RAC1	ENSG00000136238.17	chr7:6374522-6403977	BCCMCs	HSMCs	OK	232	266.564	0.200357	0.163063	0.67075	0.786719	no
RAC1	ENSG00000136238.17	chr7:6374522-6403977	HSMCs	MAMCs	OK	266.564	124.256	-1.10116	-0.80855	0.08795	0.354953	no
RAF1	ENSG00000132155.11	chr3:12484431-12664226	BCCMCs	MAMCs	OK	29.2416	10.4475	-1.48487	-0.755526	0.0167	0.17389	no
RAF1	ENSG00000132155.11	chr3:12484431-12664226	HSMCs	MAMCs	OK	33.7003	10.4475	-1.68961	-0.810068	0.0189	0.18282	no
RAF1	ENSG00000132155.11	chr3:12484431-12664226	BCCMCs	HSMCs	OK	29.2416	33.7003	0.20474	0.110943	0.64295	0.766545	no
SOS1	ENSG00000115904.12	chr2:38981395-39124345	HSMCs	MAMCs	OK	9.58347	15.9446	0.734447	0.265321	0.4029	0.589147	no
SOS1	ENSG00000115904.12	chr2:38981395-39124345	BCCMCs	HSMCs	OK	7.56485	9.58347	0.341238	0.102187	0.58195	0.722044	no
SOS1	ENSG00000115904.12	chr2:38981395-39124345	BCCMCs	MAMCs	OK	7.56485	15.9446	1.07568	0.372047	0.177	0.461822	no
SYK	ENSG00000165025.14	chr9:90801786-90898549	BCCMCs	MAMCs	OK	14.9125	3.56064	-2.06631	-1.15515	5.00E-04	0.025397	yes
SYK	ENSG00000165025.14	chr9:90801786-90898549	HSMCs	MAMCs	OK	12.4929	3.56064	-1.81091	-0.863246	0.0013	0.045201	yes
SYK	ENSG00000165025.14	chr9:90801786-90898549	BCCMCs	HSMCs	OK	14.9125	12.4929	-0.255407	-0.10833	0.5567	0.702167	no
TNF	ENSG00000232810.3	chr6:31575566-31578336	HSMCs	MAMCs	OK	2.81278	0.066042	-5.41247	-1.48396	0.29585	0.527421	no
TNF	ENSG00000232810.3	chr6:31575566-31578336	BCCMCs	HSMCs	OK	5.68779	2.81278	-1.01587	-0.519996	0.3541	0.558921	no
TNF	ENSG00000232810.3	chr6:31575566-31578336	BCCMCs	MAMCs	OK	5.68779	0.066042	-6.42834	-1.80051	0.07385	0.330516	no
VAV3	ENSG00000134215.15	chr1:107571159-107994607	BCCMCs	HSMCs	OK	24.0018	10.7106	-1.16411	-0.740748	0.00665	0.10973	no
VAV3	ENSG00000134215.15	chr1:107571159-107994607	HSMCs	MAMCs	OK	10.7106	6.48463	-0.723939	-0.412204	0.2485	0.505621	no
VAV3	ENSG00000134215.15	chr1:107571159-107994607	BCCMCs	MAMCs	OK	24.0018	6.48463	-1.88805	-1.15784	0.0021	0.0593131	no

HSMCs: healthy skin-associated mast cells, BCCMCs: basal cell carcinoma-associated mast cells, MAMCs: melanoma-associated mast cells. FPKM: fragments per kilo base of transcript per million mapped fragments.

Supplementary Table 6: Gene expression of FcεRI signalling-associated molecules in skin CD45$^+$ cells

gene	gene_id	locus	sample_1	sample_2	status	value_1 (FPKM)	value_2 (FPKM)	log2(fold_change)	test_stat	p_value	q_value	significant
AKT1	ENSG00000142208.15	chr14:104769348-104795751	Healthy_CD45	MEL_CD45	OK	30.6817	20.5945	-0.575122	-0.20691	0.2917	0.56316	no
AKT1	ENSG00000142208.15	chr14:104769348-104795751	BCC_CD45	Healthy_CD45	OK	16.4713	30.6817	0.897428	0.288158	0.15465	0.436899	no
AKT1	ENSG00000142208.15	chr14:104769348-104795751	BCC_CD45	MEL_CD45	NOTEST	16.4713	20.5945	0.322306	0	1	1	no
BTK	ENSG00000010671.15	chrX:101349446-101390796	Healthy_CD45	MEL_CD45	NOTEST	5.12893	10.0835	0.975266	0	1	1	no
BTK	ENSG00000010671.15	chrX:101349446-101390796	BCC_CD45	MEL_CD45	NOTEST	3.33027	10.0835	1.59828	0	1	1	no
BTK	ENSG00000010671.15	chrX:101349446-101390796	BCC_CD45	Healthy_CD45	NOTEST	3.33027	5.12893	0.623018	0	1	1	no
CSF2	ENSG00000164400.5	chr5:132073789-132076170	BCC_CD45	Healthy_CD45	NOTEST	3.94089	8.00272	1.02197	0	1	1	no
CSF2	ENSG00000164400.5	chr5:132073789-132076170	Healthy_CD45	MEL_CD45	NOTEST	8.00272	3.27745	-1.28792	0	1	1	no
CSF2	ENSG00000164400.5	chr5:132073789-132076170	BCC_CD45	MEL_CD45	NOTEST	3.94089	3.27745	-0.265949	0	1	1	no
FCER1A	ENSG00000179639.10	chr1:159289713-159308224	BCC_CD45	Healthy_CD45	NOTEST	12.103	4.49568	-1.42875	0	1	1	no
FCER1A	ENSG00000179639.10	chr1:159289713-159308224	BCC_CD45	MEL_CD45	NOTEST	12.103	18.8209	0.636971	0	1	1	no
FCER1A	ENSG00000179639.10	chr1:159289713-159308224	Healthy_CD45	MEL_CD45	NOTEST	4.49568	18.8209	2.06573	0	1	1	no
FCER1G	ENSG00000158869.10	chr1:161215233-161220699	Healthy_CD45	MEL_CD45	OK	211.533	485.801	1.19948	0.562855	0.3513	0.606125	no
FCER1G	ENSG00000158869.10	chr1:161215233-161220699	BCC_CD45	MEL_CD45	OK	100.179	485.801	2.27779	1.12263	0.06795	0.308603	no
FCER1G	ENSG00000158869.10	chr1:161215233-161220699	BCC_CD45	Healthy_CD45	OK	100.179	211.533	1.07831	0.607304	0.41635	0.646271	no
FYN	ENSG00000010810.17	chr6:111660331-111873452	BCC_CD45	Healthy_CD45	OK	148.013	81.2027	-0.866123	-0.402622	0.2837	0.557466	no
FYN	ENSG00000010810.17	chr6:111660331-111873452	Healthy_CD45	MEL_CD45	OK	81.2027	375.862	2.2106	0.600655	0.0021	0.056333	no
FYN	ENSG00000010810.17	chr6:111660331-111873452	BCC_CD45	MEL_CD45	OK	148.013	375.862	1.34448	0.373397	0.11975	0.392289	no
GAB2	ENSG00000033327.12	chr11:78215296-78574874	BCC_CD45	MEL_CD45	NOTEST	15.1578	9.68951	-0.645566	0	1	1	no
GAB2	ENSG00000033327.12	chr11:78215296-78574874	BCC_CD45	Healthy_CD45	NOTEST	15.1578	14	-0.114633	0	1	1	no
GAB2	ENSG00000033327.12	chr11:78215296-78574874	Healthy_CD45	MEL_CD45	NOTEST	14	9.68951	-0.530932	0	1	1	no
GRB2	ENSG00000177885.13	chr17:75318075-75405709	BCC_CD45	MEL_CD45	OK	31.723	63.5397	1.00213	0.32835	0.2033	0.490005	no
GRB2	ENSG00000177885.13	chr17:75318075-75405709	Healthy_CD45	MEL_CD45	OK	39.2904	63.5397	0.693484	0.257881	0.30245	0.571769	no
GRB2	ENSG00000177885.13	chr17:75318075-75405709	BCC_CD45	Healthy_CD45	OK	31.723	39.2904	0.308647	0.113742	0.6441	0.791197	no
HRAS	ENSG00000174775.16	chr11:532241-537287	BCC_CD45	MEL_CD45	NOTEST	0	2.24874		0	1	1	no
HRAS	ENSG00000174775.16	chr11:532241-537287	BCC_CD45	Healthy_CD45	NOTEST	0	4.40341		0	1	1	no
HRAS	ENSG00000174775.16	chr11:532241-537287	Healthy_CD45	MEL_CD45	NOTEST	4.40341	2.24874	-0.969507	0	1	1	no
IL13	ENSG00000169194.9	chr5:132410635-132664272	BCC_CD45	MEL_CD45	NOTEST	5.5036	2.67357	-1.04161	0	1	1	no
IL13	ENSG00000169194.9	chr5:132410635-132664272	Healthy_CD45	MEL_CD45	NOTEST	12.8099	2.67357	-2.26042	0	1	1	no
IL13	ENSG00000169194.9	chr5:132410635-132664272	BCC_CD45	Healthy_CD45	NOTEST	5.5036	12.8099	1.21881	0	1	1	no
IL3	ENSG00000164399.4	chr5:132060528-132063204	Healthy_CD45	MEL_CD45	NOTEST	4.48347	1.90021	-1.23845	0	1	1	no
IL3	ENSG00000164399.4	chr5:132060528-132063204	BCC_CD45	Healthy_CD45	NOTEST	2.65972	4.48347	0.753341	0	1	1	no
IL3	ENSG00000164399.4	chr5:132060528-132063204	BCC_CD45	MEL_CD45	NOTEST	2.65972	1.90021	-0.485112	0	1	1	no
IL4	ENSG00000113520.10	chr5:132673985-132682676	BCC_CD45	Healthy_CD45	NOTEST	0	0	0	0	1	1	no
IL4	ENSG00000113520.10	chr5:132673985-132682676	Healthy_CD45	MEL_CD45	NOTEST	0	3.94206		0	1	1	no
IL4	ENSG00000113520.10	chr5:132673985-132682676	BCC_CD45	MEL_CD45	NOTEST	0	3.94206		0	1	1	no
IL5	ENSG00000113525.9	chr5:132410635-132664272	BCC_CD45	MEL_CD45	NOTEST	9.14181	4.57987	-0.997174	0	1	1	no
IL5	ENSG00000113525.9	chr5:132410635-132664272	Healthy_CD45	MEL_CD45	NOTEST	5.38691	4.57987	-0.234153	0	1	1	no
IL5	ENSG00000113525.9	chr5:132410635-132664272	BCC_CD45	Healthy_CD45	NOTEST	9.14181	5.38691	-0.763021	0	1	1	no

INPP5D	ENSG00000168918.13	chr2:233059966-233207903	BCC_CD45	MEL_CD45	NOTEST	0	2.40302		0	1	1	no
INPP5D	ENSG00000168918.13	chr2:233059966-233207903	BCC_CD45	Healthy_CD45	NOTEST	0	1.44111		0	1	1	no
INPP5D	ENSG00000168918.13	chr2:233059966-233207903	Healthy_CD45	MEL_CD45	NOTEST	1.44111	2.40302	0.73767	0	1	1	no
KRAS	ENSG00000133703.11	chr12:25195215-25250936	BCC_CD45	Healthy_CD45	NOTEST	7.58354	16.6164	1.13166	0	1	1	no
KRAS	ENSG00000133703.11	chr12:25195215-25250936	Healthy_CD45	MEL_CD45	OK	16.6164	39.9726	1.2664	0.447048	0.18275	0.468229	no
KRAS	ENSG00000133703.11	chr12:25195215-25250936	BCC_CD45	MEL_CD45	OK	7.58354	39.9726	2.39807	0.644101	0.05115	0.271967	no
LAT	ENSG00000213658.11	chr16:28973961-28990783	BCC_CD45	Healthy_CD45	OK	34.3661	26.0174	-0.40151	-0.141779	0.4672	0.678622	no
LAT	ENSG00000213658.11	chr16:28973961-28990783	BCC_CD45	MEL_CD45	OK	34.3661	70.7497	1.04174	0.412579	0.08675	0.340089	no
LAT	ENSG00000213658.11	chr16:28973961-28990783	Healthy_CD45	MEL_CD45	OK	26.0174	70.7497	1.44325	0.554442	0.00775	0.110197	no
LCP2	ENSG00000043462.11	chr5:170232446-170298227	BCC_CD45	Healthy_CD45	OK	28.8352	38.3767	0.412402	0.168295	0.62455	0.779452	no
LCP2	ENSG00000043462.11	chr5:170232446-170298227	Healthy_CD45	MEL_CD45	OK	38.3767	48.785	0.346206	0.164051	0.65215	0.796904	no
LCP2	ENSG00000043462.11	chr5:170232446-170298227	BCC_CD45	MEL_CD45	OK	28.8352	48.785	0.758607	0.337735	0.3618	0.613509	no
LYN	ENSG00000254087.7	chr8:55879812-56014168	Healthy_CD45	MEL_CD45	NOTEST	7.57458	9.05492	0.257537	0	1	1	no
LYN	ENSG00000254087.7	chr8:55879812-56014168	BCC_CD45	Healthy_CD45	NOTEST	20.2597	7.57458	-1.41937	0	1	1	no
LYN	ENSG00000254087.7	chr8:55879812-56014168	BCC_CD45	MEL_CD45	NOTEST	20.2597	9.05492	-1.16184	0	1	1	no
MAP2K1	ENSG00000169032.9	chr15:66386816-66497813	BCC_CD45	MEL_CD45	OK	5.52838	29.0778	2.39499	0.96844	0.0558	0.282869	no
MAP2K1	ENSG00000169032.9	chr15:66386816-66497813	Healthy_CD45	MEL_CD45	OK	13.6246	29.0778	1.0937	0.400777	0.398	0.635906	no
MAP2K1	ENSG00000169032.9	chr15:66386816-66497813	BCC_CD45	Healthy_CD45	NOTEST	5.52838	13.6246	1.30129	0	1	1	no
MAP2K2	ENSG00000126934.13	chr19:4090320-4124129	BCC_CD45	MEL_CD45	OK	34.4371	30.0623	-0.19601	-0.0961626	0.7301	0.84279	no
MAP2K2	ENSG00000126934.13	chr19:4090320-4124129	Healthy_CD45	MEL_CD45	OK	32.887	30.0623	-0.129564	-0.0592384	0.8186	0.893842	no
MAP2K2	ENSG00000126934.13	chr19:4090320-4124129	BCC_CD45	Healthy_CD45	OK	34.4371	32.887	-0.0664457	-0.0316598	0.9163	0.951354	no
MAP2K3	ENSG00000034152.18	chr17:21284671-21315240	BCC_CD45	Healthy_CD45	OK	20.275	39.6437	0.967391	0.399053	0.1344	0.412964	no
MAP2K3	ENSG00000034152.18	chr17:21284671-21315240	Healthy_CD45	MEL_CD45	OK	39.6437	46.9927	0.245343	0.10021	0.68335	0.815048	no
MAP2K3	ENSG00000034152.18	chr17:21284671-21315240	BCC_CD45	MEL_CD45	OK	20.275	46.9927	1.21273	0.498146	0.045	0.256005	no
MAP2K4	ENSG00000065559.14	chr17:11998352-12143830	BCC_CD45	Healthy_CD45	OK	26.9306	16.3965	-0.715862	-0.213834	0.38835	0.629998	no
MAP2K4	ENSG00000065559.14	chr17:11998352-12143830	Healthy_CD45	MEL_CD45	OK	16.3965	24.9733	0.607001	0.190131	0.303	0.572015	no
MAP2K4	ENSG00000065559.14	chr17:11998352-12143830	BCC_CD45	MEL_CD45	OK	26.9306	24.9733	-0.108862	-0.0381455	0.8675	0.922761	no
MAP2K6	ENSG00000108984.13	chr17:69414697-69543331	Healthy_CD45	MEL_CD45	OK	26.7704	18.2629	-0.551723	-0.203817	0.48635	0.691584	no
MAP2K6	ENSG00000108984.13	chr17:69414697-69543331	BCC_CD45	Healthy_CD45	OK	40.7324	26.7704	-0.605537	-0.262239	0.5018	0.702062	no
MAP2K6	ENSG00000108984.13	chr17:69414697-69543331	BCC_CD45	MEL_CD45	OK	40.7324	18.2629	-1.15726	-0.491847	0.1188	0.390723	no
MAP2K7	ENSG00000076984.17	chr19:7903842-7914478	Healthy_CD45	MEL_CD45	OK	27.7432	16.5871	-0.742074	-0.276864	0.31635	0.581216	no
MAP2K7	ENSG00000076984.17	chr19:7903842-7914478	BCC_CD45	Healthy_CD45	OK	52.3363	27.7432	-0.915677	-0.408138	0.2475	0.530403	no
MAP2K7	ENSG00000076984.17	chr19:7903842-7914478	BCC_CD45	MEL_CD45	OK	52.3363	16.5871	-1.65775	-0.68654	0.05895	0.289283	no
MAPK1	ENSG00000100030.14	chr22:21754499-21867680	Healthy_CD45	MEL_CD45	OK	27.8277	13.8627	-1.00531	-0.234284	0.3238	0.586809	no
MAPK1	ENSG00000100030.14	chr22:21754499-21867680	BCC_CD45	Healthy_CD45	OK	10.6443	27.8277	1.38643	0.287511	0.2072	0.494417	no
MAPK1	ENSG00000100030.14	chr22:21754499-21867680	BCC_CD45	MEL_CD45	NOTEST	10.6443	13.8627	0.38112	0	1	1	no
MAPK14	ENSG00000112062.10	chr6:36027676-36111236	Healthy_CD45	MEL_CD45	NOTEST	10.6262	10.9127	0.0383843	0	1	1	no
MAPK14	ENSG00000112062.10	chr6:36027676-36111236	BCC_CD45	Healthy_CD45	NOTEST	7.28728	10.6262	0.544175	0	1	1	no
MAPK14	ENSG00000112062.10	chr6:36027676-36111236	BCC_CD45	MEL_CD45	NOTEST	7.28728	10.9127	0.582559	0	1	1	no
MAPK8	ENSG00000107643.15	chr10:48306638-48439360	BCC_CD45	Healthy_CD45	OK	36.0575	16.3168	-1.14394	-0.375198	0.2022	0.489083	no
MAPK8	ENSG00000107643.15	chr10:48306638-48439360	BCC_CD45	MEL_CD45	OK	36.0575	13.3467	-1.43382	-0.530119	0.0228	0.187588	no
MAPK8	ENSG00000107643.15	chr10:48306638-48439360	Healthy_CD45	MEL_CD45	NOTEST	16.3168	13.3467	-0.289881	0	1	1	no
MS4A2	ENSG00000149534.8	chr11:60088260-60098466	BCC_CD45	MEL_CD45	OK	19.5478	48.8818	1.32229	0.643829	0.11655	0.386228	no

MS4A2	ENSG00000149534.8	chr11:60088260-60098466	BCC_CD45	Healthy_CD45	NOTEST	19.5478	15.8264	-0.304673	0	1	1	no
MS4A2	ENSG00000149534.8	chr11:60088260-60098466	Healthy_CD45	MEL_CD45	OK	15.8264	48.8818	1.62697	0.749893	0.13745	0.416755	no
NRAS	ENSG00000213281.4	chr1:114704468-114716894	Healthy_CD45	MEL_CD45	NOTEST	2.87315	8.39035	1.5461	0	1	1	no
NRAS	ENSG00000213281.4	chr1:114704468-114716894	BCC_CD45	MEL_CD45	NOTEST	11.4818	8.39035	-0.452546	0	1	1	no
NRAS	ENSG00000213281.4	chr1:114704468-114716894	BCC_CD45	Healthy_CD45	NOTEST	11.4818	2.87315	-1.99864	0	1	1	no
PDK1	ENSG00000152256.13	chr2:172427353-172608669	Healthy_CD45	MEL_CD45	NOTEST	8.04056	11.662	0.536452	0	1	1	no
PDK1	ENSG00000152256.13	chr2:172427353-172608669	BCC_CD45	Healthy_CD45	NOTEST	10.2278	8.04056	-0.347126	0	1	1	no
PDK1	ENSG00000152256.13	chr2:172427353-172608669	BCC_CD45	MEL_CD45	NOTEST	10.2278	11.662	0.189326	0	1	1	no
PIK3CA	ENSG00000121879.3	chr3:179148113-179267002	BCC_CD45	Healthy_CD45	NOTEST	9.98581	10.3482	0.0514277	0	1	1	no
PIK3CA	ENSG00000121879.3	chr3:179148113-179267002	Healthy_CD45	MEL_CD45	NOTEST	10.3482	7.6741	-0.43131	0	1	1	no
PIK3CA	ENSG00000121879.3	chr3:179148113-179267002	BCC_CD45	MEL_CD45	NOTEST	9.98581	7.6741	-0.379882	0	1	1	no
PIK3R1	ENSG00000145675.14	chr5:68215719-68301821	Healthy_CD45	MEL_CD45	OK	45.6879	83.1317	0.863585	0.26164	0.2004	0.487444	no
PIK3R1	ENSG00000145675.14	chr5:68215719-68301821	BCC_CD45	Healthy_CD45	OK	14.4035	45.6879	1.66539	0.455272	0.01805	0.166804	no
PIK3R1	ENSG00000145675.14	chr5:68215719-68301821	BCC_CD45	MEL_CD45	OK	14.4035	83.1317	2.52898	0.576259	0.0041	0.0793865	no
PLA2G4A	ENSG00000116711.9	chr1:186828952-186988981	BCC_CD45	Healthy_CD45	NOTEST	15.471	4.5884	-1.7535	0	1	1	no
PLA2G4A	ENSG00000116711.9	chr1:186828952-186988981	BCC_CD45	MEL_CD45	NOTEST	15.471	3.1436	-2.29907	0	1	1	no
PLA2G4A	ENSG00000116711.9	chr1:186828952-186988981	Healthy_CD45	MEL_CD45	NOTEST	4.5884	3.1436	-0.545573	0	1	1	no
PLCG1	ENSG00000124181.14	chr20:41028817-41317672	Healthy_CD45	MEL_CD45	NOTEST	17.9293	10.3018	-0.799431	0	1	1	no
PLCG1	ENSG00000124181.14	chr20:41028817-41317672	BCC_CD45	MEL_CD45	NOTEST	16.0056	10.3018	-0.635686	0	1	1	no
PLCG1	ENSG00000124181.14	chr20:41028817-41317672	BCC_CD45	Healthy_CD45	NOTEST	16.0056	17.9293	0.163745	0	1	1	no
PRKCA	ENSG00000154229.11	chr17:66302635-66810743	Healthy_CD45	MEL_CD45	OK	30.3064	21.7204	-0.480573	-0.148803	0.6139	0.772944	no
PRKCA	ENSG00000154229.11	chr17:66302635-66810743	BCC_CD45	Healthy_CD45	OK	30.6771	30.3064	-0.0175393	-0.00530243	0.98925	0.993192	no
PRKCA	ENSG00000154229.11	chr17:66302635-66810743	BCC_CD45	MEL_CD45	OK	30.6771	21.7204	-0.498113	-0.172259	0.66865	0.806804	no
RAC1	ENSG00000136238.17	chr7:6374522-6403977	BCC_CD45	MEL_CD45	OK	69.5263	155.665	1.16281	0.537085	0.23225	0.517483	no
RAC1	ENSG00000136238.17	chr7:6374522-6403977	BCC_CD45	Healthy_CD45	OK	69.5263	218.91	1.6547	0.816731	0.14795	0.429489	no
RAC1	ENSG00000136238.17	chr7:6374522-6403977	Healthy_CD45	MEL_CD45	OK	218.91	155.665	-0.491891	-0.222266	0.6427	0.790567	no
RAF1	ENSG00000132155.11	chr3:12484431-12664226	BCC_CD45	MEL_CD45	OK	37.8568	76.5011	1.01493	0.371405	0.19675	0.484341	no
RAF1	ENSG00000132155.11	chr3:12484431-12664226	Healthy_CD45	MEL_CD45	OK	62.6781	76.5011	0.287519	0.130271	0.7043	0.827473	no
RAF1	ENSG00000132155.11	chr3:12484431-12664226	BCC_CD45	Healthy_CD45	OK	37.8568	62.6781	0.727411	0.263237	0.35805	0.611125	no
SOS1	ENSG00000115904.12	chr2:38981395-39124345	Healthy_CD45	MEL_CD45	NOTEST	10.8397	22.2488	1.0374	0	1	1	no
SOS1	ENSG00000115904.12	chr2:38981395-39124345	BCC_CD45	Healthy_CD45	NOTEST	5.79054	10.8397	0.904557	0	1	1	no
SOS1	ENSG00000115904.12	chr2:38981395-39124345	BCC_CD45	MEL_CD45	NOTEST	5.79054	22.2488	1.94196	0	1	1	no
SYK	ENSG00000165025.14	chr9:90801786-90898549	BCC_CD45	MEL_CD45	NOTEST	4.20608	4.39622	0.0637875	0	1	1	no
SYK	ENSG00000165025.14	chr9:90801786-90898549	Healthy_CD45	MEL_CD45	NOTEST	5.15599	4.39622	-0.229986	0	1	1	no
SYK	ENSG00000165025.14	chr9:90801786-90898549	BCC_CD45	Healthy_CD45	NOTEST	4.20608	5.15599	0.293774	0	1	1	no
TNF	ENSG00000232810.3	chr6:31575566-31578336	Healthy_CD45	MEL_CD45	NOTEST	0	0	0	0	1	1	no
TNF	ENSG00000232810.3	chr6:31575566-31578336	BCC_CD45	Healthy_CD45	NOTEST	0	0	0	0	1	1	no
TNF	ENSG00000232810.3	chr6:31575566-31578336	BCC_CD45	MEL_CD45	NOTEST	0	0	0	0	1	1	no
VAV3	ENSG00000134215.15	chr1:107571159-107994607	BCC_CD45	Healthy_CD45	NOTEST	10.1284	10.3415	0.0300449	0	1	1	no
VAV3	ENSG00000134215.15	chr1:107571159-107994607	Healthy_CD45	MEL_CD45	NOTEST	10.3415	17.4785	0.757129	0	1	1	no
VAV3	ENSG00000134215.15	chr1:107571159-107994607	BCC_CD45	MEL_CD45	NOTEST	10.1284	17.4785	0.787174	0	1	1	no

Healthy_CD45: healthy skin CD45 cells, BCC_CD45: basal cell carcinoma CD45 cells, MEL_CD45: melanoma CD45 cells. FPKM: fragments per kilo base of transcript per million mapped fragments.

Supplementary Table 7: Gene expression of complement cascade-associated molecules in skin MCs

gene	gene_id	locus	sample_1	sample_2	status	value_1 (FPKM)	value_2 (FPKM)	log2 (fold_change)	test_stat	p_value	q_value	significant
C3AR1	ENSG00000171860.4	chr12:8058301-8066471	BCCMCS	MAMCS	OK	17.1008	10.1017	-0.75946	-0.52892	0.13065	0.41486	no
C3AR1	ENSG00000171860.4	chr12:8058301-8066471	BCCMCS	HSMCs	OK	17.1008	45.8078	1.42153	0.88392	0.00755	0.116738	no
C3AR1	ENSG00000171860.4	chr12:8058301-8066471	HSMCs	MAMCS	OK	45.8078	10.1017	-2.18099	-1.3039	0.00545	0.099502	no
C4BPA	ENSG00000123838.10	chr1:207104261-207144972	HSMCs	MAMCS	NOTEST	0.074752	0		0	1	1	no
C4BPA	ENSG00000123838.10	chr1:207104261-207144972	BCCMCS	MAMCS	NOTEST	0.127715	0		0	1	1	no
C4BPA	ENSG00000123838.10	chr1:207104261-207144972	BCCMCS	HSMCs	NOTEST	0.127715	0.074752	-0.77275	0	1	1	no
C4BPB	ENSG00000123843.12	chr1:207088841-207099993	HSMCs	MAMCS	NOTEST	0.434201	0.685901	0.659638	0	1	1	no
C4BPB	ENSG00000123843.12	chr1:207088841-207099993	BCCMCS	MAMCS	NOTEST	0.363	0.685901	0.918032	0	1	1	no
C4BPB	ENSG00000123843.12	chr1:207088841-207099993	BCCMCS	HSMCs	NOTEST	0.363	0.434201	0.258394	0	1	1	no
C5AR1	ENSG00000197405.7	chr19:47290022-47322066	BCCMCS	MAMCS	OK	44.6946	10.8188	-2.04656	-1.39697	7.00E-04	0.03108	yes
C5AR1	ENSG00000197405.7	chr19:47290022-47322066	HSMCs	MAMCS	OK	21.1634	10.8188	-0.96803	-0.65909	0.0799	0.340889	no
C5AR1	ENSG00000197405.7	chr19:47290022-47322066	BCCMCS	HSMCs	OK	44.6946	21.1634	-1.07853	-0.73934	0.00865	0.125111	no
CD46	ENSG00000117335.19	chr1:207752056-207795513	HSMCs	MAMCS	OK	76.0655	44.7641	-0.7649	-0.42591	0.247	0.504974	no
CD46	ENSG00000117335.19	chr1:207752056-207795513	BCCMCS	HSMCs	OK	89.1561	76.0655	-0.22909	-0.1231	0.61425	0.746168	no
CD46	ENSG00000117335.19	chr1:207752056-207795513	BCCMCS	MAMCS	OK	89.1561	44.7641	-0.99399	-0.57385	0.1093	0.38756	no
CD55	ENSG00000196352.14	chr1:207321507-207386804	BCCMCS	MAMCS	OK	126.068	39.1871	-1.68575	-1.40896	0.00495	0.094435	no
CD55	ENSG00000196352.14	chr1:207321507-207386804	HSMCs	MAMCS	OK	226.746	39.1871	-2.53263	-1.86475	4.00E-04	0.022061	yes
CD55	ENSG00000196352.14	chr1:207321507-207386804	BCCMCS	HSMCs	OK	126.068	226.746	0.84688	0.675614	0.0492	0.279232	no
CD59	ENSG00000085063.14	chr11:33698260-33736445	BCCMCS	HSMCs	OK	122.99	322.338	1.39003	1.25425	0.00015	0.011552	yes
CD59	ENSG00000085063.14	chr11:33698260-33736445	HSMCs	MAMCS	OK	322.338	149.175	-1.11157	-0.70744	0.0445	0.2687	no
CD59	ENSG00000085063.14	chr11:33698260-33736445	BCCMCS	MAMCS	OK	122.99	149.175	0.278465	0.17869	0.5675	0.710473	no
CD93	ENSG00000125810.9	chr20:23079348-23086340	BCCMCS	HSMCs	NOTEST	0.418323	0.490736	0.230331	0	1	1	no
CD93	ENSG00000125810.9	chr20:23079348-23086340	HSMCs	MAMCS	OK	0.490736	1.86013	1.92238	0.932748	0.2547	0.508554	no
CD93	ENSG00000125810.9	chr20:23079348-23086340	BCCMCS	MAMCS	OK	0.418323	1.86013	2.15272	1.00909	0.3314	0.545895	no
CFH	ENSG00000000971.15	chr1:196651877-196747504	BCCMCS	MAMCS	OK	1.63254	37.0861	4.50569	1.69188	0.0027	0.068521	no
CFH	ENSG00000000971.15	chr1:196651877-196747504	BCCMCS	HSMCs	OK	1.63254	2.4582	0.590487	0.206006	0.4789	0.642623	no
CFH	ENSG00000000971.15	chr1:196651877-196747504	HSMCs	MAMCS	OK	2.4582	37.0861	3.91521	1.65125	0.0086	0.124729	no
CFHR1	ENSG00000244414.6	chr1:196819744-196959226	HSMCs	MAMCS	OK	0.35198	2.38011	2.75746	0.701225	0.073	0.328981	no
CFHR1	ENSG00000244414.6	chr1:196819744-196959226	BCCMCS	MAMCS	OK	0.164095	2.38011	3.85842	0.716994	0.07775	0.33726	no
CFHR1	ENSG00000244414.6	chr1:196819744-196959226	BCCMCS	HSMCs	NOTEST	0.164095	0.35198	1.10096	0	1	1	no
CFHR2	ENSG00000080910.11	chr1:196819744-196959226	BCCMCS	MAMCS	OK	0.062272	8.27524	7.05408	0.537872	0.0323	0.233909	no
CFHR2	ENSG00000080910.11	chr1:196819744-196959226	BCCMCS	HSMCs	NOTEST	0.062272	0.033375	-0.89982	0	1	1	no
CFHR2	ENSG00000080910.11	chr1:196819744-196959226	HSMCs	MAMCS	OK	0.033375	8.27524	7.9539	0.335375	0.0015	0.049242	yes
CFHR3	ENSG00000116785.13	chr1:196774794-196795406	HSMCs	MAMCS	NOTEST	0.255134	0.215964	-0.24046	0	1	1	no
CFHR3	ENSG00000116785.13	chr1:196774794-196795406	BCCMCS	MAMCS	NOTEST	0.231544	0.215964	-0.1005	0	1	1	no
CFHR3	ENSG00000116785.13	chr1:196774794-196795406	BCCMCS	HSMCs	NOTEST	0.231544	0.255134	0.139968	0	1	1	no
CFHR4	ENSG00000134365.12	chr1:196819744-196959226	BCCMCS	MAMCS	NOTEST	0.043222	0.765895	4.1473	0	1	1	no
CFHR4	ENSG00000134365.12	chr1:196819744-196959226	BCCMCS	HSMCs	NOTEST	0.043222	0.117506	1.44288	0	1	1	no
CFHR4	ENSG00000134365.12	chr1:196819744-196959226	HSMCs	MAMCS	NOTEST	0.117506	0.765895	2.70442	0	1	1	no

CFHR5	ENSG00000134389.9	chr1:196977555-197009674	BCCMCS	HSMCs	NOTEST	0.08277	0.155034	0.905415	0	1	1	no
CFHR5	ENSG00000134389.9	chr1:196977555-197009674	HSMCs	MAMCS	NOTEST	0.155034	0.449522	1.53581	0	1	1	no
CFHR5	ENSG00000134389.9	chr1:196977555-197009674	BCCMCS	MAMCS	NOTEST	0.08277	0.449522	2.44122	0	1	1	no
CFI	ENSG00000205403.12	chr4:109740693-109802179	HSMCs	MAMCS	OK	1.12253	5.57288	2.31167	0.786863	0.0393	0.254987	no
CFI	ENSG00000205403.12	chr4:109740693-109802179	BCCMCS	MAMCS	OK	0.50854	5.57288	3.45399	1.10916	0.0049	0.093976	no
CFI	ENSG00000205403.12	chr4:109740693-109802179	BCCMCS	HSMCs	NOTEST	0.50854	1.12253	1.14232	0	1	1	no
CLU	ENSG00000120885.21	chr8:27596916-27615031	BCCMCS	HSMCs	OK	1513.78	377.903	-2.00207	-1.43589	5.00E-05	0.005242	yes
CLU	ENSG00000120885.21	chr8:27596916-27615031	HSMCs	MAMCS	OK	377.903	764.042	1.01564	0.684617	0.1851	0.467171	no
CLU	ENSG00000120885.21	chr8:27596916-27615031	BCCMCS	MAMCS	OK	1513.78	764.042	-0.98643	-0.63752	0.18745	0.468847	no
CR1	ENSG00000203710.10	chr1:207496146-207640647	HSMCs	MAMCS	OK	0.363966	1.26169	1.79348	0.577949	0.13705	0.422465	no
CR1	ENSG00000203710.10	chr1:207496146-207640647	BCCMCS	MAMCS	OK	0.287661	1.26169	2.13291	0.672693	0.06235	0.308276	no
CR1	ENSG00000203710.10	chr1:207496146-207640647	BCCMCS	HSMCs	NOTEST	0.287661	0.363966	0.339433	0	1	1	no
CR2	ENSG00000117322.17	chr1:207454229-207489895	BCCMCS	HSMCs	NOTEST	0.332466	0.50664	0.607752	0	1	1	no
CR2	ENSG00000117322.17	chr1:207454229-207489895	HSMCs	MAMCS	OK	0.50664	1.46356	1.53045	0.345658	0.2524	0.507434	no
CR2	ENSG00000117322.17	chr1:207454229-207489895	BCCMCS	MAMCS	OK	0.332466	1.46356	2.1382	0.431979	0.0872	0.353566	no
CSMD1	ENSG00000183117.18	chr8:2935352-4994972	BCCMCS	MAMCS	OK	0.719697	5.15387	2.84019	0.62126	0.0022	0.060668	no
CSMD1	ENSG00000183117.18	chr8:2935352-4994972	HSMCs	MAMCS	OK	1.47491	5.15387	1.80503	0.44814	0.0541	0.290478	no
CSMD1	ENSG00000183117.18	chr8:2935352-4994972	BCCMCS	HSMCs	OK	0.719697	1.47491	1.03516	0.210847	0.06725	0.317965	no
CSMD2	ENSG00000121904.17	chr1:33513998-34165842	HSMCs	MAMCS	OK	0.658023	4.51178	2.77749	0.893975	0.00455	0.090756	no
CSMD2	ENSG00000121904.17	chr1:33513998-34165842	BCCMCS	MAMCS	OK	0.373196	4.51178	3.59569	1.03598	3.00E-04	0.018455	yes
CSMD2	ENSG00000121904.17	chr1:33513998-34165842	BCCMCS	HSMCs	NOTEST	0.373196	0.658023	0.818206	0	1	1	no
CSMD3	ENSG00000164796.17	chr8:112222927-113437099	BCCMCS	MAMCS	OK	0.42282	1.79882	2.08893	0.590985	0.06125	0.306205	no
CSMD3	ENSG00000164796.17	chr8:112222927-113437099	HSMCs	MAMCS	OK	0.645859	1.79882	1.47776	0.417732	0.2233	0.491848	no
CSMD3	ENSG00000164796.17	chr8:112222927-113437099	BCCMCS	HSMCs	NOTEST	0.42282	0.645859	0.611175	0	1	1	no
ELANE	ENSG00000197561.6	chr19:851013-856247	HSMCs	MAMCS	NOTEST	0	0	0	0	1	1	no
ELANE	ENSG00000197561.6	chr19:851013-856247	BCCMCS	HSMCs	NOTEST	0.0168	0		0	1	1	no
ELANE	ENSG00000197561.6	chr19:851013-856247	BCCMCS	MAMCS	NOTEST	0.0168	0		0	1	1	no
F2	ENSG00000180210.14	chr11:46719179-46739506	BCCMCS	HSMCs	NOTEST	0.355622	0.689592	0.955397	0	1	1	no
F2	ENSG00000180210.14	chr11:46719179-46739506	BCCMCS	MAMCS	OK	0.355622	7.15803	4.33114	1.60249	0.0417	0.2611	no
F2	ENSG00000180210.14	chr11:46719179-46739506	HSMCs	MAMCS	OK	0.689592	7.15803	3.37575	1.22926	0.06925	0.321845	no
ITGAM	ENSG00000169896.16	chr16:31259989-31332892	BCCMCS	MAMCS	OK	19.7429	16.0228	-0.3012	-0.17607	0.5489	0.69603	no
ITGAM	ENSG00000169896.16	chr16:31259989-31332892	HSMCs	MAMCS	OK	10.13	16.0228	0.6615	0.309128	0.2467	0.504874	no
ITGAM	ENSG00000169896.16	chr16:31259989-31332892	BCCMCS	HSMCs	OK	19.7429	10.13	-0.9627	-0.44651	0.0054	0.098957	no
ITGAX	ENSG00000140678.16	chr16:31355133-31382997	HSMCs	MAMCS	OK	116.684	104.243	-0.16265	-0.11185	0.7983	0.874517	no
ITGAX	ENSG00000140678.16	chr16:31355133-31382997	BCCMCS	MAMCS	OK	98.4145	104.243	0.083006	0.050322	0.89225	0.935434	no
ITGAX	ENSG00000140678.16	chr16:31355133-31382997	BCCMCS	HSMCs	OK	98.4145	116.684	0.24566	0.166222	0.5509	0.697461	no
ITGB2	ENSG00000160255.17	chr21:44885952-44931989	BCCMCS	HSMCs	OK	14.3801	3.5596	-2.01429	-1.01365	5.00E-05	0.005242	yes
ITGB2	ENSG00000160255.17	chr21:44885952-44931989	HSMCs	MAMCS	OK	3.5596	50.5211	3.8271	1.67085	5.00E-05	0.005242	yes
ITGB2	ENSG00000160255.17	chr21:44885952-44931989	BCCMCS	MAMCS	OK	14.3801	50.5211	1.81281	0.922001	0.01515	0.165466	no
SERPING1	ENSG00000149131.15	chr11:57597386-57614853	BCCMCS	HSMCs	OK	9.4659	8.72546	-0.11751	-0.06047	0.8455	0.905484	no
SERPING1	ENSG00000149131.15	chr11:57597386-57614853	HSMCs	MAMCS	OK	8.72546	70.0991	3.00609	1.11314	0.0087	0.125334	no
SERPING1	ENSG00000149131.15	chr11:57597386-57614853	BCCMCS	MAMCS	OK	9.4659	70.0991	2.88858	1.10683	0.00765	0.117729	no
VSIG4	ENSG00000155659.14	chrX:66021737-66040125	BCCMCS	HSMCs	OK	0.505067	1.27066	1.33103	0.340769	0.1793	0.463202	no
VSIG4	ENSG00000155659.14	chrX:66021737-66040125	HSMCs	MAMCS	OK	1.27066	2.38822	0.910355	0.304171	0.5691	0.71167	no
VSIG4	ENSG00000155659.14	chrX:66021737-66040125	BCCMCS	MAMCS	OK	0.505067	2.38822	2.24139	0.649241	0.13595	0.421018	no

VTN	ENSG00000109072.13	chr17:28357580-28407197	BCCMCS	MAMCS	OK	0.786835	4.08699	2.37691	0.308785	0.10055	0.374835	no
VTN	ENSG00000109072.13	chr17:28357580-28407197	BCCMCS	HSMCs	NOTEST	0.786835	0.842093	0.097918	0	1	1	no
VTN	ENSG00000109072.13	chr17:28357580-28407197	HSMCs	MAMCS	OK	0.842093	4.08699	2.27899	0.254156	0.12655	0.410251	no
C1QA	ENSG00000173372.16	chr1:22636505-22639608	BCCMCS	MAMCS	OK	3.90975	160.356	5.35806	2.52959	0.0201	0.188849	no
C1QA	ENSG00000173372.16	chr1:22636505-22639608	BCCMCS	HSMCs	OK	3.90975	1.77381	-1.14022	-0.40373	0.3152	0.536847	no
C1QA	ENSG00000173372.16	chr1:22636505-22639608	HSMCs	MAMCS	OK	1.77381	160.356	6.49828	2.80787	0.0047	0.092066	no
C1QB	ENSG00000173369.15	chr1:22652761-22661538	BCCMCS	MAMCS	OK	3.11405	56.8372	4.18997	1.72973	0.0077	0.118023	no
C1QB	ENSG00000173369.15	chr1:22652761-22661538	BCCMCS	HSMCs	OK	3.11405	1.64364	-0.92189	-0.29222	0.3282	0.543968	no
C1QB	ENSG00000173369.15	chr1:22652761-22661538	HSMCs	MAMCS	OK	1.64364	56.8372	5.11187	2.15227	0.0049	0.093976	no
C1QC	ENSG00000159189.11	chr1:22643629-22648110	HSMCs	MAMCS	OK	1.20875	14.0632	3.54034	1.23231	0.3219	0.540566	no
C1QC	ENSG00000159189.11	chr1:22643629-22648110	BCCMCS	MAMCS	OK	1.36794	14.0632	3.36185	1.2989	0.19775	0.476474	no
C1QC	ENSG00000159189.11	chr1:22643629-22648110	BCCMCS	HSMCs	OK	1.36794	1.20875	-0.17849	-0.05102	0.88135	0.928959	no
C1R	ENSG00000159403.15	chr12:7080208-7122501	BCCMCS	MAMCS	OK	4.74436	78.8548	4.05491	1.60707	0.00015	0.011552	yes
C1R	ENSG00000159403.15	chr12:7080208-7122501	HSMCs	MAMCS	OK	7.01736	78.8548	3.4902	1.43532	7.00E-04	0.03108	yes
C1R	ENSG00000159403.15	chr12:7080208-7122501	BCCMCS	HSMCs	OK	4.74436	7.01736	0.564714	0.228495	0.315	0.536679	no
C1S	ENSG00000182326.14	chr12:6970892-7071032	HSMCs	MAMCS	OK	6.11315	96.2577	3.97691	1.18602	1.00E-04	0.008695	yes
C1S	ENSG00000182326.14	chr12:6970892-7071032	BCCMCS	HSMCs	OK	7.36322	6.11315	-0.26842	-0.06644	0.7086	0.814078	no
C1S	ENSG00000182326.14	chr12:6970892-7071032	BCCMCS	MAMCS	OK	7.36322	96.2577	3.70849	1.25894	5.00E-05	0.005242	yes
C2	ENSG00000166278.14	chr6:31897784-31952084	BCCMCS	MAMCS	OK	1.92109	0.462811	-2.05343	-0.50424	0.0871	0.353348	no
C2	ENSG00000166278.14	chr6:31897784-31952084	BCCMCS	HSMCs	OK	1.92109	0.809263	-1.24725	-0.36251	0.0894	0.357616	no
C2	ENSG00000166278.14	chr6:31897784-31952084	HSMCs	MAMCS	NOTEST	0.809263	0.462811	-0.80619	0	1	1	no
C3	ENSG00000125730.16	chr19:6677703-6737603	BCCMCS	HSMCs	OK	2.95419	6.3428	1.10236	0.161138	0.1476	0.4337	no
C3	ENSG00000125730.16	chr19:6677703-6737603	BCCMCS	MAMCS	OK	2.95419	371.868	6.97589	1.29263	5.00E-05	0.005242	yes
C3	ENSG00000125730.16	chr19:6677703-6737603	HSMCs	MAMCS	OK	6.3428	371.868	5.87353	1.25423	5.00E-05	0.005242	yes
C4A	ENSG00000244731.7	chr6:31969809-32003521	BCCMCS	HSMCs	NOTEST	0.049819	0.013873	-1.84447	0	1	1	no
C4A	ENSG00000244731.7	chr6:31969809-32003521	HSMCs	MAMCS	NOTEST	0.013873	0.554518	5.32093	0	1	1	no
C4A	ENSG00000244731.7	chr6:31969809-32003521	BCCMCS	MAMCS	NOTEST	0.049819	0.554518	3.47646	0	1	1	no
C4B	ENSG00000224389.8	chr6:32014761-32036258	BCCMCS	HSMCs	NOTEST	0.014857	0.002363	-2.65271	0	1	1	no
C4B	ENSG00000224389.8	chr6:32014761-32036258	HSMCs	MAMCS	NOTEST	0.002363	0		0	1	1	no
C4B	ENSG00000224389.8	chr6:32014761-32036258	BCCMCS	MAMCS	NOTEST	0.014857	0		0	1	1	no
C5	ENSG00000106804.7	chr9:120952334-121050276	BCCMCS	HSMCs	NOTEST	0.466913	0.467014	0.00031	0	1	1	no
C5	ENSG00000106804.7	chr9:120952334-121050276	BCCMCS	MAMCS	OK	0.466913	1.46632	1.65098	0.472439	0.14195	0.428016	no
C5	ENSG00000106804.7	chr9:120952334-121050276	HSMCs	MAMCS	OK	0.467014	1.46632	1.65067	0.451436	0.1567	0.443299	no
C6	ENSG00000039537.13	chr5:41142233-41261438	BCCMCS	MAMCS	NOTEST	0.253415	0.911945	1.84745	0	1	1	no
C6	ENSG00000039537.13	chr5:41142233-41261438	HSMCs	MAMCS	NOTEST	0.344305	0.911945	1.40526	0	1	1	no
C6	ENSG00000039537.13	chr5:41142233-41261438	BCCMCS	HSMCs	NOTEST	0.253415	0.344305	0.442189	0	1	1	no
C7	ENSG00000112936.18	chr5:40909251-40982939	BCCMCS	MAMCS	OK	0.680329	7.40496	3.44419	1.05225	0.00515	0.096352	no
C7	ENSG00000112936.18	chr5:40909251-40982939	BCCMCS	HSMCs	OK	0.680329	1.98369	1.54388	0.39687	0.0531	0.288122	no
C7	ENSG00000112936.18	chr5:40909251-40982939	HSMCs	MAMCS	OK	1.98369	7.40496	1.9003	0.638552	0.11425	0.393605	no
C8A	ENSG00000157131.10	chr1:56854805-56918221	BCCMCS	HSMCs	NOTEST	0.044021	0.192866	2.13135	0	1	1	no
C8A	ENSG00000157131.10	chr1:56854805-56918221	HSMCs	MAMCS	NOTEST	0.192866	0.477422	1.30767	0	1	1	no
C8A	ENSG00000157131.10	chr1:56854805-56918221	BCCMCS	MAMCS	NOTEST	0.044021	0.477422	3.43902	0	1	1	no
C8B	ENSG00000021852.12	chr1:56929209-58546802	BCCMCS	HSMCs	NOTEST	0.179542	0.204045	0.184569	0	1	1	no
C8B	ENSG00000021852.12	chr1:56929209-58546802	BCCMCS	MAMCS	NOTEST	0.179542	0.1601	-0.16535	0	1	1	no
C8B	ENSG00000021852.12	chr1:56929209-58546802	HSMCs	MAMCS	NOTEST	0.204045	0.1601	-0.34992	0	1	1	no

C8G	ENSG00000176919.11	chr9:136945245-136946974	BCCMCS	HSMCs	OK	29.367	15.5279	-0.91934	-0.52062	0.09945	0.373121	no
C8G	ENSG00000176919.11	chr9:136945245-136946974	BCCMCS	MAMCS	OK	29.367	4.19897	-2.80609	-1.33601	0.0347	0.241621	no
C8G	ENSG00000176919.11	chr9:136945245-136946974	HSMCs	MAMCS	OK	15.5279	4.19897	-1.88675	-0.8443	0.1226	0.404763	no
C9	ENSG00000113600.10	chr5:39284261-39462300	BCCMCS	MAMCS	NOTEST	0.103401	0.39912	1.94858	0	1	1	no
C9	ENSG00000113600.10	chr5:39284261-39462300	HSMCs	MAMCS	NOTEST	0.110223	0.39912	1.85639	0	1	1	no
C9	ENSG00000113600.10	chr5:39284261-39462300	BCCMCS	HSMCs	NOTEST	0.103401	0.110223	0.092186	0	1	1	no
CFB	ENSG00000243649.8	chr6:31897784-31952084	HSMCs	MAMCS	OK	1.49683	1.35247	-0.14631	-0.03937	0.90005	0.940429	no
CFB	ENSG00000243649.8	chr6:31897784-31952084	BCCMCS	MAMCS	OK	0.463606	1.35247	1.54463	0.25765	0.15695	0.443532	no
CFB	ENSG00000243649.8	chr6:31897784-31952084	BCCMCS	HSMCs	OK	0.463606	1.49683	1.69094	0.277488	0.0344	0.240726	no
CFD	ENSG00000197766.7	chr19:859642-863630	BCCMCS	MAMCS	OK	2.46372	1.24352	-0.98641	-0.34986	0.50405	0.661768	no
CFD	ENSG00000197766.7	chr19:859642-863630	HSMCs	MAMCS	OK	5.85901	1.24352	-2.23623	-0.89971	0.30595	0.531819	no
CFD	ENSG00000197766.7	chr19:859642-863630	BCCMCS	HSMCs	OK	2.46372	5.85901	1.24982	0.490148	0.29305	0.526304	no
CFP	ENSG00000126759.13	chrX:47561099-47630305	BCCMCS	HSMCs	OK	1.46554	0.751842	-0.96293	-0.04899	0.399	0.586677	no
CFP	ENSG00000126759.13	chrX:47561099-47630305	HSMCs	MAMCS	OK	0.751842	4.12368	2.45543	0.128964	0.0971	0.369157	no
CFP	ENSG00000126759.13	chrX:47561099-47630305	BCCMCS	MAMCS	OK	1.46554	4.12368	1.4925	0.082416	0.1925	0.473098	no
COLEC10	ENSG00000184374.2	chr8:118995451-119106582	BCCMCS	HSMCs	NOTEST	0.291709	0.181267	-0.68642	0	1	1	no
COLEC10	ENSG00000184374.2	chr8:118995451-119106582	HSMCs	MAMCS	NOTEST	0.181267	1.05357	2.5391	0	1	1	no
COLEC10	ENSG00000184374.2	chr8:118995451-119106582	BCCMCS	MAMCS	NOTEST	0.291709	1.05357	1.85269	0	1	1	no
COLEC11	ENSG00000118004.17	chr2:3594831-3644644	BCCMCS	HSMCs	OK	0.333718	1.60346	2.26449	0.577364	0.0013	0.045201	yes
COLEC11	ENSG00000118004.17	chr2:3594831-3644644	BCCMCS	MAMCS	NOTEST	0.333718	0.820479	1.29784	0	1	1	no
COLEC11	ENSG00000118004.17	chr2:3594831-3644644	HSMCs	MAMCS	OK	1.60346	0.820479	-0.96665	-0.27605	0.30335	0.531024	no
FCN1	ENSG00000085265.10	chr9:134905889-134917963	BCCMCS	HSMCs	NOTEST	1.12528	0.536383	-1.06895	0	1	1	no
FCN1	ENSG00000085265.10	chr9:134905889-134917963	HSMCs	MAMCS	OK	0.536383	6.83393	3.67138	1.34827	0.10685	0.38412	no
FCN1	ENSG00000085265.10	chr9:134905889-134917963	BCCMCS	MAMCS	OK	1.12528	6.83393	2.60243	0.889767	0.14955	0.436241	no
FCN2	ENSG00000160339.15	chr9:134880811-134887520	BCCMCS	MAMCS	NOTEST	0.063167	0.872652	3.78816	0	1	1	no
FCN2	ENSG00000160339.15	chr9:134880811-134887520	BCCMCS	HSMCs	NOTEST	0.063167	0.023128	-1.44955	0	1	1	no
FCN2	ENSG00000160339.15	chr9:134880811-134887520	HSMCs	MAMCS	NOTEST	0.023128	0.872652	5.2377	0	1	1	no
FCN3	ENSG00000142748.12	chr1:27369111-27374824	BCCMCS	HSMCs	NOTEST	0.4707	0.600572	0.351529	0	1	1	no
FCN3	ENSG00000142748.12	chr1:27369111-27374824	HSMCs	MAMCS	OK	0.600572	2.43422	2.01905	0.732833	0.14955	0.436241	no
FCN3	ENSG00000142748.12	chr1:27369111-27374824	BCCMCS	MAMCS	OK	0.4707	2.43422	2.37058	0.890677	0.07765	0.337124	no
MASP1	ENSG00000127241.16	chr3:187217284-187297933	BCCMCS	HSMCs	NOTEST	1.10032	1.01464	-0.11696	0	1	1	no
MASP1	ENSG00000127241.16	chr3:187217284-187297933	HSMCs	MAMCS	OK	1.01464	2.43102	1.26059	0.37454	0.18155	0.464737	no
MASP1	ENSG00000127241.16	chr3:187217284-187297933	BCCMCS	MAMCS	OK	1.10032	2.43102	1.14363	0.384786	0.12665	0.410382	no
MASP2	ENSG00000009724.16	chr1:11012343-11047233	BCCMCS	MAMCS	OK	0.548328	5.92971	3.43485	0.34612	0.0486	0.277913	no
MASP2	ENSG00000009724.16	chr1:11012343-11047233	BCCMCS	HSMCs	NOTEST	0.548328	0.796963	0.539474	0	1	1	no
MASP2	ENSG00000009724.16	chr1:11012343-11047233	HSMCs	MAMCS	OK	0.796963	5.92971	2.89538	0.298857	0.08565	0.350734	no
MBL2	ENSG00000165471.6	chr10:52765379-52771700	BCCMCS	HSMCs	NOTEST	0.178272	0.304736	0.773481	0	1	1	no
MBL2	ENSG00000165471.6	chr10:52765379-52771700	BCCMCS	MAMCS	NOTEST	0.178272	0.364381	1.03137	0	1	1	no
MBL2	ENSG00000165471.6	chr10:52765379-52771700	HSMCs	MAMCS	NOTEST	0.304736	0.364381	0.257889	0	1	1	no

HSMCs: healthy skin-associated mast cells, BCCMCs: basal cell carcinoma-associated mast cells, MAMCs: melanoma-associated mast cells. FPKM: fragments per kilo base of transcript per million mapped fragments.

Supplementary Table 8: Gene expression of complement cascade-associated molecules in skin CD45^{+} cells

gene	gene_id	locus	sample_1	sample_2	status	value_1 (FPKM)	value_2 (FPKM)	log2 (fold_change)	test_stat	p_value	q_value	significant
C3AR1	ENSG00000171860.4	chr12:8058301-8066471	BCC_CD45	MEL_CD45	OK	74.6249	8.51362	-3.13181	-1.37452	0.2575	0.538254	no
C3AR1	ENSG00000171860.4	chr12:8058301-8066471	BCC_CD45	Healthy_CD45	OK	74.6249	6.99642	-3.41497	-1.19979	0.34855	0.604339	no
C3AR1	ENSG00000171860.4	chr12:8058301-8066471	Healthy_CD45	MEL_CD45	NOTEST	6.99642	8.51362	0.283157	0	1	1	no
C4BPA	ENSG00000123838.10	chr1:207104261-207144972	Healthy_CD45	MEL_CD45	NOTEST	1.14155	0.604614	-0.916911	0	1	1	no
C4BPA	ENSG00000123838.10	chr1:207104261-207144972	BCC_CD45	MEL_CD45	NOTEST	0.4766	0.604614	0.343234	0	1	1	no
C4BPA	ENSG00000123838.10	chr1:207104261-207144972	BCC_CD45	Healthy_CD45	NOTEST	0.4766	1.14155	1.26014	0	1	1	no
C4BPB	ENSG00000123843.12	chr1:207088841-207099993	Healthy_CD45	MEL_CD45	NOTEST	8.07456	4.73441	-0.770198	0	1	1	no
C4BPB	ENSG00000123843.12	chr1:207088841-207099993	BCC_CD45	MEL_CD45	NOTEST	13.3236	4.73441	-1.49273	0	1	1	no
C4BPB	ENSG00000123843.12	chr1:207088841-207099993	BCC_CD45	Healthy_CD45	NOTEST	13.3236	8.07456	-0.722533	0	1	1	no
C5AR1	ENSG00000197405.7	chr19:47290022-47322066	BCC_CD45	MEL_CD45	OK	48.4824	26.5402	-0.86928	-0.380513	0.4369	0.659537	no
C5AR1	ENSG00000197405.7	chr19:47290022-47322066	Healthy_CD45	MEL_CD45	OK	22.8245	26.5402	0.217599	0.062209	0.8287	0.89929	no
C5AR1	ENSG00000197405.7	chr19:47290022-47322066	BCC_CD45	Healthy_CD45	OK	48.4824	22.8245	-1.08688	-0.319073	0.4244	0.650593	no
CD46	ENSG00000117335.19	chr1:207752056-207795513	Healthy_CD45	MEL_CD45	OK	45.1219	75.7261	0.746963	0.259561	0.27075	0.548972	no
CD46	ENSG00000117335.19	chr1:207752056-207795513	BCC_CD45	Healthy_CD45	OK	41.015	45.1219	0.137674	0.062335	0.84175	0.906924	no
CD46	ENSG00000117335.19	chr1:207752056-207795513	BCC_CD45	MEL_CD45	OK	41.015	75.7261	0.884637	0.30147	0.2117	0.499447	no
CD55	ENSG00000196352.14	chr1:207321507-207386804	BCC_CD45	MEL_CD45	OK	33.3603	51.217	0.618489	0.278365	0.5002	0.701212	no
CD55	ENSG00000196352.14	chr1:207321507-207386804	Healthy_CD45	MEL_CD45	OK	24.5639	51.217	1.06009	0.518607	0.2741	0.551651	no
CD55	ENSG00000196352.14	chr1:207321507-207386804	BCC_CD45	Healthy_CD45	OK	33.3603	24.5639	-0.441596	-0.191768	0.65505	0.798805	no
CD59	ENSG00000085063.14	chr11:33698260-33736445	BCC_CD45	Healthy_CD45	OK	32.8596	38.9882	0.246722	0.112449	0.70205	0.826338	no
CD59	ENSG00000085063.14	chr11:33698260-33736445	Healthy_CD45	MEL_CD45	OK	38.9882	92.1068	1.24027	0.593666	0.04035	0.243857	no
CD59	ENSG00000085063.14	chr11:33698260-33736445	BCC_CD45	MEL_CD45	OK	32.8596	92.1068	1.48699	0.710296	0.0327	0.221087	no
CD93	ENSG00000125810.9	chr20:23079348-23086340	BCC_CD45	Healthy_CD45	NOTEST	0.643065	6.986	3.44143	0	1	1	no
CD93	ENSG00000125810.9	chr20:23079348-23086340	Healthy_CD45	MEL_CD45	NOTEST	6.986	6.9941	0.00167149	0	1	1	no
CD93	ENSG00000125810.9	chr20:23079348-23086340	BCC_CD45	MEL_CD45	NOTEST	0.643065	6.9941	3.4431	0	1	1	no
CFH	ENSG00000000971.15	chr1:196651877-196747504	BCC_CD45	MEL_CD45	NOTEST	9.663	2.66608	-1.85775	0	1	1	no
CFH	ENSG00000000971.15	chr1:196651877-196747504	BCC_CD45	Healthy_CD45	NOTEST	9.663	5.75527	-0.747586	0	1	1	no
CFH	ENSG00000000971.15	chr1:196651877-196747504	Healthy_CD45	MEL_CD45	NOTEST	5.75527	2.66608	-1.11016	0	1	1	no
CFHR1	ENSG00000244414.6	chr1:196819744-196959226	Healthy_CD45	MEL_CD45	NOTEST	25.9159	5.75104	-2.17194	0	1	1	no
CFHR1	ENSG00000244414.6	chr1:196819744-196959226	BCC_CD45	MEL_CD45	NOTEST	14.9247	5.75104	-1.37581	0	1	1	no
CFHR1	ENSG00000244414.6	chr1:196819744-196959226	BCC_CD45	Healthy_CD45	NOTEST	14.9247	25.9159	0.796134	0	1	1	no
CFHR2	ENSG00000080910.11	chr1:196819744-196959226	BCC_CD45	MEL_CD45	NOTEST	5.41157	0		0	1	1	no
CFHR2	ENSG00000080910.11	chr1:196819744-196959226	BCC_CD45	Healthy_CD45	NOTEST	5.41157	0		0	1	1	no
CFHR2	ENSG00000080910.11	chr1:196819744-196959226	Healthy_CD45	MEL_CD45	NOTEST	0	0	0	0	1	1	no
CFHR3	ENSG00000116785.13	chr1:196774794-196795406	Healthy_CD45	MEL_CD45	NOTEST	9.66396	2.11495	-2.19199	0	1	1	no
CFHR3	ENSG00000116785.13	chr1:196774794-196795406	BCC_CD45	MEL_CD45	NOTEST	13.0297	2.11495	-2.62311	0	1	1	no
CFHR3	ENSG00000116785.13	chr1:196774794-196795406	BCC_CD45	Healthy_CD45	NOTEST	13.0297	9.66396	-0.431119	0	1	1	no
CFHR4	ENSG00000134365.12	chr1:196819744-196959226	BCC_CD45	MEL_CD45	NOTEST	5.59117	1.55021	-1.85069	0	1	1	no
CFHR4	ENSG00000134365.12	chr1:196819744-196959226	BCC_CD45	Healthy_CD45	NOTEST	5.59117	0.616353	-3.18132	0	1	1	no
CFHR4	ENSG00000134365.12	chr1:196819744-196959226	Healthy_CD45	MEL_CD45	NOTEST	0.616353	1.55021	1.33063	0	1	1	no

CFHR5	ENSG00000134389.9	chr1:196977555-197009674	BCC_CD45	Healthy_CD45	NOTEST	4.89039	0.100718	-5.60155	0	1	1	no
CFHR5	ENSG00000134389.9	chr1:196977555-197009674	Healthy_CD45	MEL_CD45	NOTEST	0.100718	0.32619	1.69539	0	1	1	no
CFHR5	ENSG00000134389.9	chr1:196977555-197009674	BCC_CD45	MEL_CD45	NOTEST	4.89039	0.32619	-3.90616	0	1	1	no
CFI	ENSG00000205403.12	chr4:109740693-109802179	Healthy_CD45	MEL_CD45	NOTEST	9.33975	12.4758	0.417673	0	1	1	no
CFI	ENSG00000205403.12	chr4:109740693-109802179	BCC_CD45	MEL_CD45	OK	36.5139	12.4758	-1.54932	-0.667249	0.0819	0.332434	no
CFI	ENSG00000205403.12	chr4:109740693-109802179	BCC_CD45	Healthy_CD45	OK	36.5139	9.33975	-1.96699	-0.737477	0.0745	0.321078	no
CLU	ENSG00000120885.21	chr8:27596916-27615031	BCC_CD45	Healthy_CD45	OK	24.5629	59.2023	1.26917	0.354612	0.12625	0.400945	no
CLU	ENSG00000120885.21	chr8:27596916-27615031	Healthy_CD45	MEL_CD45	OK	59.2023	82.551	0.479633	0.144911	0.54725	0.7309	no
CLU	ENSG00000120885.21	chr8:27596916-27615031	BCC_CD45	MEL_CD45	OK	24.5629	82.551	1.7488	0.687108	0.00895	0.118805	no
CR1	ENSG00000203710.10	chr1:207496146-207640647	Healthy_CD45	MEL_CD45	NOTEST	3.96201	1.14909	-1.78574	0	1	1	no
CR1	ENSG00000203710.10	chr1:207496146-207640647	BCC_CD45	MEL_CD45	NOTEST	5.55729	1.14909	-2.27389	0	1	1	no
CR1	ENSG00000203710.10	chr1:207496146-207640647	BCC_CD45	Healthy_CD45	NOTEST	5.55729	3.96201	-0.488148	0	1	1	no
CR2	ENSG00000117322.17	chr1:207454229-207489895	BCC_CD45	Healthy_CD45	NOTEST	6.96881	2.11441	-1.72066	0	1	1	no
CR2	ENSG00000117322.17	chr1:207454229-207489895	Healthy_CD45	MEL_CD45	NOTEST	2.11441	0.799597	-1.40291	0	1	1	no
CR2	ENSG00000117322.17	chr1:207454229-207489895	BCC_CD45	MEL_CD45	NOTEST	6.96881	0.799597	-3.12357	0	1	1	no
CSMD1	ENSG00000183117.18	chr8:2935352-4994972	BCC_CD45	MEL_CD45	NOTEST	7.6492	19.4277	1.34473	0	1	1	no
CSMD1	ENSG00000183117.18	chr8:2935352-4994972	Healthy_CD45	MEL_CD45	NOTEST	12.7856	19.4277	0.603599	0	1	1	no
CSMD1	ENSG00000183117.18	chr8:2935352-4994972	BCC_CD45	Healthy_CD45	NOTEST	7.6492	12.7856	0.741135	0	1	1	no
CSMD2	ENSG00000121904.17	chr1:33513998-34165842	Healthy_CD45	MEL_CD45	NOTEST	15.0896	10.7864	-0.484336	0	1	1	no
CSMD2	ENSG00000121904.17	chr1:33513998-34165842	BCC_CD45	MEL_CD45	OK	31.6063	10.7864	-1.55099	-0.652469	0.04925	0.267824	no
CSMD2	ENSG00000121904.17	chr1:33513998-34165842	BCC_CD45	Healthy_CD45	OK	31.6063	15.0896	-1.06666	-0.493506	0.222	0.508241	no
CSMD3	ENSG00000164796.17	chr8:112222927-113437099	BCC_CD45	MEL_CD45	OK	33.6527	12.5361	-1.42463	-0.509359	0.19115	0.478123	no
CSMD3	ENSG00000164796.17	chr8:112222927-113437099	Healthy_CD45	MEL_CD45	NOTEST	18.0345	12.5361	-0.524671	0	1	1	no
CSMD3	ENSG00000164796.17	chr8:112222927-113437099	BCC_CD45	Healthy_CD45	OK	33.6527	18.0345	-0.89996	-0.29212	0.38255	0.626997	no
ELANE	ENSG00000197561.6	chr19:851013-856247	Healthy_CD45	MEL_CD45	NOTEST	0.481018	0		0	1	1	no
ELANE	ENSG00000197561.6	chr19:851013-856247	BCC_CD45	Healthy_CD45	NOTEST	0	0.481018		0	1	1	no
ELANE	ENSG00000197561.6	chr19:851013-856247	BCC_CD45	MEL_CD45	NOTEST	0	0	0	0	1	1	no
F2	ENSG00000180210.14	chr11:46719179-46739506	BCC_CD45	Healthy_CD45	NOTEST	1.04316	8.43549	3.01551	0	1	1	no
F2	ENSG00000180210.14	chr11:46719179-46739506	BCC_CD45	MEL_CD45	NOTEST	1.04316	2.93396	1.49189	0	1	1	no
F2	ENSG00000180210.14	chr11:46719179-46739506	Healthy_CD45	MEL_CD45	NOTEST	8.43549	2.93396	-1.52362	0	1	1	no
ITGAM	ENSG00000169896.16	chr16:31259989-31332892	BCC_CD45	MEL_CD45	NOTEST	19.2634	20.2579	0.072617	0	1	1	no
ITGAM	ENSG00000169896.16	chr16:31259989-31332892	Healthy_CD45	MEL_CD45	OK	46.762	20.2579	-1.20686	-0.415119	0.1842	0.470069	no
ITGAM	ENSG00000169896.16	chr16:31259989-31332892	BCC_CD45	Healthy_CD45	OK	19.2634	46.762	1.27947	0.50072	0.2607	0.5407	no
ITGAX	ENSG00000140678.16	chr16:31355133-31382997	Healthy_CD45	MEL_CD45	OK	13.167	34.8776	1.40538	0.572858	0.10245	0.365081	no
ITGAX	ENSG00000140678.16	chr16:31355133-31382997	BCC_CD45	MEL_CD45	OK	21.9594	34.8776	0.667464	0.287418	0.34455	0.601917	no
ITGAX	ENSG00000140678.16	chr16:31355133-31382997	BCC_CD45	Healthy_CD45	NOTEST	21.9594	13.167	-0.737913	0	1	1	no
ITGB2	ENSG00000160255.17	chr21:44885952-44931989	BCC_CD45	Healthy_CD45	OK	63.2583	9.55927	-2.72628	-0.582359	0.0053	0.0913294	no
ITGB2	ENSG00000160255.17	chr21:44885952-44931989	Healthy_CD45	MEL_CD45	OK	9.55927	43.1935	2.17584	0.448659	0.0044	0.0829096	no
ITGB2	ENSG00000160255.17	chr21:44885952-44931989	BCC_CD45	MEL_CD45	OK	63.2583	43.1935	-0.55044	-0.215707	0.3311	0.592438	no
SERPING1	ENSG00000149131.15	chr11:57597386-57614853	BCC_CD45	Healthy_CD45	OK	3.53959	29.9128	3.07911	0.691518	5.00E-05	0.0046728	yes
SERPING1	ENSG00000149131.15	chr11:57597386-57614853	Healthy_CD45	MEL_CD45	OK	29.9128	23.7182	-0.334769	-0.137041	0.60905	0.769639	no
SERPING1	ENSG00000149131.15	chr11:57597386-57614853	BCC_CD45	MEL_CD45	NOTEST	3.53959	23.7182	2.74434	0	1	1	no
VSIG4	ENSG00000155659.14	chrX:66021737-66040125	BCC_CD45	Healthy_CD45	NOTEST	7.65315	8.15073	0.0908759	0	1	1	no
VSIG4	ENSG00000155659.14	chrX:66021737-66040125	Healthy_CD45	MEL_CD45	NOTEST	8.15073	9.19737	0.174291	0	1	1	no
VSIG4	ENSG00000155659.14	chrX:66021737-66040125	BCC_CD45	MEL_CD45	NOTEST	7.65315	9.19737	0.265167	0	1	1	no

VTN	ENSG00000109072.13	chr17:28357580-28407197	BCC_CD45	MEL_CD45	NOTEST	18.3046	0.469477	-5.28501	0	1	1	no
VTN	ENSG00000109072.13	chr17:28357580-28407197	BCC_CD45	Healthy_CD45	NOTEST	18.3046	2.92641	-2.645	0	1	1	no
VTN	ENSG00000109072.13	chr17:28357580-28407197	Healthy_CD45	MEL_CD45	NOTEST	2.92641	0.469477	-2.64001	0	1	1	no
C1QA	ENSG00000173372.16	chr1:22636505-22639608	BCC_CD45	MEL_CD45	OK	13.4727	479.376	5.15305	1.81254	0.0421	0.248536	no
C1QA	ENSG00000173372.16	chr1:22636505-22639608	BCC_CD45	Healthy_CD45	OK	13.4727	51.4173	1.93222	0.874555	0.3833	0.627339	no
C1QA	ENSG00000173372.16	chr1:22636505-22639608	Healthy_CD45	MEL_CD45	OK	51.4173	479.376	3.22083	1.20223	0.13115	0.408713	no
C1QB	ENSG00000173369.15	chr1:22652761-22661538	BCC_CD45	MEL_CD45	OK	7.52175	37.7815	2.32854	0.833125	0.15065	0.43272	no
C1QB	ENSG00000173369.15	chr1:22652761-22661538	BCC_CD45	Healthy_CD45	NOTEST	7.52175	2.54271	-1.5647	0	1	1	no
C1QB	ENSG00000173369.15	chr1:22652761-22661538	Healthy_CD45	MEL_CD45	OK	2.54271	37.7815	3.89324	0.862291	0.2664	0.5451	no
C1QC	ENSG00000159189.11	chr1:22643629-22648110	Healthy_CD45	MEL_CD45	NOTEST	1.1957	14.9087	3.64024	0	1	1	no
C1QC	ENSG00000159189.11	chr1:22643629-22648110	BCC_CD45	MEL_CD45	NOTEST	0	14.9087		0	1	1	no
C1QC	ENSG00000159189.11	chr1:22643629-22648110	BCC_CD45	Healthy_CD45	NOTEST	0	1.1957		0	1	1	no
C1R	ENSG00000159403.15	chr12:7080208-7122501	BCC_CD45	MEL_CD45	NOTEST	12.9743	13.9995	0.109724	0	1	1	no
C1R	ENSG00000159403.15	chr12:7080208-7122501	Healthy_CD45	MEL_CD45	NOTEST	15.4935	13.9995	-0.146279	0	1	1	no
C1R	ENSG00000159403.15	chr12:7080208-7122501	BCC_CD45	Healthy_CD45	NOTEST	12.9743	15.4935	0.256003	0	1	1	no
C1S	ENSG00000182326.14	chr12:6970892-7071032	Healthy_CD45	MEL_CD45	NOTEST	13.3765	23.0679	0.786187	0	1	1	no
C1S	ENSG00000182326.14	chr12:6970892-7071032	BCC_CD45	Healthy_CD45	OK	25.9817	13.3765	-0.957799	-0.291941	0.09295	0.350635	no
C1S	ENSG00000182326.14	chr12:6970892-7071032	BCC_CD45	MEL_CD45	OK	25.9817	23.0679	-0.171611	-0.052296	0.7345	0.845175	no
C2	ENSG00000166278.14	chr6:31897784-31952084	BCC_CD45	MEL_CD45	NOTEST	0	0.1318		0	1	1	no
C2	ENSG00000166278.14	chr6:31897784-31952084	BCC_CD45	Healthy_CD45	NOTEST	0	0	0	0	1	1	no
C2	ENSG00000166278.14	chr6:31897784-31952084	Healthy_CD45	MEL_CD45	NOTEST	0	0.1318		0	1	1	no
C3	ENSG00000125730.16	chr19:6677703-6737603	BCC_CD45	Healthy_CD45	OK	90.5566	108.719	0.263717	0.126803	0.66135	0.802561	no
C3	ENSG00000125730.16	chr19:6677703-6737603	BCC_CD45	MEL_CD45	OK	90.5566	71.0046	-0.350908	-0.161222	0.54605	0.730114	no
C3	ENSG00000125730.16	chr19:6677703-6737603	Healthy_CD45	MEL_CD45	OK	108.719	71.0046	-0.614624	-0.288839	0.2566	0.53782	no
C4A	ENSG00000244731.7	chr6:31969809-32003521	BCC_CD45	Healthy_CD45	NOTEST	0	0	0	0	1	1	no
C4A	ENSG00000244731.7	chr6:31969809-32003521	Healthy_CD45	MEL_CD45	NOTEST	0	0	0	0	1	1	no
C4A	ENSG00000244731.7	chr6:31969809-32003521	BCC_CD45	MEL_CD45	NOTEST	0	0	0	0	1	1	no
C4B	ENSG00000224389.8	chr6:32014761-32036258	BCC_CD45	Healthy_CD45	NOTEST	0	0	0	0	1	1	no
C4B	ENSG00000224389.8	chr6:32014761-32036258	Healthy_CD45	MEL_CD45	NOTEST	0	0	0	0	1	1	no
C4B	ENSG00000224389.8	chr6:32014761-32036258	BCC_CD45	MEL_CD45	NOTEST	0	0	0	0	1	1	no
C5	ENSG00000106804.7	chr9:120952334-121050276	BCC_CD45	Healthy_CD45	NOTEST	2.76555	2.43799	-0.181877	0	1	1	no
C5	ENSG00000106804.7	chr9:120952334-121050276	BCC_CD45	MEL_CD45	NOTEST	2.76555	8.3006	1.58565	0	1	1	no
C5	ENSG00000106804.7	chr9:120952334-121050276	Healthy_CD45	MEL_CD45	NOTEST	2.43799	8.3006	1.76753	0	1	1	no
C6	ENSG00000039537.13	chr5:41142233-41261438	BCC_CD45	MEL_CD45	NOTEST	0	5.7616		0	1	1	no
C6	ENSG00000039537.13	chr5:41142233-41261438	Healthy_CD45	MEL_CD45	NOTEST	1.86483	5.7616	1.62743	0	1	1	no
C6	ENSG00000039537.13	chr5:41142233-41261438	BCC_CD45	Healthy_CD45	NOTEST	0	1.86483		0	1	1	no
C7	ENSG00000112936.18	chr5:40909251-40982939	BCC_CD45	MEL_CD45	OK	35.4735	115.671	1.70522	0.804198	0.0951	0.354362	no
C7	ENSG00000112936.18	chr5:40909251-40982939	BCC_CD45	Healthy_CD45	OK	35.4735	83.5221	1.23542	0.583905	0.3228	0.586143	no
C7	ENSG00000112936.18	chr5:40909251-40982939	Healthy_CD45	MEL_CD45	OK	83.5221	115.671	0.469802	0.234968	0.70325	0.826994	no
C8A	ENSG00000157131.10	chr1:56854805-56918221	BCC_CD45	Healthy_CD45	NOTEST	5.86717	1.72136	-1.76911	0	1	1	no
C8A	ENSG00000157131.10	chr1:56854805-56918221	Healthy_CD45	MEL_CD45	NOTEST	1.72136	0		0	1	1	no
C8A	ENSG00000157131.10	chr1:56854805-56918221	BCC_CD45	MEL_CD45	NOTEST	5.86717	0		0	1	1	no
C8B	ENSG00000021852.12	chr1:56929209-58546802	BCC_CD45	Healthy_CD45	NOTEST	0.331778	1.68151	2.34147	0	1	1	no
C8B	ENSG00000021852.12	chr1:56929209-58546802	BCC_CD45	MEL_CD45	NOTEST	0.331778	0		0	1	1	no
C8B	ENSG00000021852.12	chr1:56929209-58546802	Healthy_CD45	MEL_CD45	NOTEST	1.68151	0		0	1	1	no

C8G	ENSG00000176919.11	chr9:136945245-136946974	BCC_CD45	Healthy_CD45	NOTEST	26.5412	25.1262	-0.0790429	0	1	1	no
C8G	ENSG00000176919.11	chr9:136945245-136946974	BCC_CD45	MEL_CD45	NOTEST	26.5412	4.111	-2.69067	0	1	1	no
C8G	ENSG00000176919.11	chr9:136945245-136946974	Healthy_CD45	MEL_CD45	NOTEST	25.1262	4.111	-2.61163	0	1	1	no
C9	ENSG00000113600.10	chr5:39284261-39462300	BCC_CD45	MEL_CD45	NOTEST	3.56097	3.58066	0.00795288	0	1	1	no
C9	ENSG00000113600.10	chr5:39284261-39462300	Healthy_CD45	MEL_CD45	NOTEST	0.104481	3.58066	5.09891	0	1	1	no
C9	ENSG00000113600.10	chr5:39284261-39462300	BCC_CD45	Healthy_CD45	NOTEST	3.56097	0.104481	-5.09096	0	1	1	no
CFB	ENSG00000243649.8	chr6:31897784-31952084	Healthy_CD45	MEL_CD45	NOTEST	0	0.135779		0	1	1	no
CFB	ENSG00000243649.8	chr6:31897784-31952084	BCC_CD45	MEL_CD45	NOTEST	0	0.135779		0	1	1	no
CFB	ENSG00000243649.8	chr6:31897784-31952084	BCC_CD45	Healthy_CD45	NOTEST	0	0	0	0	1	1	no
CFD	ENSG00000197766.7	chr19:859642-863630	BCC_CD45	MEL_CD45	NOTEST	1.61243	2.2186	0.460411	0	1	1	no
CFD	ENSG00000197766.7	chr19:859642-863630	Healthy_CD45	MEL_CD45	NOTEST	0	2.2186	Inf	0	1	1	no
CFD	ENSG00000197766.7	chr19:859642-863630	BCC_CD45	Healthy_CD45	NOTEST	1.61243	0		0	1	1	no
CFP	ENSG00000126759.13	chrX:47561099-47630305	BCC_CD45	Healthy_CD45	NOTEST	9.25614	16.9291	0.871019	0	1	1	no
CFP	ENSG00000126759.13	chrX:47561099-47630305	Healthy_CD45	MEL_CD45	NOTEST	16.9291	9.15691	-0.886569	0	1	1	no
CFP	ENSG00000126759.13	chrX:47561099-47630305	BCC_CD45	MEL_CD45	NOTEST	9.25614	9.15691	-0.0155501	0	1	1	no
COLEC10	ENSG00000184374.2	chr8:118995451-119106582	BCC_CD45	Healthy_CD45	NOTEST	0	0	0	0	1	1	no
COLEC10	ENSG00000184374.2	chr8:118995451-119106582	Healthy_CD45	MEL_CD45	NOTEST	0	0	0	0	1	1	no
COLEC10	ENSG00000184374.2	chr8:118995451-119106582	BCC_CD45	MEL_CD45	NOTEST	0	0	0	0	1	1	no
COLEC11	ENSG00000118004.17	chr2:3594831-3644644	BCC_CD45	Healthy_CD45	NOTEST	6.68576	12.2656	0.875453	0	1	1	no
COLEC11	ENSG00000118004.17	chr2:3594831-3644644	BCC_CD45	MEL_CD45	NOTEST	6.68576	0.0396833	-7.39642	0	1	1	no
COLEC11	ENSG00000118004.17	chr2:3594831-3644644	Healthy_CD45	MEL_CD45	NOTEST	12.2656	0.0396833	-8.27187	0	1	1	no
FCN1	ENSG00000085265.10	chr9:134905889-134917963	BCC_CD45	Healthy_CD45	NOTEST	6.9635	5.46206	-0.350369	0	1	1	no
FCN1	ENSG00000085265.10	chr9:134905889-134917963	Healthy_CD45	MEL_CD45	NOTEST	5.46206	22.9099	2.06845	0	1	1	no
FCN1	ENSG00000085265.10	chr9:134905889-134917963	BCC_CD45	MEL_CD45	NOTEST	6.9635	22.9099	1.71809	0	1	1	no
FCN2	ENSG00000160339.15	chr9:134880811-134887520	BCC_CD45	MEL_CD45	NOTEST	4.85235	2.40475	-1.0128	0	1	1	no
FCN2	ENSG00000160339.15	chr9:134880811-134887520	BCC_CD45	Healthy_CD45	NOTEST	4.85235	0		0	1	1	no
FCN2	ENSG00000160339.15	chr9:134880811-134887520	Healthy_CD45	MEL_CD45	NOTEST	0	2.40475		0	1	1	no
FCN3	ENSG00000142748.12	chr1:27369111-27374824	BCC_CD45	Healthy_CD45	OK	8.69963	27.5128	1.66108	0.628684	0.39755	0.635625	no
FCN3	ENSG00000142748.12	chr1:27369111-27374824	Healthy_CD45	MEL_CD45	OK	27.5128	9.54255	-1.52766	-0.654165	0.1862	0.472007	no
FCN3	ENSG00000142748.12	chr1:27369111-27374824	BCC_CD45	MEL_CD45	NOTEST	8.69963	9.54255	0.13342	0	1	1	no
MASP1	ENSG00000127241.16	chr3:187217284-187297933	BCC_CD45	Healthy_CD45	NOTEST	19.7788	10.7211	-0.883506	0	1	1	no
MASP1	ENSG00000127241.16	chr3:187217284-187297933	Healthy_CD45	MEL_CD45	NOTEST	10.7211	7.31519	-0.551486	0	1	1	no
MASP1	ENSG00000127241.16	chr3:187217284-187297933	BCC_CD45	MEL_CD45	NOTEST	19.7788	7.31519	-1.43499	0	1	1	no
MASP2	ENSG00000009724.16	chr1:11012343-11047233	BCC_CD45	MEL_CD45	NOTEST	5.56145	7.54403	0.439872	0	1	1	no
MASP2	ENSG00000009724.16	chr1:11012343-11047233	BCC_CD45	Healthy_CD45	NOTEST	5.56145	17.6425	1.66552	0	1	1	no
MASP2	ENSG00000009724.16	chr1:11012343-11047233	Healthy_CD45	MEL_CD45	NOTEST	17.6425	7.54403	-1.22565	0	1	1	no
MBL2	ENSG00000165471.6	chr10:52765379-52771700	BCC_CD45	Healthy_CD45	NOTEST	7.92042	4.57972	-0.790318	0	1	1	no
MBL2	ENSG00000165471.6	chr10:52765379-52771700	BCC_CD45	MEL_CD45	NOTEST	7.92042	0.905087	-3.12945	0	1	1	no
MBL2	ENSG00000165471.6	chr10:52765379-52771700	Healthy_CD45	MEL_CD45	NOTEST	4.57972	0.905087	-2.33913	0	1	1	no

Healthy_CD45: healthy skin CD45 cells, BCC_CD45: basal cell carcinoma CD45 cells, MEL_CD45: melanoma CD45 cells. FPKM: fragments per kilo base of transcript per million mapped fragments.

Supplementary Table 9: Gene expression of proteases in skin MCs

gene	gene_id	locus	sample_1	sample_2	status	value_1 (FPKM)	value_2 (FPKM)	log2 (fold_change)	test_stat	p_value	q_value	significant
CMA1	ENSG00000092009.10	chr14:24501593-24508688	BCCMCS	HSMCs	OK	1893.29	711.153	-1.41266	-1.24183	5.00E-05	0.005242	yes
CMA1	ENSG00000092009.10	chr14:24501593-24508688	HSMCs	MAMCS	OK	711.153	255.158	-1.47877	-1.20029	0.0051	0.0960177	no
CMA1	ENSG00000092009.10	chr14:24501593-24508688	BCCMCS	MAMCS	OK	1893.29	255.158	-2.89143	-2.30627	5.00E-05	0.005242	yes
CTSG	ENSG00000100448.3	chr14:24573521-24576260	BCCMCS	MAMCS	OK	9646.37	1228.71	-2.97284	-2.26875	5.00E-05	0.005242	yes
CTSG	ENSG00000100448.3	chr14:24573521-24576260	HSMCs	MAMCS	OK	3493.61	1228.71	-1.50757	-1.17744	0.00995	0.134627	no
CTSG	ENSG00000100448.3	chr14:24573521-24576260	BCCMCS	HSMCs	OK	9646.37	3493.61	-1.46527	-1.09622	3.00E-04	0.0184548	yes
MMP14	ENSG00000157227.12	chr14:22836556-22849027	BCCMCS	MAMCS	OK	0.793446	18.9898	4.58095	1.56175	0.00045	0.0237337	yes
MMP14	ENSG00000157227.12	chr14:22836556-22849027	BCCMCS	HSMCs	NOTEST	0.793446	0.887542	0.161683	0	1	1	no
MMP14	ENSG00000157227.12	chr14:22836556-22849027	HSMCs	MAMCS	OK	0.887542	18.9898	4.41927	1.4825	0.00085	0.0349007	yes
MMP17	ENSG00000198598.6	chr12:131828392-131851783	HSMCs	MAMCS	OK	1.40141	15.9252	3.50636	1.03387	0.00095	0.0374849	yes
MMP17	ENSG00000198598.6	chr12:131828392-131851783	BCCMCS	HSMCs	OK	1.77331	1.40141	-0.339564	-0.138408	0.44945	0.622	no
MMP17	ENSG00000198598.6	chr12:131828392-131851783	BCCMCS	MAMCS	OK	1.77331	15.9252	3.16679	0.943278	0.00115	0.0419582	yes
MMP2	ENSG00000087245.12	chr16:55389699-55506691	BCCMCS	HSMCs	OK	4.54302	8.40361	0.887357	0.293698	0.19515	0.474776	no
MMP2	ENSG00000087245.12	chr16:55389699-55506691	BCCMCS	MAMCS	OK	4.54302	85.6196	4.23622	1.66531	0.00365	0.0808987	no
MMP2	ENSG00000087245.12	chr16:55389699-55506691	HSMCs	MAMCS	OK	8.40361	85.6196	3.34886	1.26229	0.01645	0.172593	no
MMP7	ENSG00000137673.8	chr11:102520507-102530753	BCCMCS	HSMCs	NOTEST	0.830326	0.777128	-0.0955268	0	1	1	no
MMP7	ENSG00000137673.8	chr11:102520507-102530753	HSMCs	MAMCS	OK	0.777128	14.2398	4.19563	1.53674	0.10725	0.384874	no
MMP7	ENSG00000137673.8	chr11:102520507-102530753	BCCMCS	MAMCS	OK	0.830326	14.2398	4.10011	1.43616	0.0999	0.373911	no
MMP9	ENSG00000100985.7	chr20:46008907-46060152	BCCMCS	MAMCS	OK	8.08198	33.8991	2.06847	1.19308	0.0631	0.309713	no
MMP9	ENSG00000100985.7	chr20:46008907-46060152	HSMCs	MAMCS	OK	8.24228	33.8991	2.04013	1.17828	0.0472	0.274751	no
MMP9	ENSG00000100985.7	chr20:46008907-46060152	BCCMCS	HSMCs	OK	8.08198	8.24228	0.028333	0.014633	0.97095	0.982565	no
TPSAB1	ENSG00000172236.16	chr16:1240695-1242554	HSMCs	MAMCS	OK	7561.54	2036.4	-1.89266	-1.07712	0.0167	0.17389	no
TPSAB1	ENSG00000172236.16	chr16:1240695-1242554	BCCMCS	MAMCS	OK	3390.41	2036.4	-0.735439	-0.0709526	0.56425	0.708234	no
TPSAB1	ENSG00000172236.16	chr16:1240695-1242554	BCCMCS	HSMCs	OK	3390.41	7561.54	1.15722	0.111856	0.29345	0.526451	no
CPA3	ENSG00000163751.3	chr3:148791101-148960112	BCCMCs	HSMCs	OK	3534.78	861.826	-2.03615	-1.59105	5.00E-05	0.005242	yes
CPA3	ENSG00000163751.3	chr3:148791101-148960112	BCCMCs	MAMCs	OK	3534.78	629.941	-2.48833	-1.80126	5.00E-05	0.005242	yes
CPA3	ENSG00000163751.3	chr3:148791101-148960112	HSMCs	MAMCs	OK	861.826	629.941	-0.45218	-0.36831	0.38965	0.581293	no

HSMCs: healthy skin-associated mast cells, BCCMCs: basal cell carcinoma-associated mast cells, MAMCs: melanoma-associated mast cells. FPKM: fragments per kilo base of transcript per million mapped fragments.

Supplementary materials and methods

Gene Set Enrichment Analysis (GSEA)

Gene Set Enrichment Analysis (Version 6.3, Broad Institute) of complement cascade and Fcε RI signalling GO term gene sets was performed for HSMCs and MAMCs using the Signal2Noise comparison setting.(11).

EdU flow cytometry cell proliferation assay

A375 and RPMI-7951 melanoma cell lines were cultured 48 hours in the presence of MCs at ratio 2:1 (melanoma cells:MCs). As negative control melanoma cells were cultured alone. Click-iT™ Plus EdU Alexa Fluor™ 647 Flow Cytometry Assay Kit (C10634, ThermoFisher) was used for analysing DNA replication in proliferating cells. 10 µM EdU (5-ethynyl-2´-deoxyuridine) was added the last 2 hours in cell cultures. Cells were harvested and a CD45 (2D1, Biolegend) surface flow cytometry staining. Then, after fixation, EdU incorporation in melanoma cells was measured by intracellular flow cytometry staining according to the recommended staining protocol. Cells were resuspended in FACS buffer, and analysed with an LSRII or LSRFortessa flow instrument (BD Biosciences). Data were analysed using the Flowjo software (Treestar Inc.) and melanoma cell proliferation was measured as a percentage of EdU^+ cells in gated $CD45^-$ cells to exclude MCs.

Cell viability and apoptosis

MCs were stained with 1µM Cell Trace Violet proliferation kit (C34571, Thermo Fisher Scientific) in PBS for 10 minutes at 37°C, and washed in complete medium twice. Labelled MCs were added to RPMI-7951 melanoma cell line cultures for 24 hours at ratio 2:1 (melanoma cells:MCs). As negative control melanoma cells were cultured alone.
Cell viability was measured using the live/dead™ blue viability dye and the apoptotic/necrotic markers (AnnexinV). After 24 hours, harvested cells were washed with cold PBS twice and incubated 15 minutes with live/dead™ blue viability dye (LIVE/DEAD™ Fixable Blue Dead Cell Stain Kit, ThermoFisher Scientific). After a wash, with cold Annexin V Binding Buffer (422201, Biolegend), cells were stained with FITC Annexin V (640906, Biolegend) in Annexin V binding buffer for 15 minutes at RT (22°C) in the dark. Then, cells were washed and resuspended in Annexin V Binding Buffer and analysed by flow cytometry (LSRII or LSRFortessa flow instrument, BD Biosciences). Data were analysed using the Flowjo software (Treestar Inc.). Melanoma cells were gated on Cell Trace Violet negative population to exclude MCs and the viability was measured as a percentage of Live/dead blue dye negative cells and the necrotic/apoptotic cells as the percentage of Annexin V^+ cells.

References:

1. Gong H, Sun L, Chen B, Han Y, Pang J, Wu W*, et al.* Evaluation of candidate reference genes for RT-qPCR studies in three metabolism related tissues of mice after caloric restriction. Sci Rep **2016**;6:38513 doi 10.1038/srep38513.
2. Ayoubi HA, Mahjoubi F, Mirzaei R. Investigation of the human H3.3B (H3F3B) gene expression as a novel marker in patients with colorectal cancer. J Gastrointest Oncol **2017**;8(1):64-9 doi 10.21037/jgo.2016.12.12.
3. Tratwal J, Follin B, Ekblond A, Kastrup J, Haack-Sorensen M. Identification of a common reference gene pair for qPCR in human mesenchymal stromal cells from different tissue sources treated with VEGF. BMC Mol Biol **2014**;15:11 doi 10.1186/1471-2199-15-11.
4. Walczak-Drzewiecka A, Salkowska A, Ratajewski M, Dastych J. Epigenetic regulation of CD34 and HIF1A expression during the differentiation of human mast cells. Immunogenetics **2013**;65(6):429-38 doi 10.1007/s00251-013-0695-8.
5. Cruse G, Kaur D, Leyland M, Bradding P. A novel FcepsilonRIbeta-chain truncation regulates human mast cell proliferation and survival. FASEB J **2010**;24(10):4047-57 doi 10.1096/fj.10-158378.
6. Levast B, Barblu L, Coutu M, Prevost J, Brassard N, Peres A*, et al.* HIV-1 gp120 envelope glycoprotein determinants for cytokine burst in human monocytes. PLoS One **2017**;12(3):e0174550 doi 10.1371/journal.pone.0174550.
7. Coulthard LG, Hawksworth OA, Li R, Balachandran A, Lee JD, Sepehrband F*, et al.* Complement C5aR1 Signaling Promotes Polarization and Proliferation of Embryonic Neural Progenitor Cells through PKCzeta. J Neurosci **2017**;37(22):5395-407 doi 10.1523/JNEUROSCI.0525-17.2017.
8. Mogilenko DA, Kudriavtsev IV, Trulioff AS, Shavva VS, Dizhe EB, Missyul BV*, et al.* Modified low density lipoprotein stimulates complement C3 expression and secretion via liver X receptor and Toll-like receptor 4 activation in human macrophages. J Biol Chem **2012**;287(8):5954-68 doi 10.1074/jbc.M111.289322.
9. Hosokawa M, Klegeris A, Maguire J, McGeer PL. Expression of complement messenger RNAs and proteins by human oligodendroglial cells. Glia **2003**;42(4):417-23 doi 10.1002/glia.10234.
10. Chmilewsky F, Jeanneau C, Laurent P, About I. Pulp fibroblasts synthesize functional complement proteins involved in initiating dentin-pulp regeneration. Am J Pathol **2014**;184(7):1991-2000 doi 10.1016/j.ajpath.2014.04.003.
11. Subramanian A, Tamayo P, Mootha VK, Mukherjee S, Ebert BL, Gillette MA*, et al.* Gene set enrichment analysis: a knowledge-based approach for interpreting genome-wide expression profiles. Proc Natl Acad Sci U S A **2005**;102(43):15545-50 doi 10.1073/pnas.0506580102.
